# Supplementary material for: A synergistic strategy to develop photostable and bright dyes with long Stokes shift for nanoscopy
Source: Nat Commun. 2022 Apr 27;13:2264. doi: 10.1038/s41467-022-29547-3 (PMC9046415; doi:10.1038/s41467-022-29547-3)
Supplement: Supplementary file 1 — Supplementary Information [file 41467_2022_29547_MOESM1_ESM.pdf]

Supplementary information

A synergistic strategy to develop photostable and bright dyes with long Stokes shift for nanoscopy

Gangwei Jiang[1,§], Tian-Bing Ren[1,§], Elisa D'Este[2], Mengyi Xiong[1], Bin Xiong[1], Kai Johnsson[3,4], Xiao-Bing Zhang[1], Lu Wang[3,5,*], Lin Yuan[1,*]

1 State Key Laboratory of Chemo/Biosensing and Chemometrics, College of Chemistry and Chemical Engineering, Hunan University, Changsha, 410082, China.

2 Optical Microscopy Facility, Max Planck Institute for Medical Research, Heidelberg, 69120, Germany.

3 Department of Chemical Biology, Max Planck Institute for Medical Research, Heidelberg, 69120, Germany.

4 Institute of Chemical Sciences and Engineering, École Polytechnique Fédérale de Lausanne (EPFL), Lausanne, CH-1015, Switzerland.

5 Key Laboratory of Smart Drug Delivery, Ministry of Education, School of Pharmacy, Fudan University, Shanghai, 201203, China.

§ These authors contributed equally: G. J., T. -B. R..

Correspondence and requests for materials should be addressed to L.Y. (email: lyuan@hnu.edu.cn) or L.W. (email: lwangfd@fudan.edu.cn).

Table of content

1. Supplementary Figures and Tables 3-30
2. Synthesis Procedure 31-47
3. Supplementary notes for test and imaging 47-49
4. NMR and HPLC spectroscopy 50-85
5. References 85-86

Supplementary Figures and Tables

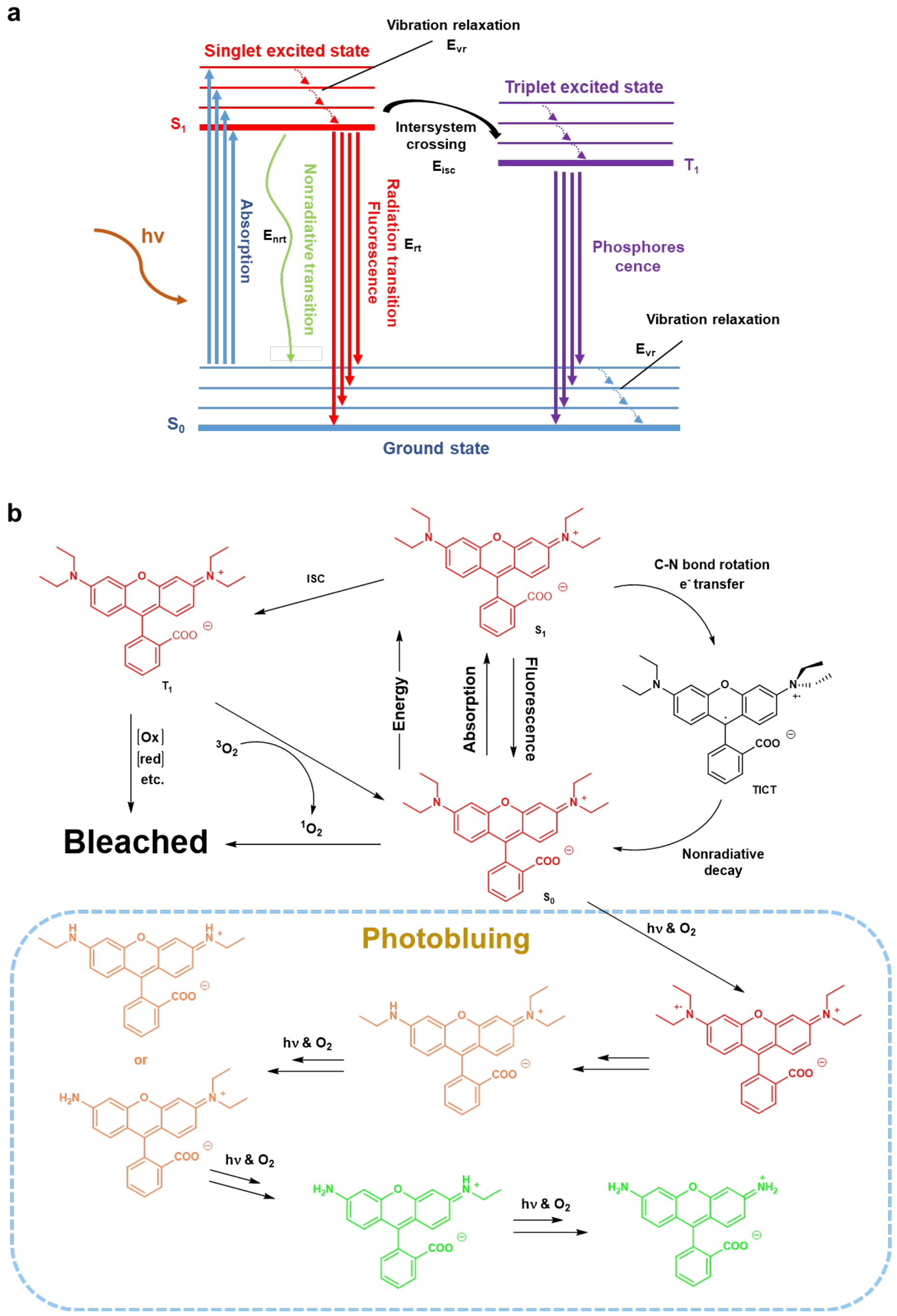

Supplementary Fig. 1 (a) Jabłoński diagram. The Stokes shift is mainly determined by the vibrational relaxation in the excitation state (S_1 state). More energy loss in vibrational relaxation generates the enlarged Stokes shift. When the excited fluorophore relaxes to the ground state, the energy can be released in two ways: radiation transition (fluorescence) and non-radiative transition. The increased energy in the brightness could be from the decreased non-radiative transition. (b) The process of twisted internal charge transfer (TICT) and general mechanisms for the fluorophore photobleaching and photobluing[1, 2, 3].

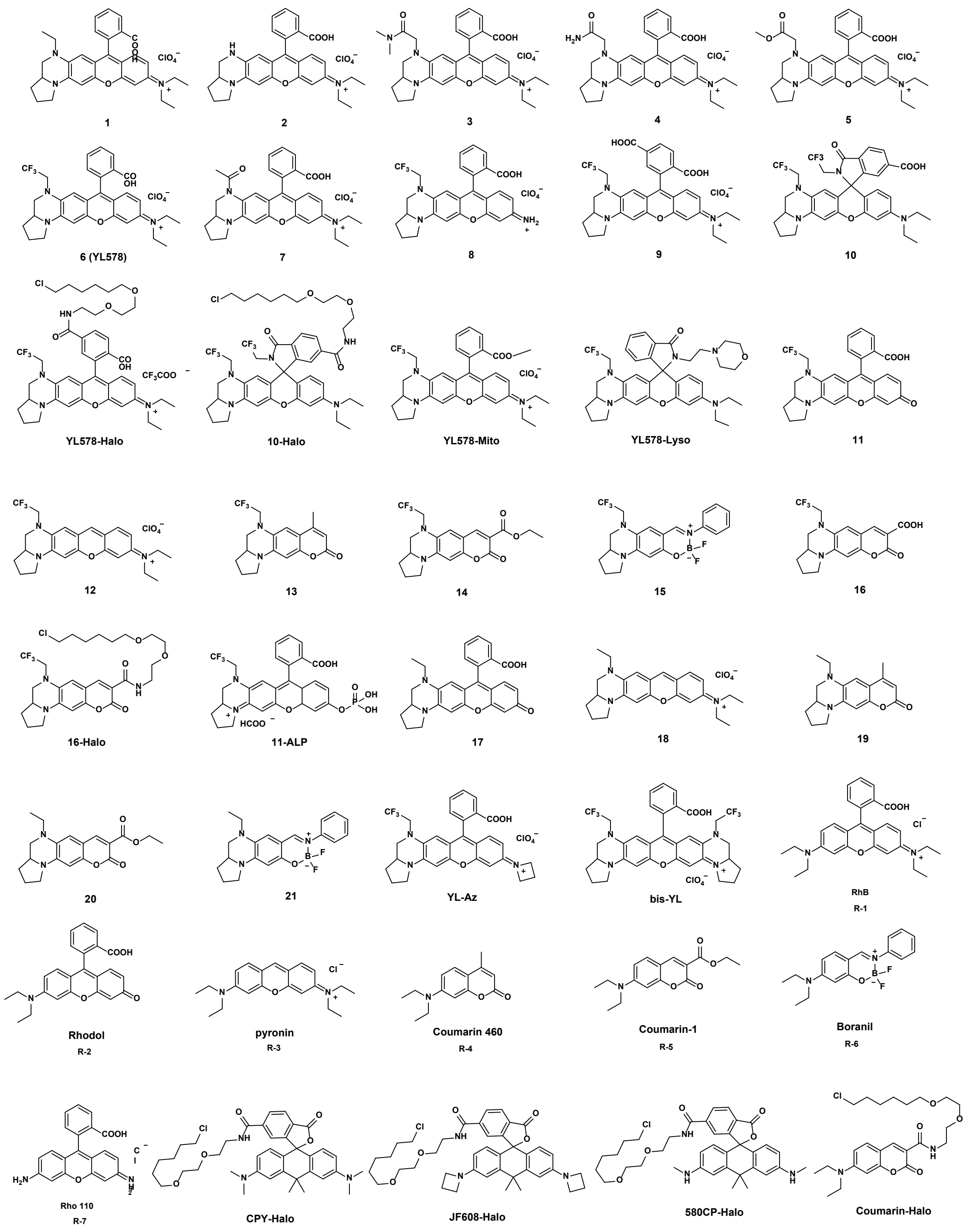

Supplementary Fig. 2 Structures of synthetic and reference fluorophores and probes.

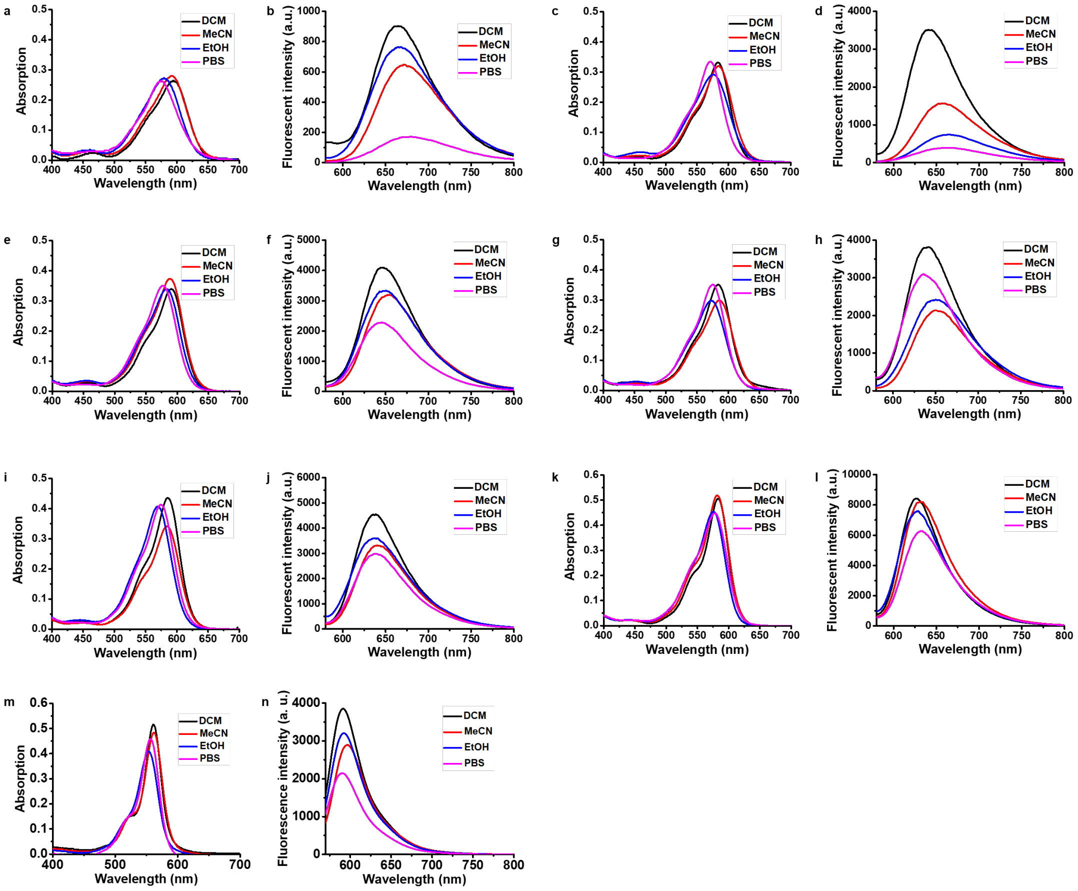

Supplementary Fig. 3 Absorption and emission spectra of **1** (a, b), **2** (c, d), **3** (e, f), **4** (g, h), **5** (i, j), **6** (k, l), **7** (m, n) (5 μM) in various solvents (CH_2Cl_2 (containing 0.1% TFA), MeCN (Containing 0.1% TFA), EtOH, PBS buffer (25 mM)) at 25°C.

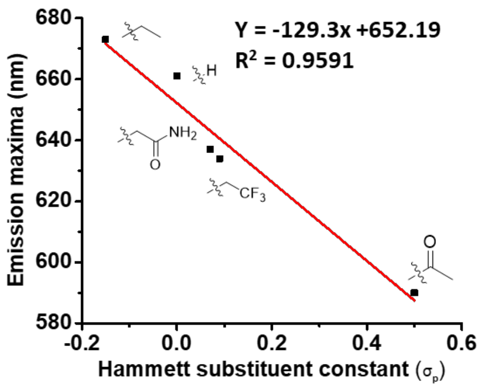

Supplementary Fig. 4 Correlation of emission maxima of dye **1-7** versus Hammett constants (σ_p) of the substituents at the quinoxaline[4].

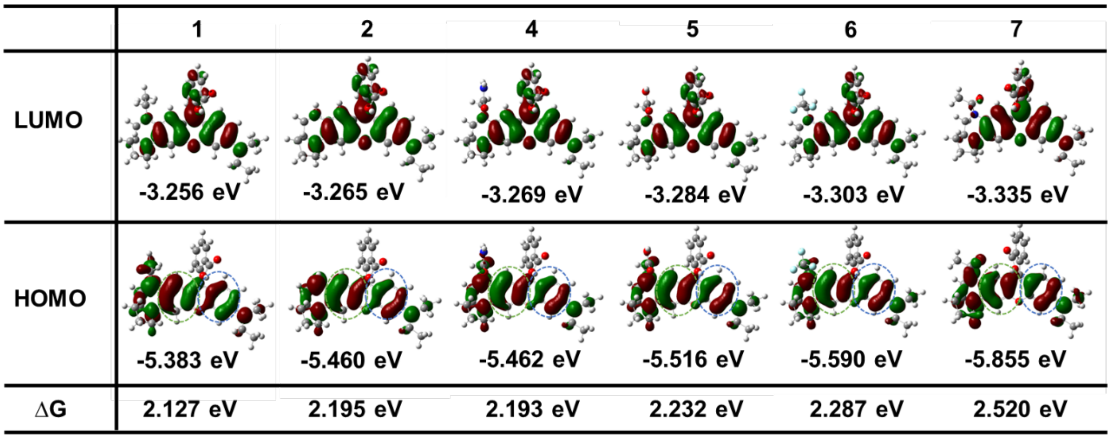

Supplementary Fig. 5 DFT optimized molecular orbital plots (LUMO and HOMO) of **1-7**.

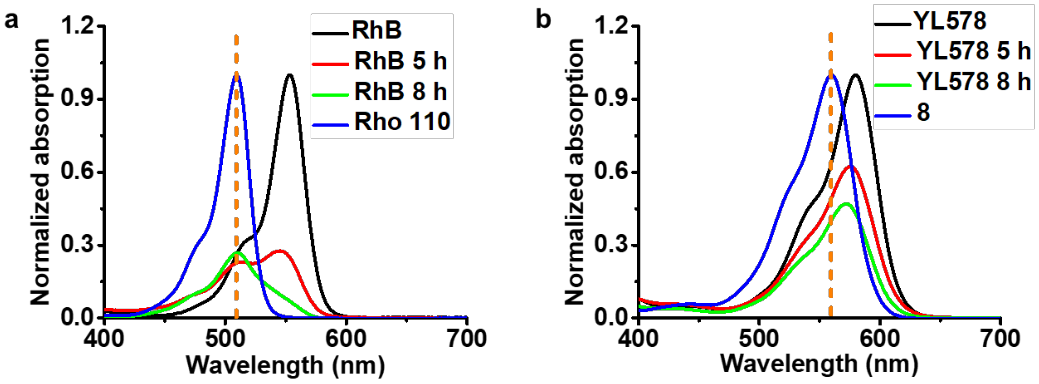

Supplementary Fig. 6 Normalized absorption spectra of **YL578** (b, 100 μM) and RhB (a, 100 μM) as a function of irradiation time in EtOH containing 0.1% TFA. Laser parameters: 530 nm, 1 W. Normalized absorption spectra of Rho 110 and **8** in EtOH containing 0.1% TFA was added to a and b, respectively.

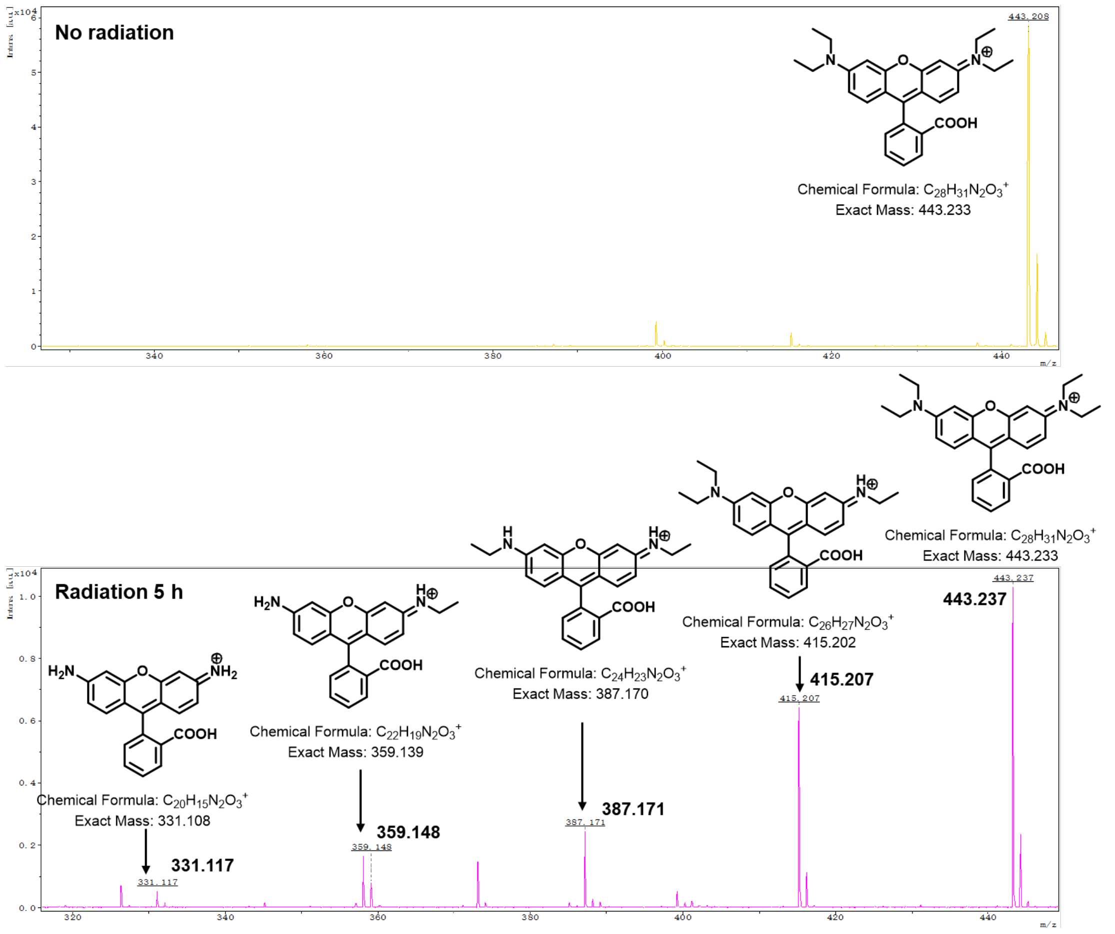

Supplementary Fig. 7 The MALDI-TOF/MS spectra of Rhodamine B in the absence or presence of radiation (5 h). Laser parameters: 530 nm, 1 W. EtOH containing 0.1% TFA.

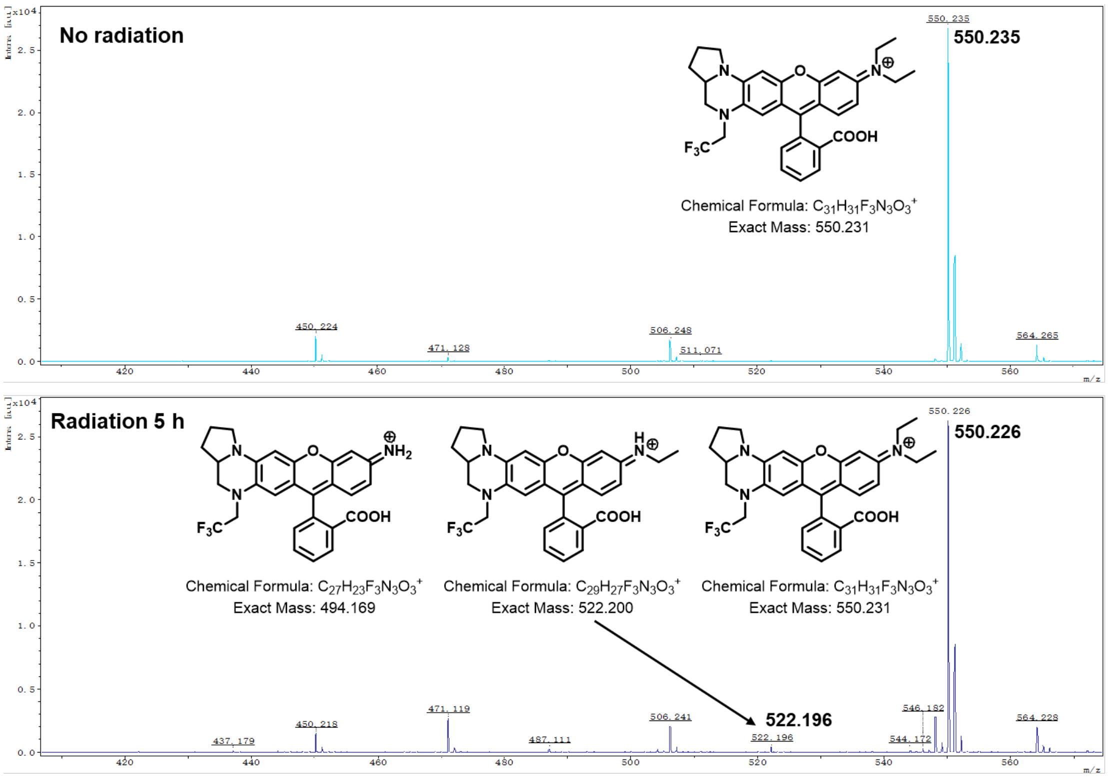

Supplementary Fig. 8 The MALDI-TOF/MS spectra of **YL578** in the absence or presence of radiation (5 h). Laser parameters: 530 nm, 1 W. EtOH containing 0.1% TFA.

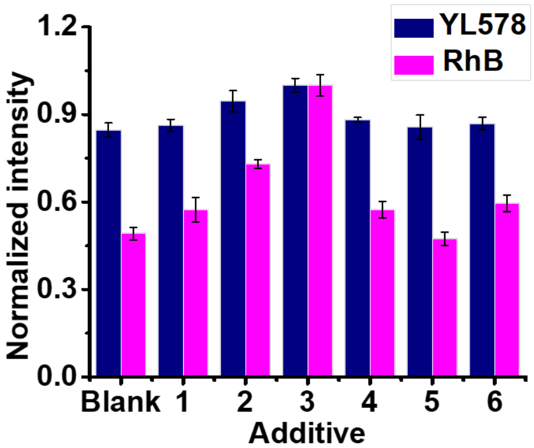

Supplementary Fig. 9 Normalized fluorescence maximum intensities of **YL578** and RhB in PBS buffer solution with abundant proteins (1: 10 mg/mL BSA; 2: 30 mg/mL BSA; 3: 50 mg/mL BSA; 4: 25 mg/mL CRP; 5: 1 mg/mL Gox; 6: 90 µL/mL Serum). Error bars, ± s.e.m. n = 3.

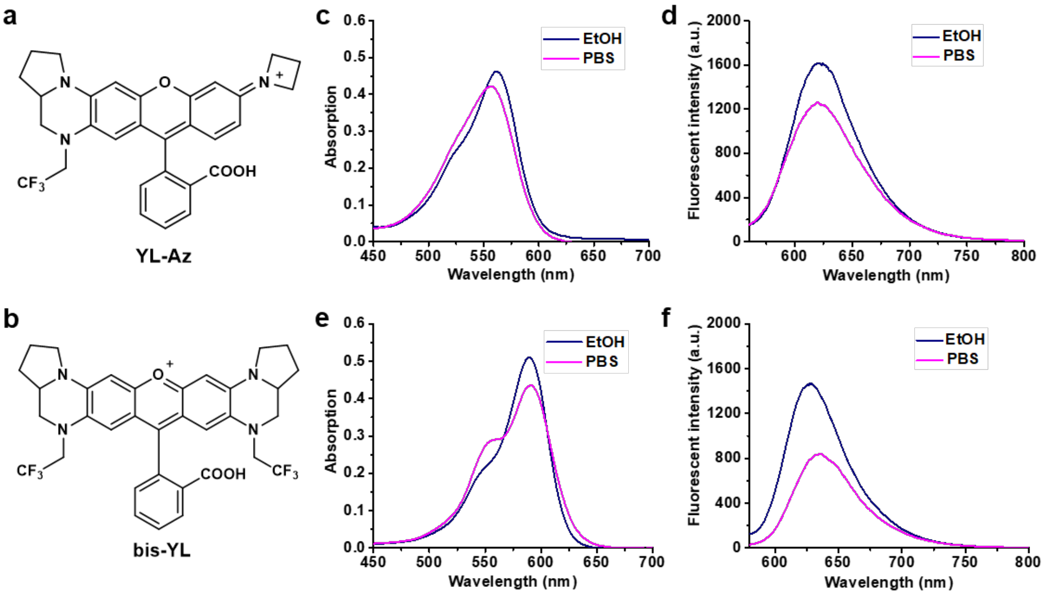

g

YL578	λ_{Abs}/nm	λ_{Em}/nm	Stokes shift/nm	φ	$\varepsilon/M^{-1}\ cm^{-1}$	$\varepsilon\times\varphi/M^{-1}cm^{-1}$
EtOH	579	628	49	0.85	91000	77400
PBS	578	634	56	0.74	89700	66400
YL-Az	λ_{Abs}/nm	λ_{Em}/nm	Stokes shift/nm	φ	$\varepsilon/M^{-1}\ cm^{-1}$	$\varepsilon\times\varphi/M^{-1}cm^{-1}$
EtOH	562	625	63	0.77	86100	66300
PBS	556	623	67	0.69	80300	55400
bis-YL	λ_{Abs}/nm	λ_{Em}/nm	Stokes shift/nm	φ	$\varepsilon/M^{-1}\ cm^{-1}$	$\varepsilon\times\varphi/M^{-1}cm^{-1}$
EtOH	590	629	39	0.87	112000	97400
PBS	590	635	45	0.56	100400	56000
RhB	λ_{Abs}/nm	λ_{Em}/nm	Stokes shift/nm	φ	$\varepsilon/M^{-1}\ cm^{-1}$	$\varepsilon\times\varphi/M^{-1}cm^{-1}$
EtOH	553	577	24	0.65	115000	74800
PBS	554	578	24	0.31	111000	34400
JF549	λ_{Abs}/nm	λ_{Em}/nm	Stokes shift/nm	φ	$\varepsilon/M^{-1}\ cm^{-1}$	$\varepsilon\times\varphi/M^{-1}cm^{-1}$
EtOH	538	561	23	0.89	115000	102000
PBS	549	571	22	0.88	99000	87100

Supplementary Fig. 10 (a, b) Structures of **YL-Az** and **bis-YL**. (c-f) Absorption and emission spectra of **YL-Az** (c, d) and **bis-YL** (e, f). (g) Photophysical properties of **YL578**, **YL-Az**, **bis-YL**, RhB and JF549 in EtOH and PBS buffer solution.

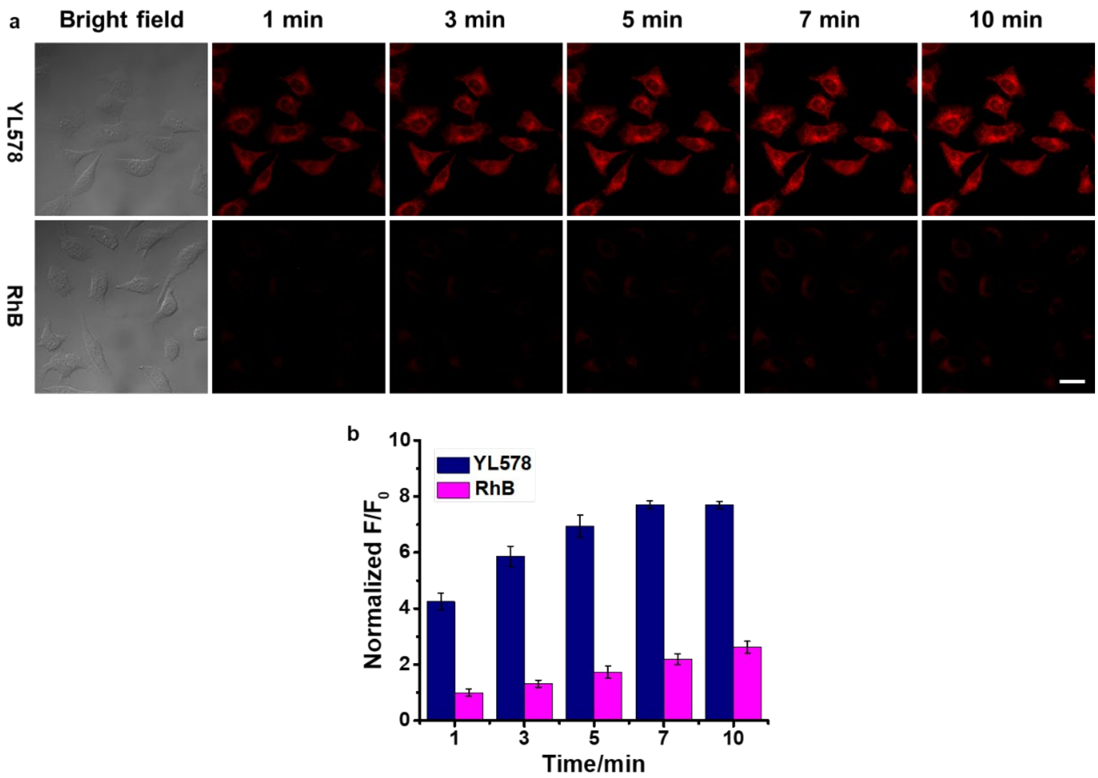

Supplementary Fig. 11 Staining performance of **YL578** and RhB. (a) Confocal fluorescence images of live HeLa cells incubated with **YL578** or RhB (5.0 μM) as a function of incubation time (1–10 min). (b) Quantification of the relative mean fluorescence intensities of **YL578** and RhB in live HeLa cells. The fluorescence intensities were normalized to that of the cells incubated with RhB for 0 min (F_0). Scale bar = 20 μm. λ_{ex} = 561 nm, detection range 585–675 nm. Error bars, ± s.e.m. from about 10 cells.

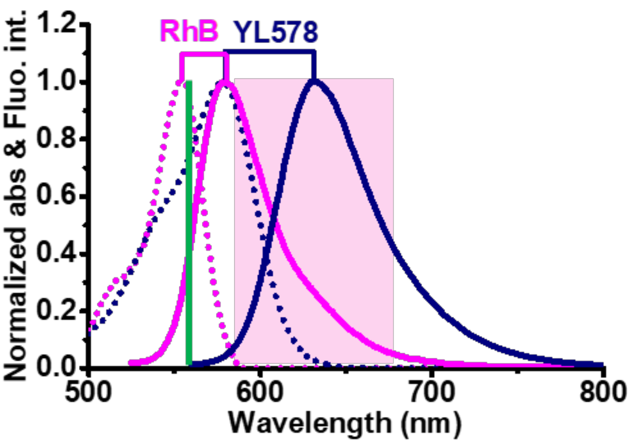

Supplementary Fig. 12 Normalized absorption and emission spectra of **YL578** and RhB. The green line and light pink area respectively mark the excitation wavelength (560 nm) and the bandpass of the confocal filter (585-675 nm) for cell imaging.

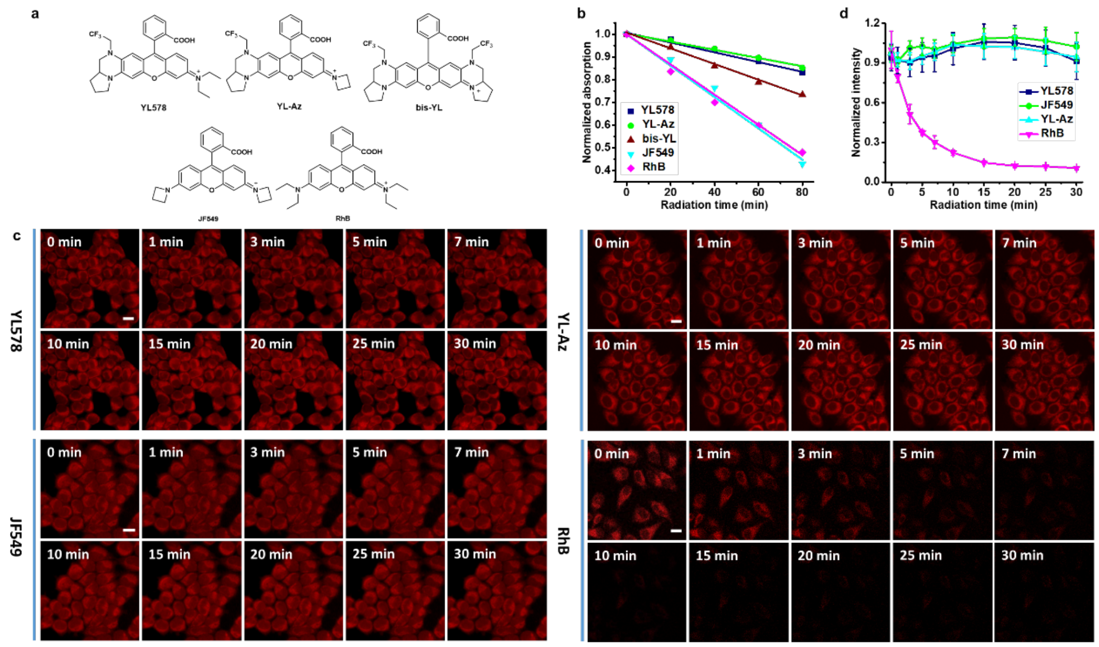

Supplementary Fig. 13 Comparison of the photostability of **YL578**, **YL-Az**, **bis-YL**, JF549 and RhB. (a) Structures of **YL578**, **YL-Az**, **bis-YL**, JF549 and RhB. (b) Absorption at λ_{max} of **YL578**, **YL-Az**, **bis-YL**, JF549 and RhB were plotted as a function of irradiation time with a laser (1 W) at 530 nm. Solution concentrations were adjusted to be comparable to one another in terms of optical density at 530 nm. (c) Confocal fluorescence images of live HeLa cells incubated with 5.0 µM **YL578,** or **YL-Az,** or JF548, or RhB under continuous irradiation at 561 nm for 30 min. HeLa cells in each group were examined in 3 independent experiments separately. (d) Normalized fluorescence intensities of live HeLa cells in **c**. The fluorescence intensities were normalized to that of the cells incubated with **YL578,** or **YL-Az,** or JF549, or RhB for 0 min (F_0) before irradiation at 561 nm. The inconsistent performance of JF549 may result from the difference between organic solvent and intracellular environment. Scale bar = 20 µm. λ_{ex} = 561 nm, detection range 585–675 nm. Error bars, ± s.e.m.

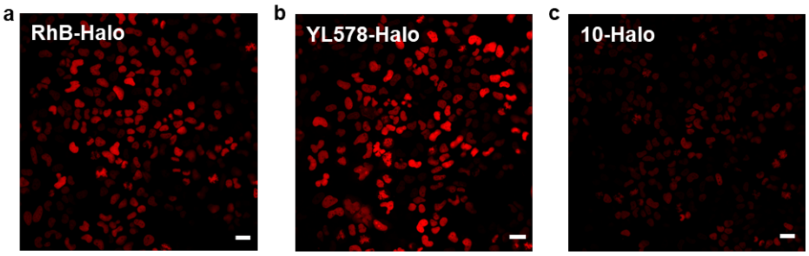

Supplementary Fig. 14 Confocal images of living H2B-Halo-expressed HeLa cells treated with RhB-Halo (a), **YL578-Halo** (b), and **10-Halo** (c). HeLa cells were incubated with 250 nM probes for 6 h, washed once with phenol-red-free DMEM and incubated for 1 h prior to cell imaging. HeLa cells in each group were examined in 3 independent experiments separately. Scale bar = 50 nm. λ_{ex} = 561 nm, detection range 585–675 nm.

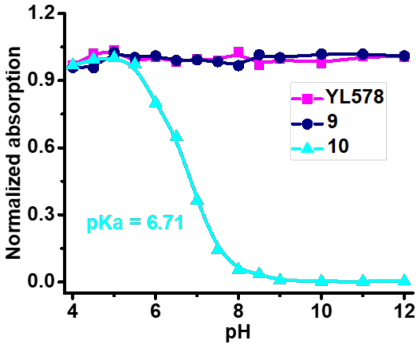

Supplementary Fig. 15 Normalized absorbance of 5 μM **YL578** derivatives (**YL578**, 9 and 10) in PBS buffer (25 mM) with different pH (4-12). The absorbance was normalized to the maximum absorbance of each dyes.

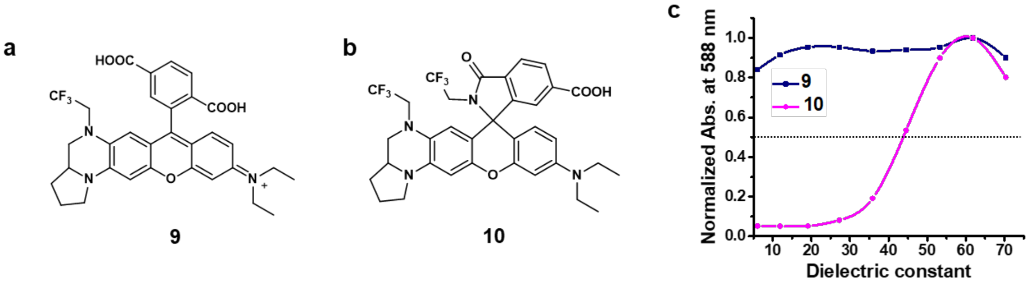

Supplementary Fig. 16 Comparison of **9** and **10**. (a, b) Structures of **9** and **10**. (c) Normalized absorbance at 588 nm in zwitterionic form of 5 μM **9** and **10** in water-dioxane mixtures (v/v, 10/90-90/10) as a function of dielectric constant. The absorbance was normalized to the maximum absorbance of **9**.

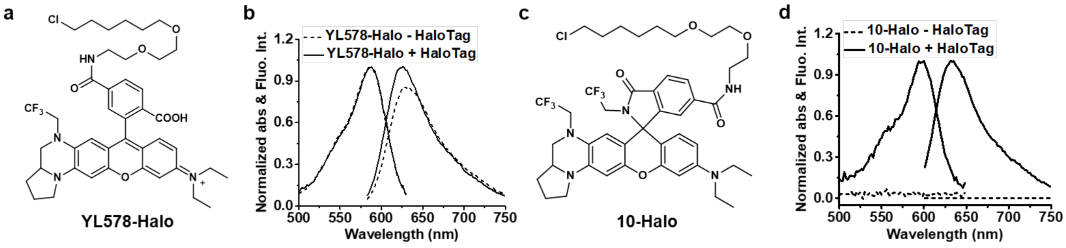

Supplementary Fig. 17 *In vitro* response of **YL578-Halo** and **10-Halo** towards HaloTag. (a, c) Structures of **YL578-Halo** and **10-Halo.** (b, d) Normalized absorption and emission spectra of **YL578-Halo** (b) and **10-Halo** (d) measured in the presence (+ protein) and absence (- protein) of HaloTag protein after 1 h incubation.

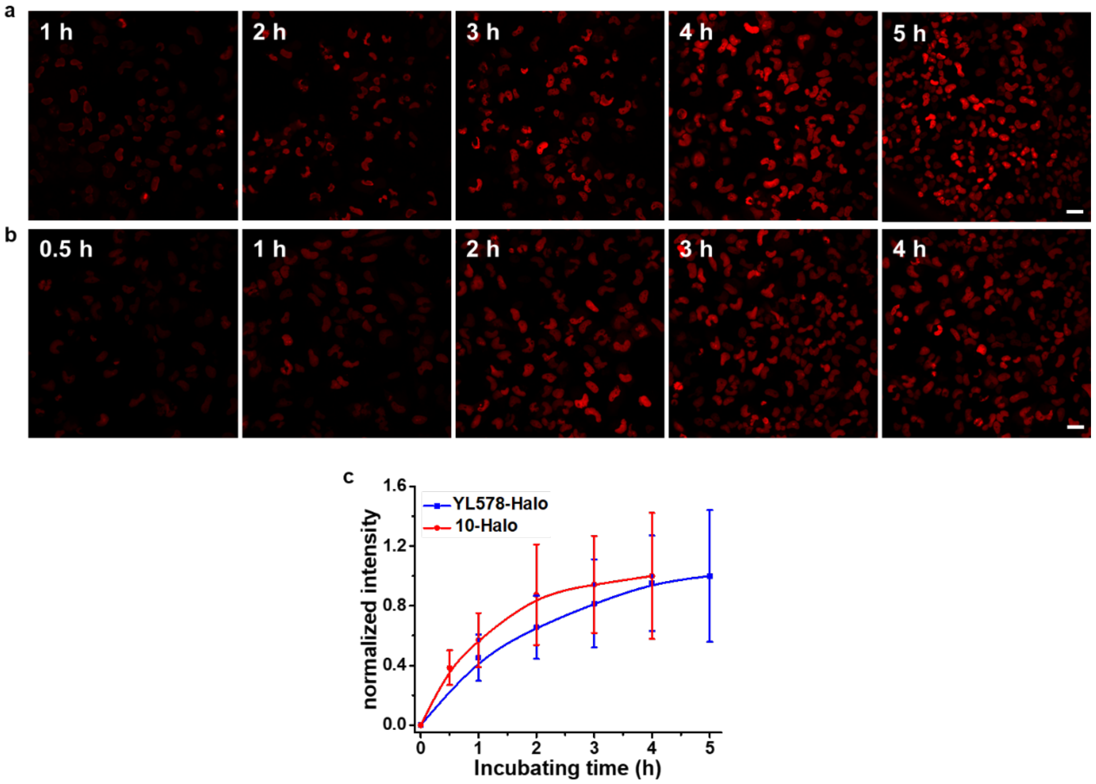

Supplementary Fig. 18 Intracellular protein labeling with **YL578-Halo** and **10-Halo** as a function of time. (a, b) Time-dependent confocal images of live HeLa H2B-Halo-expressing cells labeled with 250 nM **YL578-Halo** (a) or **10-Halo** (b). (c) Normalized fluorescence intensities of live HeLa cells incubated with **YL578-Halo** or **10-Halo** at different time points. The fluorescence intensities were normalized to the intensity of cells treated with **YL578-Halo** for 4 h or that of **10-Halo** for 5 h. Scale bar = 50 μm. λ_{ex} = 561 nm, detection range 585–675 nm. Error bars, ± s.e.m. from about 50 cells.

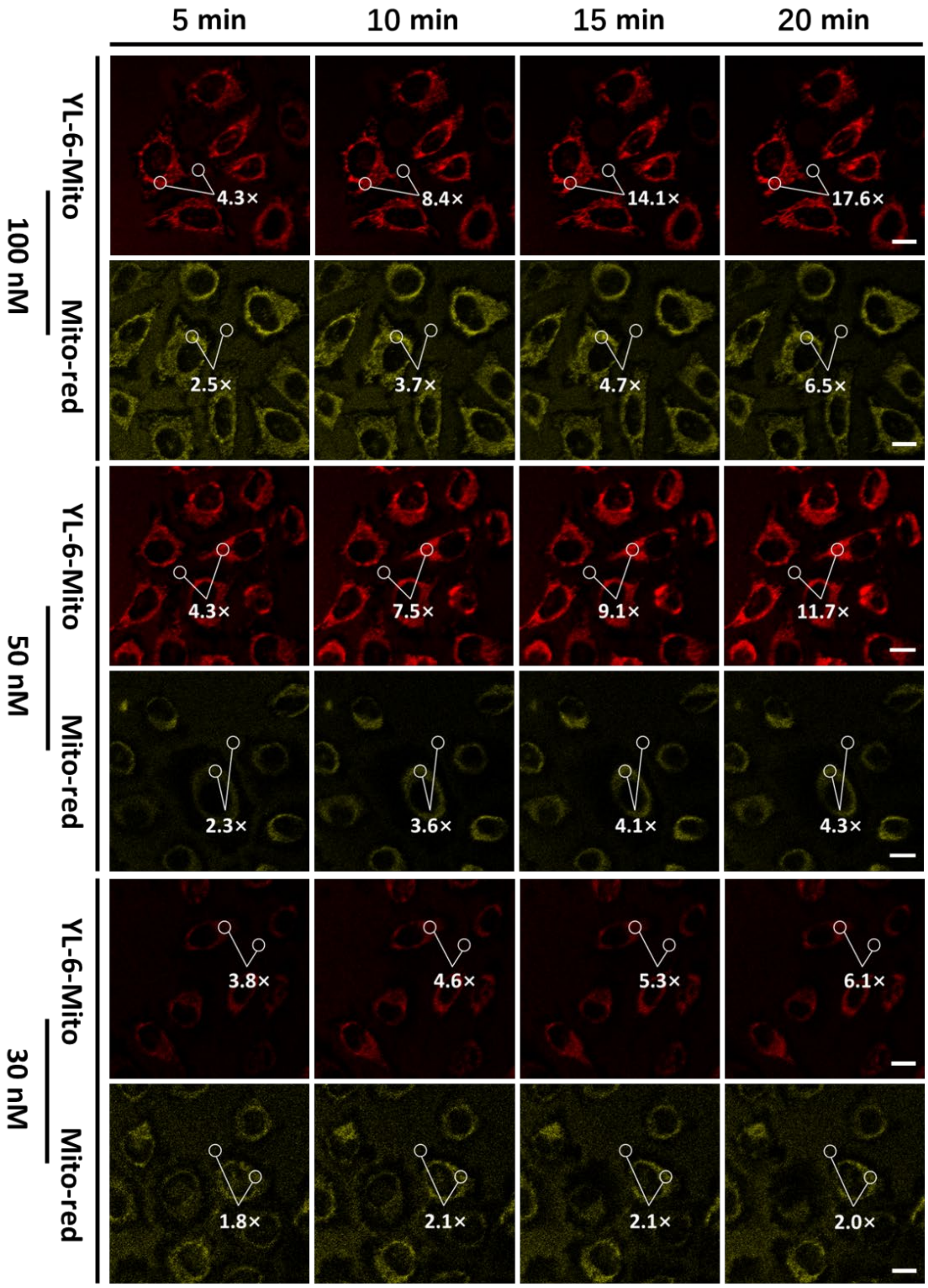

Supplementary Fig. 19 Staining performance of **YL578-Mito** and Mito-Red in live-cell imaging. Confocal fluorescent images of live HeLa cells incubated with **YL578-Mito** or Mito-red at different concentrations (100, 50, and 30 nM). Scale bar = 20 μm. λ_{ex} = 561 nm, detection range 585–675 nm.

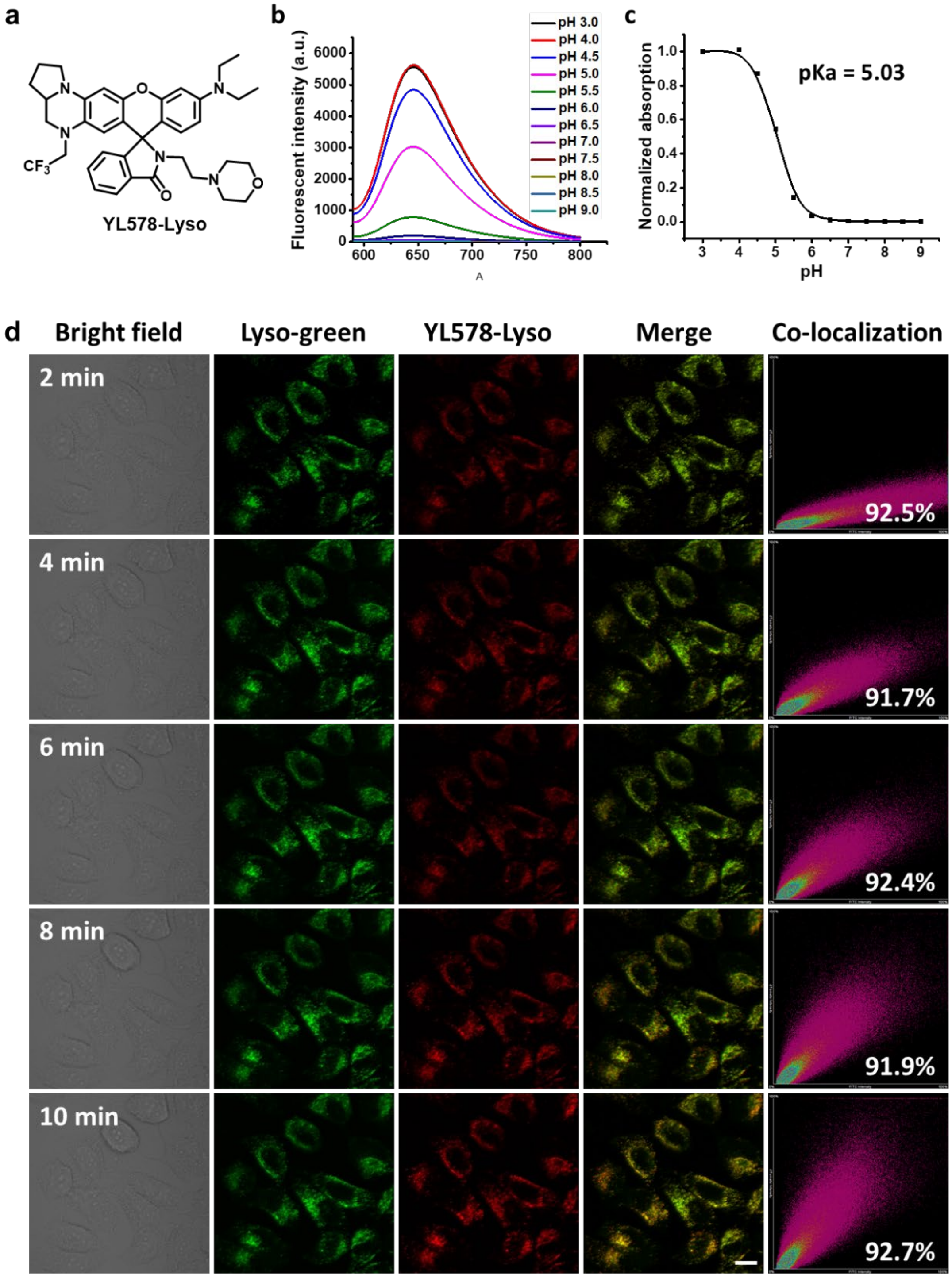

Supplementary Fig. 20 Response of **YL578-Lyso** to pH. (a) Structure of **YL578-Lyso**. (b) Fluorescence spectra of **YL578-Lyso** (5 μM) at different pH values in PBS buffer (25 mM). (c) pH titration curve was plotted by **YL578-Lyso** fluorescence intensities at 635 nm as a function of pH. (d) Confocal fluorescent images of live HeLa cells incubated with **YL578-Lyso** (500 nM) for different time periods. HeLa cells were pre-incubated with Lyso-Green (500 nM) for 10 min before the addition of **YL578-Lyso**. Scale bar = 20 μm. λ_{ex} = 561 nm, detection range 585–675 nm.

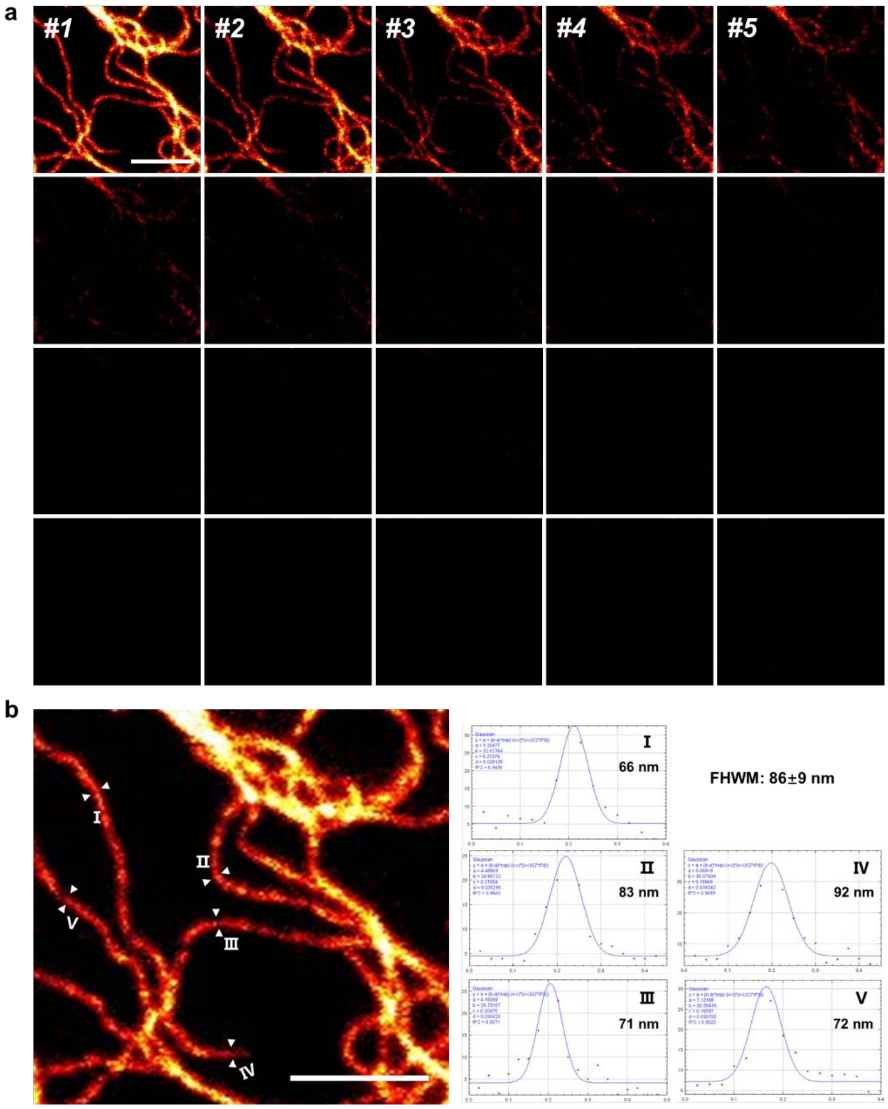

Supplementary Fig. 21 (a) Multiframe STED images of fixed U-2 OS stably expressing vimentin-HaloTag-expressing cells labeled with 50 nM CPY-Halo for 6 h. (b) The resolution of vimentin filaments in the first frame of graph **a**. The corresponding line profiles drawn perpendicular to the indicated filaments. Counts were averaged over five pixels along the direction of the filament and fitted to the Gaussian function. Resulting full-width at half maximum values are given for each fit, providing an upper boundary for the optical resolution. 20 areas from 5 cells were examined in 2 independent experiments separately. Scale bar = 1 μm.

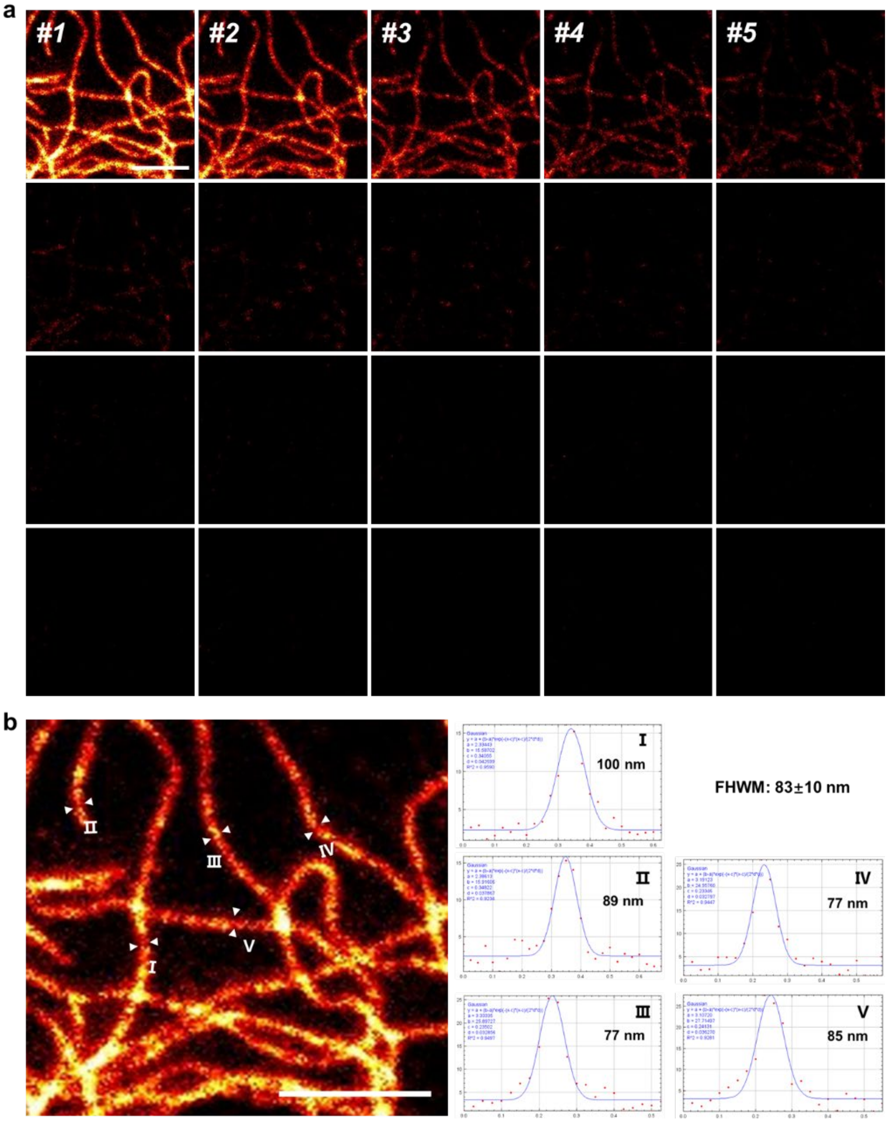

Supplementary Fig. 22 (a) Multiframe STED images of fixed U-2 OS stably expressing vimentin-HaloTag-expressing cells labeled with 50 nM JF608-Halo for 6 h. (b) The resolution of vimentin filaments in the first frame of graph **a**. The corresponding line profiles drawn perpendicular to the indicated filaments. Counts were averaged over five pixels along the direction of the filament and fitted to the Gaussian function. Resulting full-width at half maximum values are given for each fit, providing an upper boundary for the optical resolution. 20 areas from 5 cells were examined. Scale bar = 1 µm.

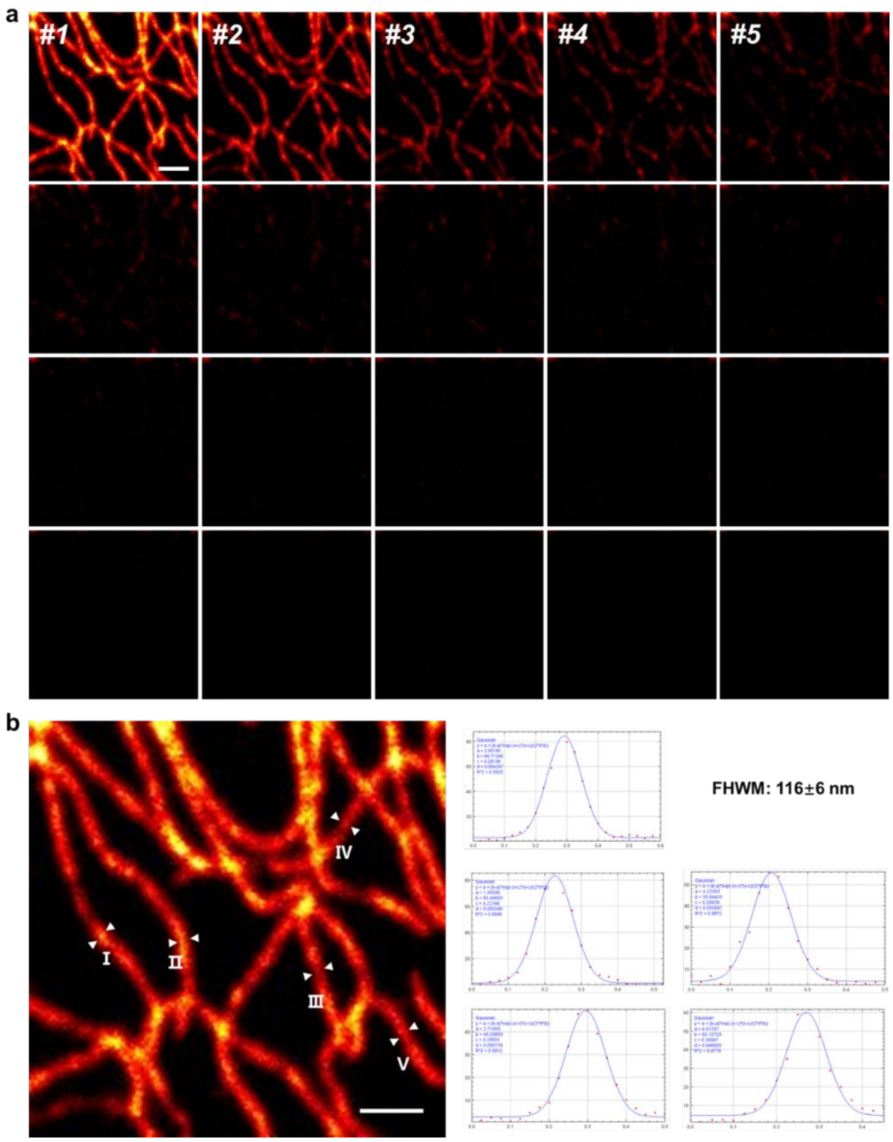

Supplementary Fig. 23 (a) Multiframe STED images of fixed U-2 OS stably expressing vimentin-HaloTag-expressing cells labeled with 50 nM 580CP-Halo for 6 h. (b) The resolution of vimentin filaments in the first frame of graph **a**. The corresponding line profiles drawn perpendicular to the indicated filaments. Counts were averaged over five pixels along the direction of the filament and fitted to the Gaussian function. Resulting full-width at half maximum values are given for each fit, providing an upper boundary for the optical resolution. 20 areas from 5 cells were examined. Scale bar = 1 µm.

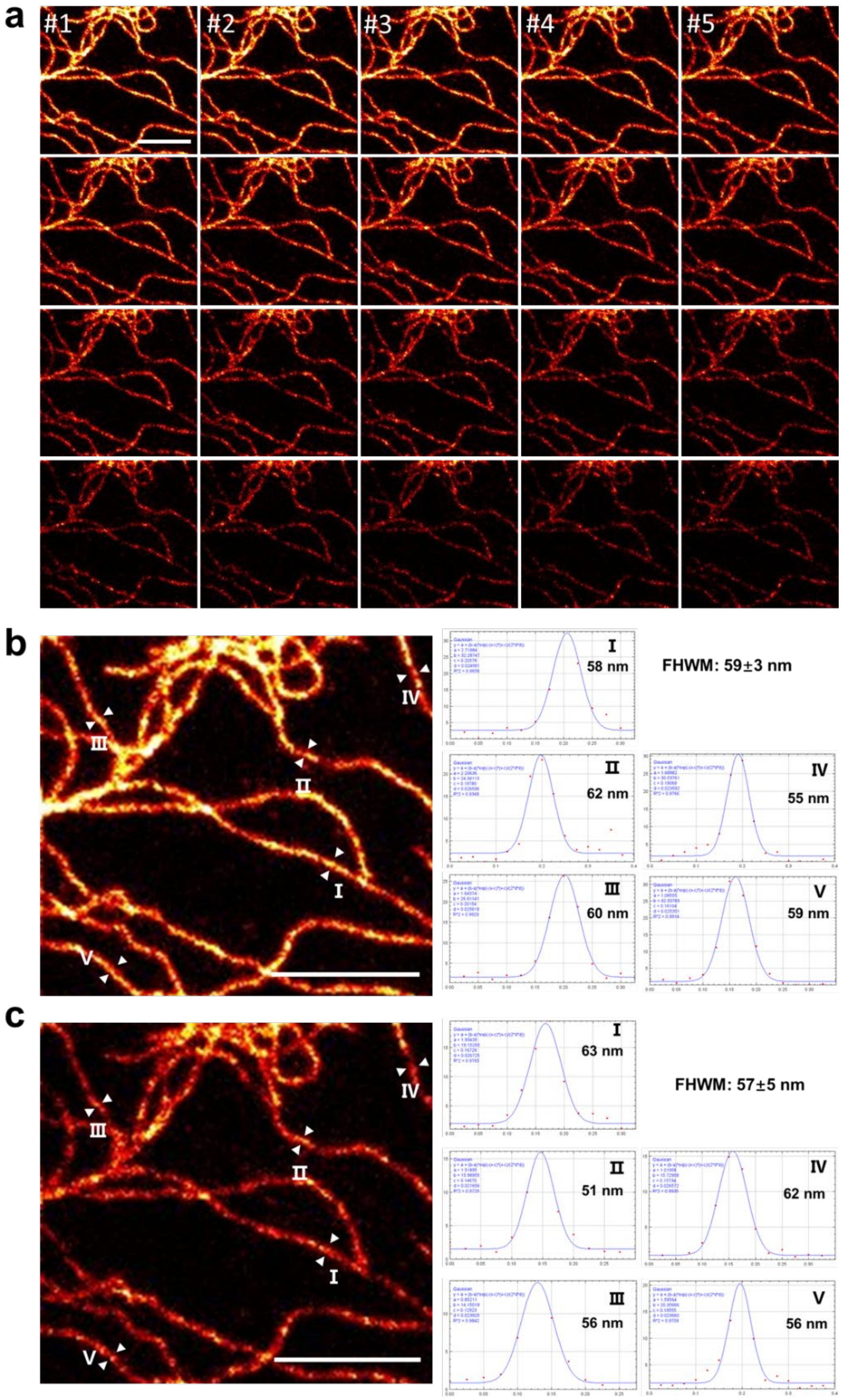

Supplementary Fig. 24 (a) Multiframe STED images of fixed U-2 OS stably expressing vimentin-HaloTag-expressing cells labeled with 50 nM **YL578-Halo** for 6 h. (b, c) The resolution of vimentin filaments in the first (b) and ninth (c) frame. The corresponding line profiles drawn perpendicular to the indicated filaments. Counts were averaged over five pixels along the direction of the filament and fitted to the Gaussian function. Resulting full-width at half maximum values are given for each fit, providing an upper boundary for the optical resolution. 20 areas from 5 cells were examined in 3 independent experiments separately. Scale bar: 1 μm.

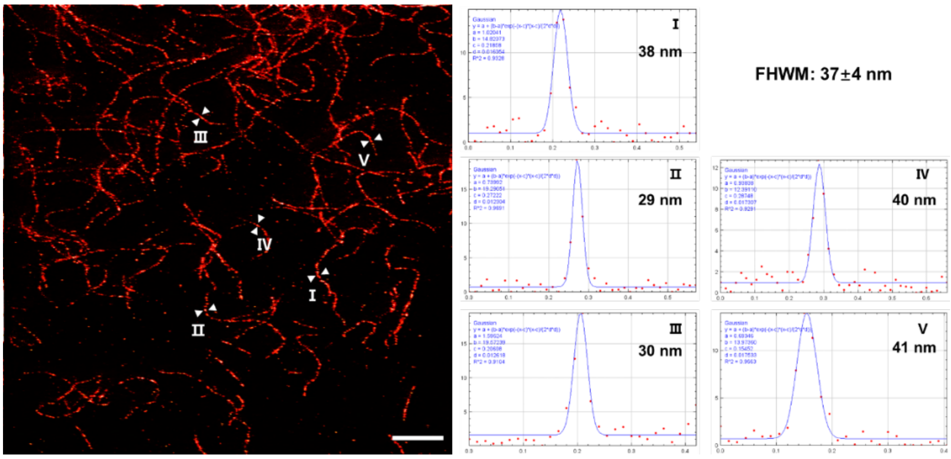

Supplementary Fig. 25 Ultrahigh-resolution STED images of live U-2 OS stably expressing vimentin-HaloTag-expressing cells labeled with 50 nM **YL578-Halo** for 6 h. The resolution of vimentin filaments when optimizing the imaging settings. The corresponding line profiles drawn perpendicular to the indicated filaments. Counts were averaged over five pixels along the direction of the filament and fitted to the Gaussian function. Resulting full-width at half maximum values are given for each fit, providing an upper boundary for the optical resolution. 20 areas from 5 cells were examined. Scale bar: 1 μm.

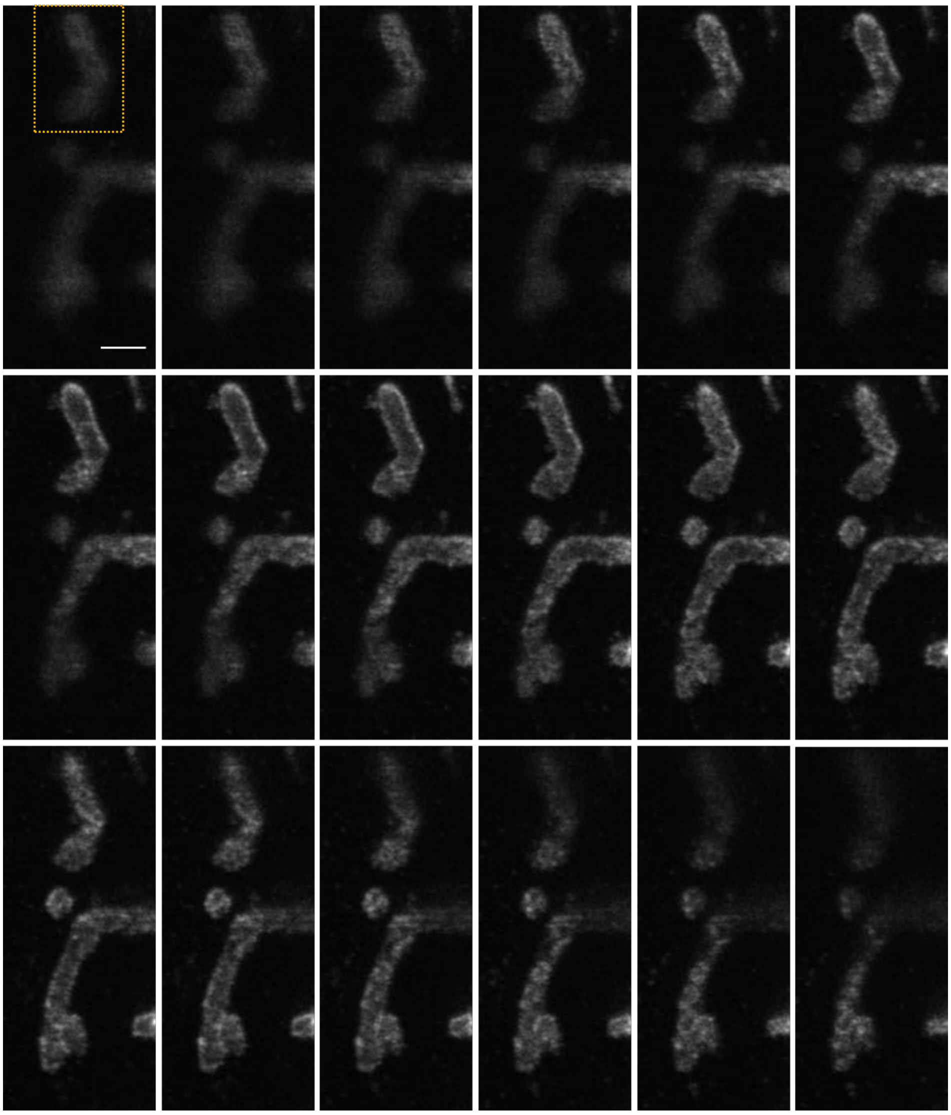

Supplementary Fig. 26 STED images of U-2 OS expressing mitochondrial import receptor Tomm20-HaloTag cells labeled with 50 nM **YL578-Halo** for 6 h. Frames were recorded in sequential *xzy*-scanning mode. STED images of mitochondria in the dashed box were utilized to construct a 3D STED image (Figure 4c). Scale bar = 1 μm.

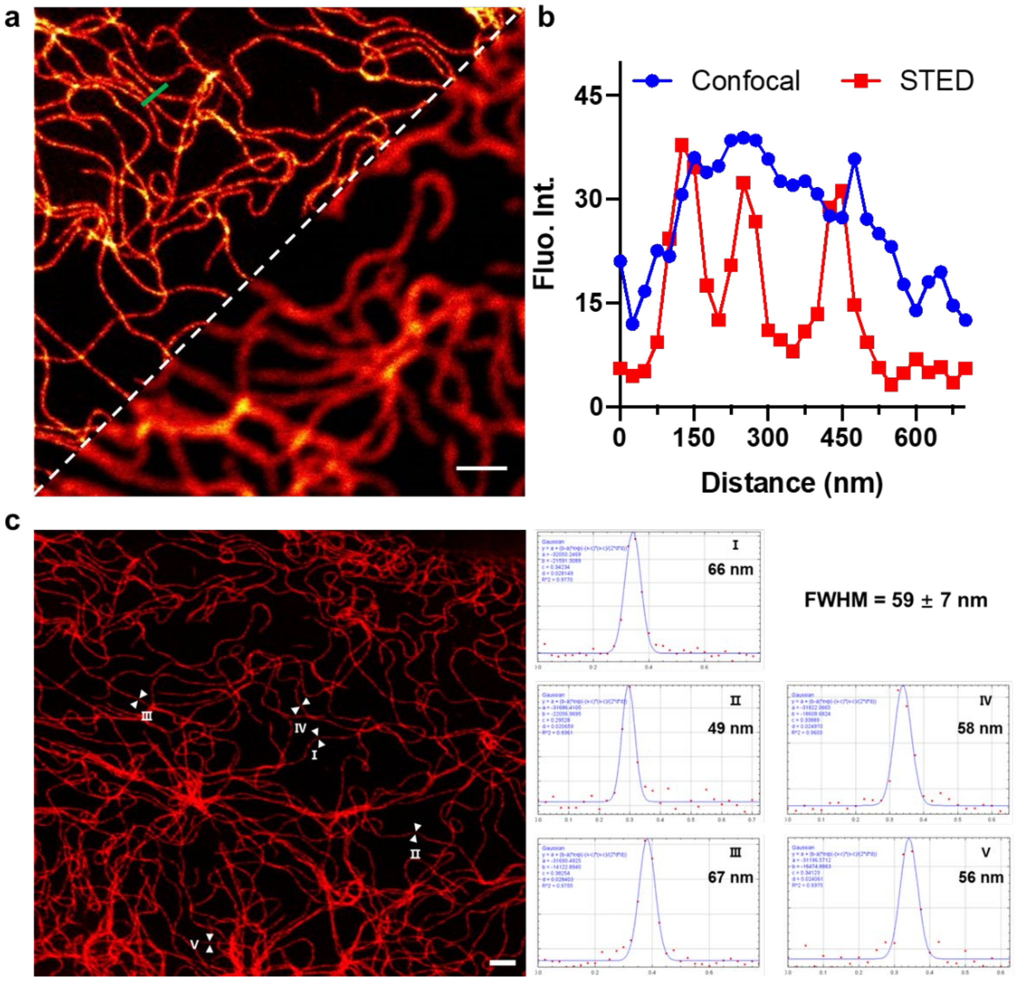

Supplementary Fig. 27 STED images of live U-2 OS cells labeled with **YL578-Halo**. (a) STED images of vimentin filaments in live U-2 OS cells that expressed vimentin-Halo fusion protein after incubation with **YL578-Halo** (50 nM) for 6 h. (b) Plot of line-scan intensity in a (green line) as a function of line length in **a**. (c) The corresponding line profiles drawn perpendicular to the indicated filaments. Counts were averaged over five pixels along the direction of the filament and fitted to the Gaussian function. Resulting full-width at half maximum values are given for each fit, providing an upper boundary for the optical resolution. 20 areas from 5 cells were examined in 3 independent experiments separately. Scale bar = 1 µm.

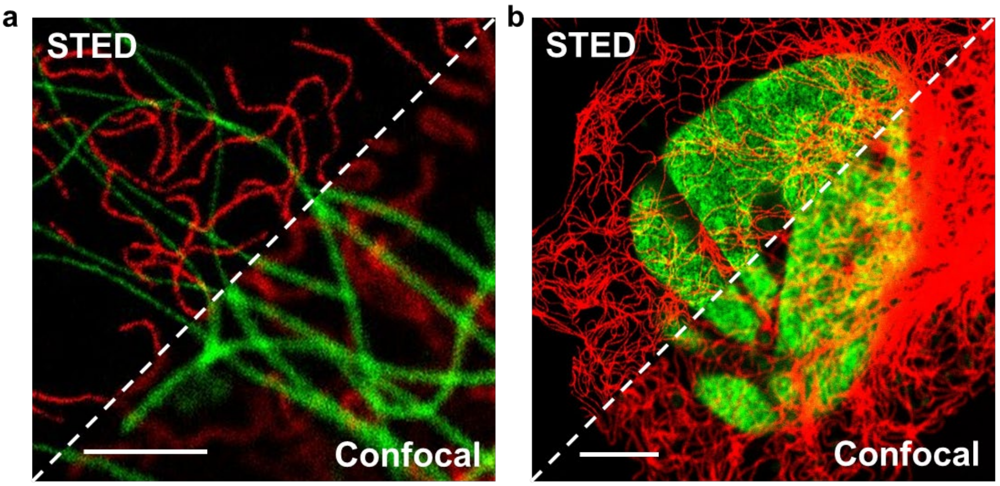

Supplementary Fig. 28 (a) Dual-color confocal and STED images of live U-2 OS Vimentin-HaloTag-expressing cells labeled with 50 nM **YL578-Halo** (red, STED at 775 nm), 500 nM GeR-tubulin (green, STED at 775 nm) Scale bar, 2 μm. (b) Dual-color confocal and STED images of live U-2 OS Vimentin-HaloTag-expressing cells labeled with 50 nM **YL578-Halo** (red, STED at 775 nm), 500 nM SiR-DNA (green, STED at 775 nm) Scale bar, 5 μm.

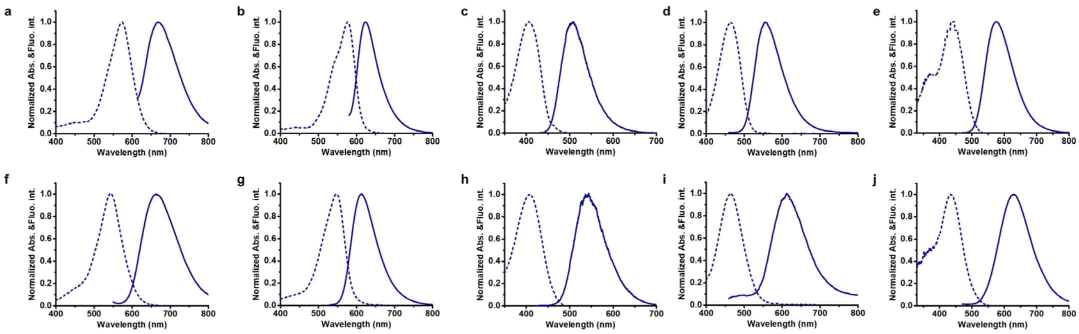

Supplementary Fig. 29 Normalized absorption and emission spectra of **11** (a), **12** (b), **13** (c), **14** (d), **15** (e), **17** (f), **18** (g), **19** (h), **20** (i), **21** (j) (5 μM) in PBS buffer (25 mM) at 25 °C

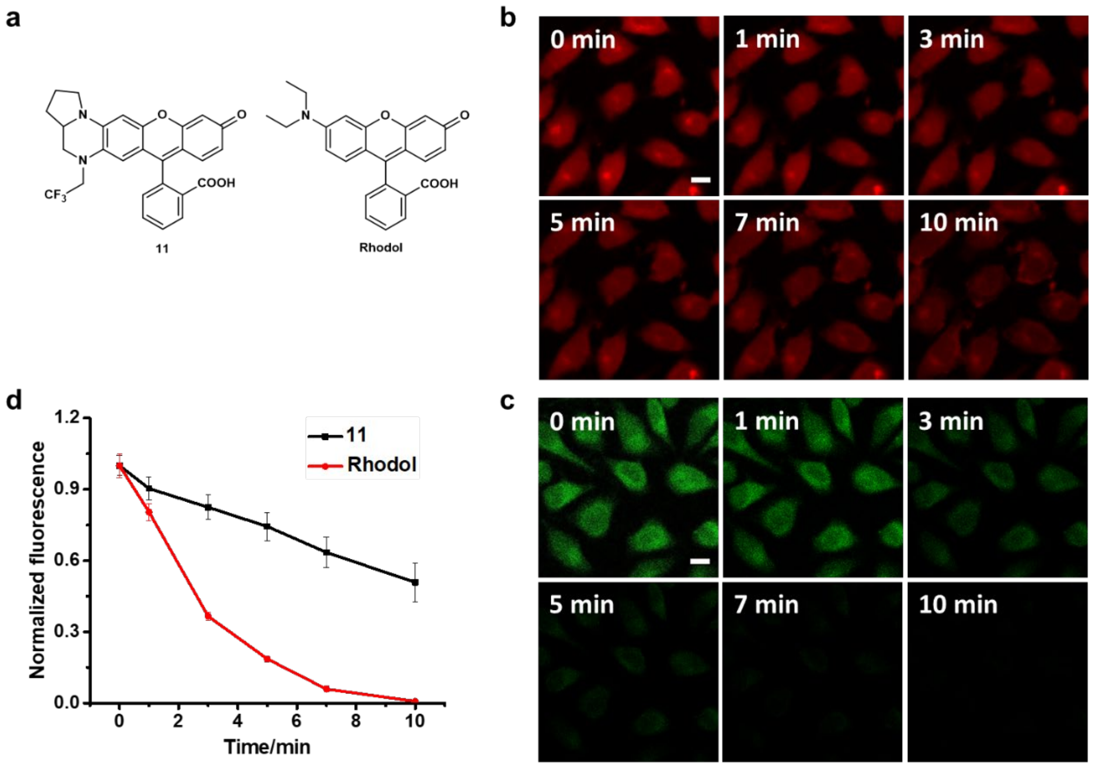

Supplementary Fig. 30 Comparison of **11** and Rhodol (R-2) in live-cell imaging. (a) structures of **11** and Rhodol. (b-c) Confocal fluorescence images of live HeLa cells cultured with **11** (5.0 µM) (b) and Rhodol (5.0 µM) (c) under continuous irradiation for 10 min. (d) Normalized fluorescence intensities of live HeLa cells in **b** and **c**. The fluorescence intensities were normalized to that of the cells incubated with **11** or Rhodol for 0 min (F_0) prior to irradiation. HeLa cells in each group were examined in 3 independent experiments separately. Scale bar = 20 µm. For **11**, λ_{ex} = 561 nm, detection range, 585-675 nm. For Rhodol, λ_{ex} = 488 nm, detection range 500-550 nm. Error bars, ± s.e.m. from about 50 cells.

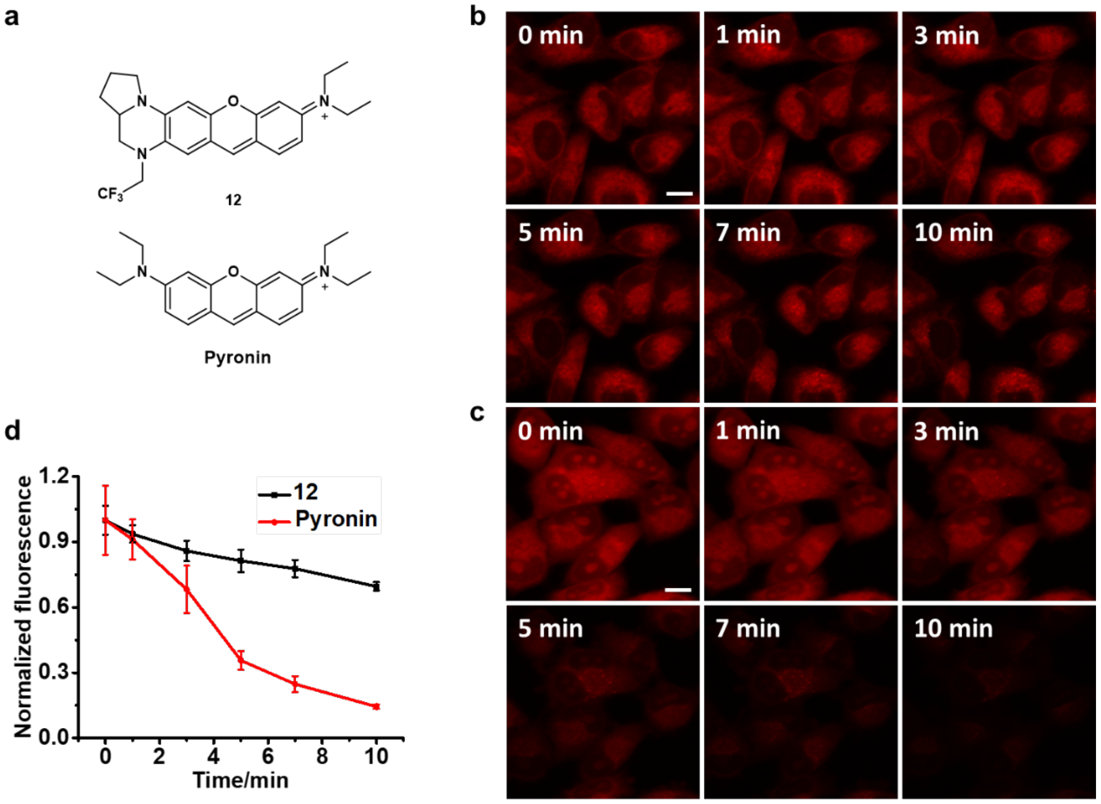

Supplementary Fig. 31 Comparison of **12** and Pyronin (R-3) in live-cell imaging. (a) structures of **12** and Pyronin. (b-c) Confocal fluorescence images of live HeLa cells cultured with **12** (5.0 μM) (b) and Pyronin (5.0 μM) (c) under continuous irradiation for 10 min. (d) Normalized fluorescence intensities of live HeLa cells in **b** and **c**. The fluorescence intensities were normalized to that of the cells incubated with **12** or Pyronin for 0 min (F_0) before irradiation. HeLa cells in each group were examined in 3 independent experiments separately. Scale bar = 20 μm. λ_{ex} = 561 nm, detection range, 585-675 nm. Error bars, ± s.e.m. from about 50 cells.

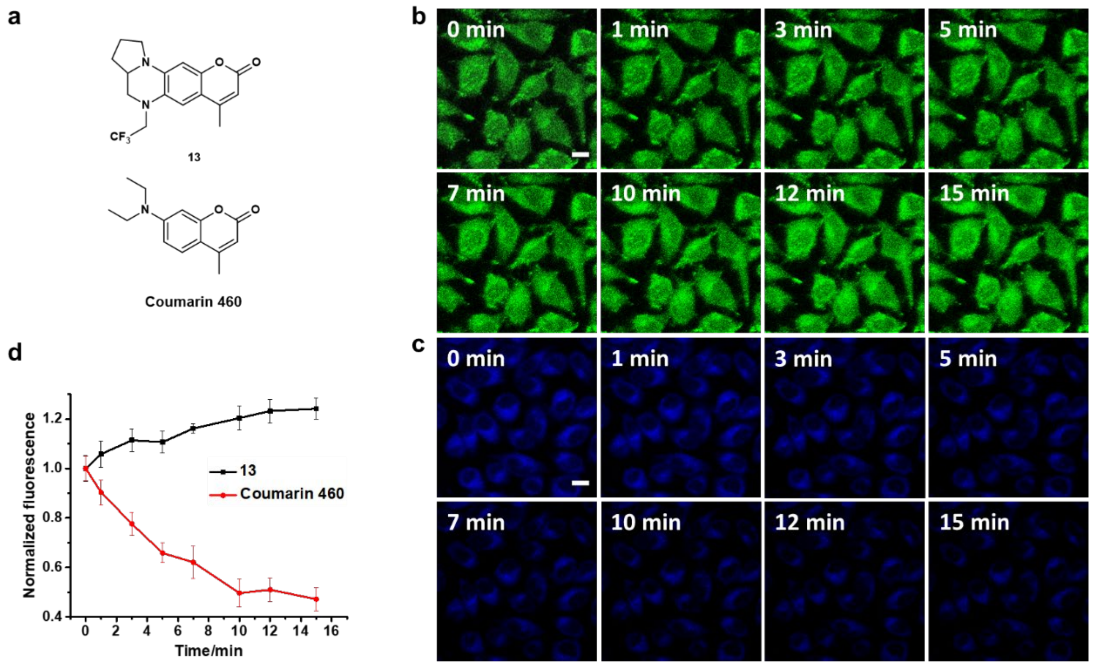

Supplementary Fig. 32 Comparison of **13** and Coumarin 460 (R-4) in live-cell imaging. (a) structures of **13** and Coumarin 460. (b-c) Confocal fluorescence images of live HeLa cells cultured with **13** (5.0 μM) (b) and Coumarin 460 (5.0 μM) (c) under continuous irradiation for 15 min. (d) Normalized fluorescence intensities of live HeLa cells in **b** and **c**. The fluorescence intensities were normalized to that of the cells incubated with **13** or Coumarin 460 for 0 min (F_0) before irradiation. HeLa cells in each group were examined in 3 independent experiments separately. Scale bar = 20 μm. For **13**, λ_{ex} = 405 nm, detection range, 500-550 nm. For Coumarin 460, λ_{ex} = 405 nm, detection range 425-475 nm. Error bars, ± s.e.m. from about 50 cells.

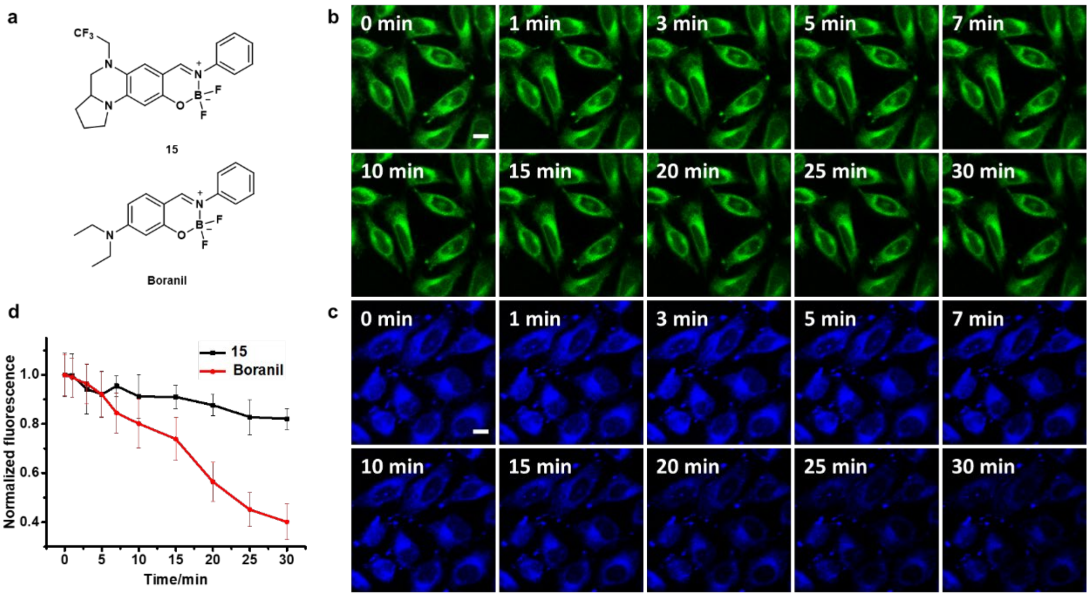

Supplementary Fig. 33 Comparison of **15** and Boranil (R-5) in live-cell imaging. (a) structures of **15** and Boranil. (b-c) Confocal fluorescence images of live HeLa cells cultured with **15** (5.0 μM) (b) and Boranil (5.0 μM) (c) under continuous irradiation for 30 min. (d) Normalized fluorescence intensities of live HeLa cells in **b** and **c**. The fluorescence intensities were normalized to that of the cells incubated with **15** or Boranil for 0 min (F_0) before irradiation. HeLa cells in each group were examined in 3 independent experiments separately. Scale bar = 20 μm. For **15**, λ_{ex} = 405 nm, detection range, 570-620 nm. For Boranil, λ_{ex} = 405 nm, detection range 425-475 nm. Error bars, ± s.e.m. from about 50 cells.

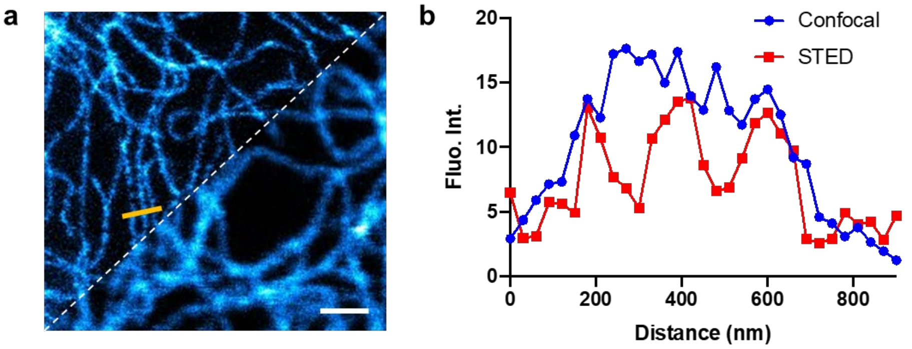

Supplementary Fig. 34 STED images of vimentin filaments in live U-2 OS cells that expressed vimentin-Halo fusion protein after incubation with **16-Halo** (50 nM) for 6 h. (b) Plot of line-scan intensity in a (yellow line) as a function of line length.Scale bar = 1 μm. λ_{ex} = 485 nm, detection range, 500–550 nm, STED laser 595 nm.

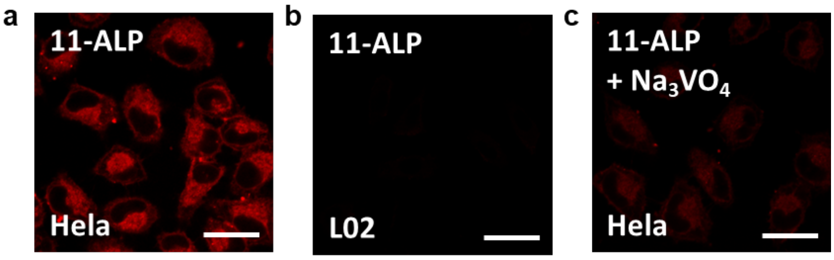

Supplementary Fig. 35 Utilization of **11-ALP** for sensing ALP in live-cell imaging. (a-b) Confocal images of living HeLa (a) or L02 (b) cells stained with **11-ALP** (5 µM) after 30 min of incubation. (c) Confocal fluorescence images of live HeLa cells incubated with **11-ALP** (5 µM) for 30 min with pre-incubation with 200 µM Na_3VO_4 for 1 h. Normalized fluorescence intensities of living HeLa cells in **a-c**. The fluorescence intensities were normalized to that of the L02 cells incubated with **11-ALP** (Figure 5i). Cells in each group were examined in 3 independent experiments separately. Scale bar = 20 µm. λ_{ex} = 561 nm, detection range, 585-675 nm.

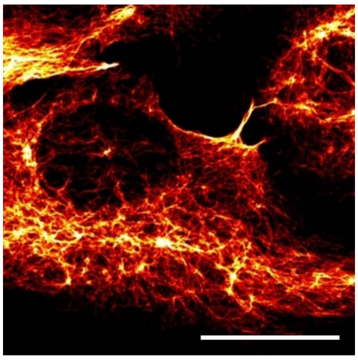

Supplementary Fig. 36 Two-photon images of vimentin filaments in live U-2 OS cells that expressed vimentin-Halo fusion protein after incubation with **YL578-Halo** (50 nM) for 6 h. Scale bar = 20 µm. λ_{ex} = 870 nm, detection range, 570–780 nm.

Supplementary Table 1 Photophysical properties of ***1-7, 11-15, 17-21*** in various solvents (CH_2Cl_2 (containing 0.1% TFA), MeCN (containing 0.1% TFA), EtOH, PBS buffer (25 mM)) at 25 °C.

1	λ_{Abs}/nm	λ_{Em}/nm	Stokes shift/nm	φ	ε/M^{-1} cm^{-1}	εφ($M^{-1}cm^{-1}$)
DCM	593	664	71	0.38	52800	20100
MeCN	591	672	81	0.34	55800	19000
EtOH	589	666	77	0.29	54600	15800
PBS	574	673	105	0.10	52200	5200

2	λ_{Abs}/nm	λ_{Em}/nm	Stokes shift/nm	φ	ε/M^{-1} cm^{-1}	εφ($M^{-1}cm^{-1}$)
DCM	583	644	61	0.42	66000	27700
MeCN	583	658	75	0.26	65000	16900
EtOH	579	665	86	0.17	58000	9860
PBS	571	661	90	0.09	66000	5940

3	λ_{Abs}/nm	λ_{Em}/nm	Stokes shift/nm	φ	ε/M^{-1} cm^{-1}	εφ($M^{-1}cm^{-1}$)
DCM	591	648	57	0.55	67800	37300
MeCN	588	652	64	0.45	74200	33400
EtOH	582	651	69	0.46	67800	31200
PBS	576	649	73	0.32	70000	22400

4	λ_{Abs}/nm	λ_{Em}/nm	Stokes shift/nm	φ	ε/M^{-1} cm^{-1}	εφ($M^{-1}cm^{-1}$)
DCM	581	642	61	0.70	70300	49200
MeCN	582	650	68	0.54	59600	32200
EtOH	575	649	74	0.52	59600	31000
PBS	575	637	62	0.59	70200	41400

5	λ_{Abs}/nm	λ_{Em}/nm	Stokes shift/nm	φ	ε/M^{-1} cm^{-1}	εφ($M^{-1}cm^{-1}$)
DCM	584	634	50	0.72	87100	62700
MeCN	583	639	56	0.61	68100	41500
EtOH	575	635	60	0.63	81400	51300
PBS	575	636	61	0.51	82500	42100

6	λ_{Abs}/nm	λ_{Em}/nm	Stokes shift/nm	φ	ε/M^{-1} cm^{-1}	εφ($M^{-1}cm^{-1}$)
DCM	582	625	43	0.86	101000	86900
MeCN	581	630	49	0.83	104000	86300
EtOH	579	628	49	0.85	91000	77400
PBS	578	634	56	0.74	89700	66400

7	λ_{Abs}/nm	λ_{Em}/nm	Stokes shift/nm	φ	ε/M^{-1} cm^{-1}	εφ($M^{-1}cm^{-1}$)
DCM	561	591	30	0.78	103000	80300
MeCN	562	597	35	0.55	96900	53300
EtOH	554	592	38	0.67	81700	57200
PBS	557	590	33	0.46	91500	42100

11	λ_{Abs}/nm	λ_{Em}/nm	Stokes shift/nm	φ	ε/M^{-1} cm^{-1}	εφ($M^{-1}cm^{-1}$)
DCM	530	590	60	0.76	44100	33500
MeCN	527	606	79	0.71	39000	27700
EtOH	530	606	66	0.68	43500	29600
PBS	548	612	64	0.62	51000	31600

17	λ_{Abs}/nm	λ_{Em}/nm	Stokes shift/nm	φ	ε/M^{-1} cm^{-1}	εφ($M^{-1}cm^{-1}$)
DCM	539	648	109	0.31	26300	8150
MeCN	534	667	133	0.06	24200	1450
EtOH	538	651	113	0.15	28600	4290
PBS	543	662	119	0.05	41600	2080

12	λ_{Abs}/nm	λ_{Em}/nm	Stokes shift/nm	φ	ε/M^{-1} cm^{-1}	εφ($M^{-1}cm^{-1}$)
DCM	585	620	35	0.83	102000	84700
MeCN	578	624	46	0.82	92200	75600
EtOH	580	623	43	0.76	103000	78300
PBS	577	623	46	0.71	83300	59100

18	λ_{Abs}/nm	λ_{Em}/nm	Stokes shift/nm	φ	ε/M^{-1} cm^{-1}	εφ($M^{-1}cm^{-1}$)
DCM	594	658	64	0.31	55200	17100
MeCN	587	667	80	0.27	52800	14300
EtOH	587	661	74	0.24	59200	14200
PBS	573	668	95	0.05	54300	2720

13	λ_{Abs}/nm	λ_{Em}/nm	Stokes shift/nm	φ	ε/M^{-1} cm^{-1}	εφ($M^{-1}cm^{-1}$)
DCM	390	467	77	0.91	33100	30100
MeCN	392	480	88	0.81	31900	25800
EtOH	400	488	88	0.91	33100	30100
PBS	406	513	117	0.55	32400	17800

19	λ_{Abs}/nm	λ_{Em}/nm	Stokes shift/nm	φ	ε/M^{-1} cm^{-1}	εφ($M^{-1}cm^{-1}$)
DCM	407	498	91	0.72	29000	20900
MeCN	407	511	104	0.52	32800	17100
EtOH	414	514	100	0.58	33000	19100
PBS	408	552	144	0.13	32600	4240

14	λ_{Abs}/nm	λ_{Em}/nm	Stokes shift/nm	φ	ε/M^{-1} cm^{-1}	εφ($M^{-1}cm^{-1}$)
DCM	447	520	73	0.84	22400	18800
MeCN	447	533	86	0.75	23200	17400
EtOH	452	538	86	0.88	24800	21800
PBS	464	556	92	0.67	26200	17600

20	λ_{Abs}/nm	λ_{Em}/nm	Stokes shift/nm	φ	ε/M^{-1} cm^{-1}	εφ($M^{-1}cm^{-1}$)
DCM	462	566	104	0.51	23000	11700
MeCN	461	586	125	0.34	22000	7480
EtOH	462	584	122	0.35	24600	8610
PBS	464	612	148	0.10	25800	2580

15	λ_{Abs}/nm	λ_{Em}/nm	Stokes shift/nm	φ	ε/M^{-1} cm^{-1}	εφ($M^{-1}cm^{-1}$)
DCM	439	548	109	0.78	29000	22600
MeCN	437	566	129	0.41	24700	10100
EtOH	438	563	125	0.43	24500	10500
PBS	439	575	136	0.40	25100	10300

21	λ_{Abs}/nm	λ_{Em}/nm	Stokes shift/nm	φ	ε/M^{-1} cm^{-1}	εφ($M^{-1}cm^{-1}$)
DCM	456	594	138	0.26	32300	8400
MeCN	453	619	166	0.075	30100	2260
EtOH	452	611	159	0.097	28600	2770
PBS	436	629	193	0.026	31900	830

Supplementary Table 2 Full width at half maximum (FWHM) of **1-6** (5 μM) and reference dyes (RhB and JF549, 5 μM) in PBS buffer (25 mM) at 25 °C.

Dye	$FWHM_{abs}$	$FWHM_{em}$
1	67 nm	98 nm
2	60 nm	90 nm
3	63 nm	73 nm
4	61 nm	74 nm
5	62 nm	71 nm
6	62 nm	64 nm
RhB	43 nm	51 nm
JF549	48 nm	47 nm

Synthesis Procedure

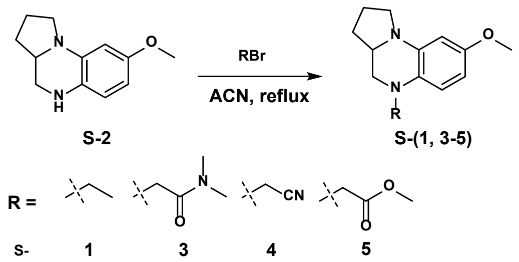

Supplementary Fig. 37 Synthesis of **S-(3-5).**

S-1 and **S-2** were synthesized based on the previous literature[5]. **S-2** (300.0 mg, 1.47 mmol, 1.0 eqiuv.) was dissolved in 20 mL MeCN and then the mixture was stirred strongly. K_2CO_3 (608.8 mg, 4.41 mmol, 3.0 eqiuv.) and RBr (1.5 eqiuv.) were next added and the mixture was refluxed for 2 h. After the reaction completing monitored by TLC analysis, the mixture was cooled to r.t. and evaporated. The residue was purified by column chromatography on silica gel using an EA/PE mixture as the eluent.

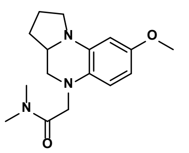

S-3. 361.3 mg. Yield: 85%. Light-yellow oily liquid. ^{1}H NMR (400 MHz, $CDCl_3$) δ 6.36 (d, J = 7.6 Hz, 1H), 6.03 (s, 2H), 4.17 – 3.80 (m, 2H), 3.70 (s, 4H), 3.50 – 3.10 (m, 2H), 3.02 (s, 4H), 2.93 (s, 4H), 2.06 – 1.98 (m, 2H), 1.96 – 1.87 (m, 1H), 1.40 (p, J = 9.7, 9.3 Hz, 1H). ^{13}C NMR (100 MHz, $CDCl_3$) δ 169.43, 153.44, 136.49, 127.49, 111.01, 98.95, 97.93, 77.00, 56.29, 55.11, 54.12, 52.23, 47.39, 36.40, 35.31, 29.75, 23.24.HRMS (EI): m/z calc. for $C_{16}H_{23}N_3O_2$ [M] 289.1790; found 289.1794.

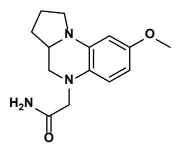

S-4. 353.1 mg. Yield: 92%. Colorless oi ly liquid. ^{1}H NMR (400 MHz, $CDCl_3$) δ 6.43 (d, *J* = 8.2 Hz, 1H), 6.09 (s, 2H), 3.99 (s, 2H), 3.77 (s, 3H), 3.35 (s, 3H), 3.10 (s, 4H), 3.00 (s, 4H), 2.11 – 2.05 (m, 2H), 2.03 – 1.94 (m, 1H), 1.47 (d, *J* = 7.6 Hz, 1H). ^{13}C NMR (100 MHz, $CDCl_3$) δ 155.46, 137.71, 125.07, 115.65, 113.09, 99.45, 98.63, 56.45, 55.61, 53.04, 47.89, 41.11, 30.42, 23.82. MALDI-TOF/MS, m/z: calc 243.14, found 243.12.

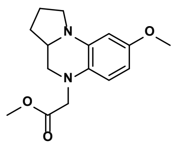

S-5. 385.6 mg. Yield: 95%. Colorless oily liquid. ^{1}H NMR (400 MHz, CD Cl_3) δ 6.32 (d, *J* = 8.5 Hz, 1H), 6.12 (s, 1H), 6.07 (s, 1H), 4.15 (d, *J* = 9.3 Hz, 1H), 3.85 – 3.77 (m, 1H), 3.74 (s, 3H), 3.72 (s, 3H), 3.65 – 3.49 (m, 1H), 3.34 (s, 2H), 3.26 (s, 1H), 3.05 (s, 1H), 2.11 – 2.01 (m, 2H), 2.01 – 1.91 (m, 1H), 1.44 (t, *J* = 10.1 Hz, 1H). ^{13}C NMR (100 MHz, $CDCl_3$) δ 171.69, 153.72, 136.44, 127.29, 110.85, 99.49, 98.54, 56.41, 55.48, 53.75, 53.43, 51.76, 47.54, 29.98, 23.39. ESI/MS, m/z: calc 276.15, found 276.07.

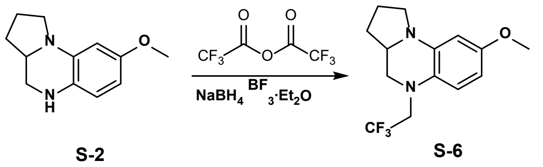

Supplementary Fig. 38 Synthesis of **S-6.**

S-2 (1.0 g, 4.9 mmol, 1.0 eqiuv.) was dissolved in 20 mL THF and the mixture was stirred strongly. Trifluoroacetic anhydride (2.1 g, 1.4 mL, 9.8 mmol, 2.0 equiv.) was added dropwise to the above solution and the mixture was continuously stirred for 10 min at r.t.. $NaBH_4$ (0.9 g, 24.5 mmol, 5.0 equiv.) and BF_3·Et_2O (47 %, 6.5 mL, 5.0 equiv.) were added slowly and the mixture was refluxed for 1-2 h. After the reaction completing monitored by TLC analysis, the mixture was cooled to r.t.. Ice-water (10 mL) was added dropwise to quench the reaction (note: the reaction was violently exothermic). The mixture was extracted with ethyl acetate (EA, 30.0 mL) three times. The organic layers were combined, dried with Na_2SO_4, and evaporated. The residue was purified by column chromatography on silica gel using an EA/PE mixture as the eluent.

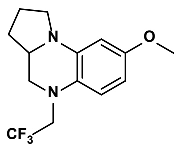

S-6. 981.4 mg. Yield: 70%. Light-yellow oily liquid. ^{1}H NMR (400 MHz, $CDCl_3$) δ 6.66 (d, J = 8.6 Hz, 1H), 6.20 (d, J = 8.6 Hz, 1H), 6.13 – 6.07 (m, 1H), 3.76 (d, J = 7.5 Hz, 4H), 3.64 (m, J = 8.9 Hz, 1H), 3.45 (d, J = 8.0 Hz, 1H), 3.39 (d, J = 8.4 Hz, 1H), 3.30 (t, J = 8.0 Hz, 2H), 2.86 (t, J = 10.2 Hz, 1H), 2.09 (m, 2H), 2.02 – 1.94 (m, 1H), 1.48 – 1.41 (m, 1H). ^{13}C NMR (100 MHz, $CDCl_3$) δ 154.38, 136.74, 126.93, 124.23, 114.10, 100.08, 98.57, 55.51, 54.56, 54.02, 47.29, 29.81, 23.19. ESI/MS, m/z: calc 286.13, found 286.13.

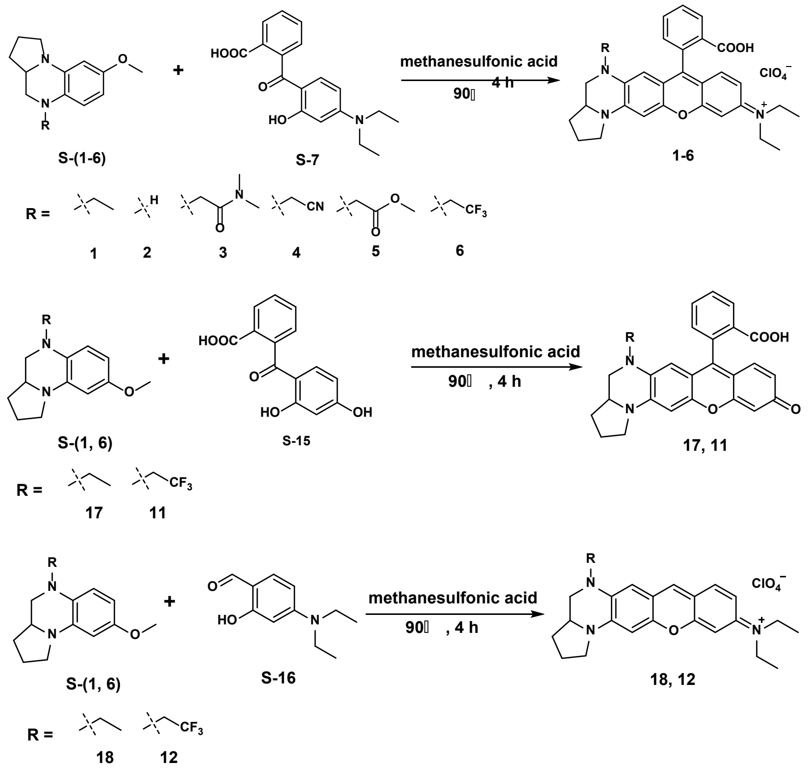

Supplementary Fig. 39 Synthesis of **1-6, 11, 12, 17, 18.**

2-(4-(diethylamino)-2-hydroxybenzoyl)benzoic acid/ 2-(2,4-dihydroxybenzoyl)benzoic acid/2-(8-hydroxy-2,3,6,7-tetrahydro-1H,5H-pyrido[3,2,1-ij]quinoline-9-carbonyl)benzoic acid (1.1 equiv.) and **S-(1-6)** (300.0 mg, 1.0 equiv.) were dissolved in methanesulfonic acid (3.0 mL). The mixture was stirred and heated at 90 °C for 3-6 h. After the reaction completing monitored by TLC analysis, the mixture was poured into ice water, and then perchloric acid (0.5 mL) was added. The resulting precipitate was filtered off and washed with water (200 mL). After the sample dried, purification by silica gel chromatography using a CH_2Cl_2/EtOH mixture as the eluent gave compound **1-6** (It was worth noting that the reaction of **S-4** with 2-(4-diethylamino-2-hydroxybenzoyl)benzoic acid in methanesulfonic acid would produce the corresponding acetamido substituted product **4**).

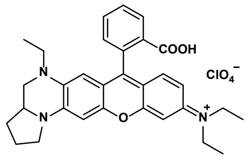

1: 499 .2 mg. Yield: 65%. Atropurpureus solid. ^{1}H NMR (400 MHz, CD_3OD containing 20% $CDCl_3$) δ 8.22 (s, 1H), 7.71 (s, 2H), 7.29 (s, 1H), 7.14 (t, J = 8.2 Hz, 1H), 6.96 – 6.83 (m, 2H), 6.68 (s, 1H), 6.05 (d, J = 10.1 Hz, 1H), 3.85 – 3.70 (m, 2H), 3.63 – 3.54 (d, J = 7.6 Hz, 6H), 3.33 (s, 2H), 2.85 (t, J = 9.9 Hz, 1H), 2.30 – 2.04 (m, 3H), 1.64 – 1.52 (m, 1H), 1.25 (s, 9H). ^{13}C NMR (100 MHz, CD_3OD containing 20% $CDCl_3$) δ 156.56, 156.27, 154.63, 153.34, 146.27, 134.44, 134.41, 130.77, 130.59, 130.09, 129.70, 129.67, 129.31, 115.41, 113.36, 113.03, 105.27, 105.10, 95.45, 94.49, 58.31, 45.13, 43.81, 43.73, 29.55, 22.81, 11.77. HRMS (ESI): m/z calc. for $C_{31}H_{34}N_3O_3$ [M] 496.2595; found 496.2606.

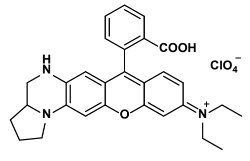

2: 665.9 mg. Yield: 80%. Atropurp ureus solid. ^{1}H NMR (400 MHz, CD_3OD containing 20% $CDCl_3$) δ 8.28 (s, 1H), 7.76 (s, 2H), 7.34 (s, 1H), 7.24 (d, J = 8.8 Hz, 1H), 6.98 (d, J = 9.1 Hz, 1H), 6.92 (s, 1H), 6.74 (s, 1H), 6.35 (s, 1H), 3.87 – 3.77 (m, 2H), 3.67 (q, J = 6.5 Hz, 6H), 2.88 (q, J = 10.2 Hz, 1H), 2.38 – 2.28 (m, 2H), 2.26 – 2.16 (m, 1H), 1.73 – 1.60 (m, 1H), 1.36 (t, J = 6.8 Hz, 6H). ^{13}C NMR (100 MHz, CD_3OD containing 20% $CDCl_3$) δ 156.23, 156.18, 154.65, 153.31, 146.96, 133.71, 131.29, 131.00, 130.73, 129.99, 129.92, 129.79, 129.69, 129.55, 115.39, 113.36, 113.09, 103.21, 95.55, 94.64, 57.95, 49.58, 45.43, 45.16, 29.71, 22.65, 11.80, 8.76, 8.52. HRMS (ESI): m/z calc. for $C_{29}H_{30}N_3O_3$ [M] 468.2282; found 468.2295.

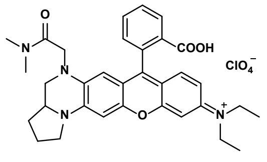

3: 473.0 mg. Yield: 70%. Atropurpureus solid. ^{1}H NMR (400 MHz, CD_3OD containing 20% $CDCl_3$) δ 8.47 (t, J = 7.3 Hz, 1H), 7.98 – 7.87 (m, 2H), 7.43 (t, J = 7.1 Hz, 1H), 7.25 (t, J = 9.0 Hz, 1H), 7.02 (d, J = 9.2 Hz, 1H), 6.98 (s, 1H), 6.81 (s, 1H), 5.70 (d, J = 7.6 Hz, 1H), 4.26 (t, J = 9.9 Hz, 1H), 4.05 – 3.99 (m, 1H), 3.97 – 3.87 (m, 2H), 3.72 (d, J = 7.2 Hz, 6H), 3.53 – 3.46 (m, 1H), 2.98 – 2.87 (m, 6H), 2.47 – 2.35 (m, 2H), 2.35 – 2.20 (m, 1H), 1.79 – 1.70 (m, 1H), 1.42 (t, J = 6.9 Hz, 6H). ^{13}C NMR (100 MHz, CD_3OD containing 20% $CDCl_3$) δ 169.23, 167.80, 167.23, 157.28, 156.19, 155.62, 154.43, 147.48, 135.50, 134.89, 133.66, 133.20, 132.11, 131.68, 131.23, 130.65, 115.95, 114.29, 104.10, 96.68, 95.72, 58.97, 53.27, 52.81, 46.26, 36.74, 36.04, 30.57, 23.67, 12.90. HRMS (ESI): m/z calc. for $C_{33}H_{37}N_4O_4$ [M] 553.2809; found 553.2814.

4: 551.4 mg. Yield: 77%. Atropurpureus solid. ^{1}H NMR (400 MHz, CD_3OD

containing 20% $CDCl_3$) δ 8.25 (s, 1H), 7.76 (s, 1H), 7.73 (d, *J* = 5.7 Hz, 1H), 7.51 (t, *J* = 9.6 Hz, 1H), 7.33 (d, *J* = 5.7 Hz, 1H), 7.08 (d, *J* = 9.2 Hz, 1H), 7.00 (s, 1H), 6.82 (s, 1H), 6.35 (s, 1H), 4.29 – 4.10 (m, 1H), 4.08-3.85 (m, 3H), 3.78 (q, *J* = 6.8 Hz, 6H), 3.51 (s, 1H), 2.53 – 2.24 (m, 3H), 1.85 – 1.70 (m, 1H), 1.48 (t, *J* = 7.1 Hz, 6H). ^{13}C NMR (100 MHz, CD_3OD containing 20% $CDCl_3$) δ 159.95, 159.58, 157.57, 155.66, 155.58, 154.56, 147.24, 134.68, 134.47, 131.89, 130.21, 130.17, 116.11, 116.00, 114.62, 114.51, 114.01, 106.17, 105.89, 96.42, 95.53, 58.88, 58.67, 53.63, 53.14, 46.21, 30.59, 23.76, 12.93. HRMS (ESI): m/z calc. for $C_{31}H_{33}N_4O_4$ [M] 525.2496; found 525.2509.

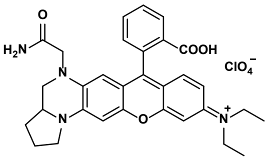

5: 416.1 mg. Yield: 60%. Atropurpureus solid. ^{1}H NMR (400 MHz, CD_3OD) δ 8.31 (d, *J* = 6.7 Hz, 1H), 7.81 (m, 2H), 7.34 (d, *J* = 6.8 Hz, 1H), 7.11 (d, *J* = 8.5 Hz, 1H), 6.98 (d, *J* = 9.0 Hz, 1H), 6.91 (s, 1H), 6.72 (s, 1H), 5.76 (s, 1H), 3.88 (s, 2H), 3.86 – 3.79 (m, 1H), 3.74 (t, *J* = 10.0 Hz, 1H), 3.61 (m, 9H), 3.20 (t, *J* = 10.4 Hz, 1H), 2.30 – 2.19 (m, 2H), 2.17 – 2.05 (m, 1H), 1.59 (m, 1H), 1.30 – 1.25 (m, 6H). ^{13}C NMR (100 MHz, CD_3OD) δ 171.08, 157.63, 157.52, 155.95, 154.96, 147.69, 135.62, 134.74, 133.26, 132.10, 131.05, 130.97, 130.81, 116.01, 114.53, 114.39, 104.75, 96.65, 95.84, 71.18, 59.18, 53.55, 52.77, 52.46, 48.80, 46.22, 30.60, 23.70, 12.59. HRMS (ESI): m/z calc. for $C_{32}H_{34}N_3O_5$ [M] 540.2493; found 540.2496.

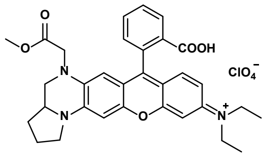

6: 475.7 mg. Yield: 70%. Atropurpureus solid. ^{1}H NMR (400 MHz, CD_3OD containing 20% $CDCl_3$) δ 8.43 – 8.36 (t, *J* = 6.8 H z, 1H), 7.85 (m, 2H), 7.38 (t, *J* = 8.4 Hz, 1H), 7.21 (t, *J* = 9.4 Hz, 1H), 7.02 – 6.91 (m, 2H), 6.80 (s, 1H), 6.27 (d, *J* = 7.5 Hz, 1H), 3.97 – 3.86 (m, 2H), 3.85 – 3.77 (m, 3H), 3.68 (q, *J* = 6.8 Hz, 5H), 3.17 (t, *J* = 9.8 Hz, 1H), 2.37 (m, 2H), 2.29 – 2.14 (m, 1H), 1.69 (m, 1H), 1.37 (t, *J* = 7.0 Hz, 6H). ^{13}C NMR (100 MHz, CD_3OD containing 20% $CDCl_3$) δ 167.00, 156.73, 156.59, 154.47, 154.03, 146.26, 134.12, 132.72, 132.50, 131.27, 130.98, 130.12, 130.03, 129.96, 114.59, 113.64, 113.41, 104.95, 95.75, 95.29, 57.45, 57.42, 53.39, 51.81, 45.40, 29.55, 22.66, 11.94. HRMS (ESI): m/z calc. for $C_{31}H_{31}F_3N_3O_3$ [M] 550.2312; found 550.2329.

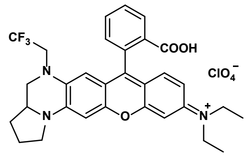

The crystal data is displayed behind the NMR spectrum.

11: 378.3 mg. Yield: 73%. Crimson solid. ^{1}H NMR (400 MHz, CD_3OD c ontaining 20% $CDCl_3$) δ 8.35 (s, 1H), 7.83 (m, 2H), 7.40 (t, *J* = 7.8 Hz, 1H), 7.20 (t, *J* = 9.0 Hz, 1H), 7.11 (s, 1H), 6.96 (d, *J* = 8.7 Hz, 1H), 6.89 (s, 1H), 6.32 – 6.23 (m, 1H), 3.98 (m, 1H), 3.84 (m, 3H), 3.75 – 3.68 (m, 1H), 3.67 – 3.56 (m, 1H), 3.18 (t, *J* = 9.9 Hz, 1H), 2.29 (dd, *J* = 9.6, 4.7 Hz, 2H), 2.14 (m, 1H), 1.67 (m, 1H).^{13}C NMR (100 MHz, MeOD containing 20% $CDCl_3$) δ 166.15, 157.70, 157.62, 157.09, 156.28, 156.21, 149.47, 134.96, 133.57, 133.46, 132.22, 131.05, 130.96, 130.85, 130.78, 117.84, 117.72, 116.33, 104.82, 102.78, 96.31, 59.01, 58.00, 49.92, 30.30, 23.38, 18.09. HRMS (ESI): m/z calc. for $C_{27}H_{21}F_3N_2O_4$ $[M+H]^+$ 495.1526; found 495.1526.

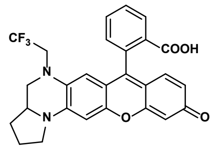

12: 393.2 mg. Yield: 71%. Atropurpureus solid. ^{1}H NMR (4 00 MHz, CD_3OD containing 20% $CDCl_3$) δ 8.50 (s, 1H), 7.84 (d, *J* = 8.4 Hz, 1H), 7.20 (d, *J* = 8.7 Hz, 1H), 7.13 (s, 1H), 6.96 (s, 1H), 6.80 (s, 1H), 3.93 (d, *J* = 11.0 Hz, 2H), 3.75 (d, *J* = 6.9 Hz, 6H),

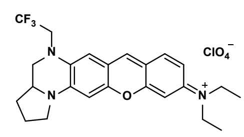

3.29 (s, 2H), 2.45 – 2.34 (m, 2H), 2.25 (d, *J* = 6.8 Hz, 1H), 1.80 – 1.68 (m, 1H), 1.42 (d, *J* = 7.0 Hz, 6H). ^{13}C NMR (100 MHz, CD_3OD containing 20% $CDCl_3$) δ 156.93, 154.74, 154.52, 146.87, 142.61, 133.03, 131.89, 126.88, 115.67, 114.07, 113.77, 106.62, 95.63, 95.23, 57.61, 54.86, 51.72, 45.40, 44.16, 29.51, 22.57, 11.77. MALDI-TOF/MS, m/z: calc 430.21, found 430.16.

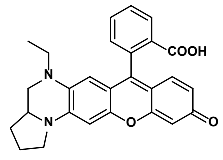

17: 438.2 mg. Yield: 77%. Atropurpureus solid. ^{1}H NMR (40 0 MHz, CD_3OD containing 20% $CDCl_3$) δ 8.37 (d, *J* = 7.6 Hz, 1H), 7.84 (m, 2H), 7.40 (t, *J* = 8.8 Hz, 1H), 7.21 (m, 1H), 7.13 (s, 1H), 6.97 (d, *J* = 9.0 Hz, 1H), 6.84 (s, 1H), 6.04 (d, *J* = 4.4 Hz, 1H), 3.89 (m, 2H), 3.77 – 3.63 (m, 2H), 3.32 – 3.24 (m, 1H), 3.24 – 3.11 (m, 1H), 3.04 (m, 1H), 2.34 (s, 2H), 2.19 (s, 1H), 1.70 (m, 1H), 1.09 – 0.95 (m, 3H). ^{13}C NMR (100 MHz, CD_3OD containing 20% $CDCl_3$) δ 168.09, 165.47, 157.42, 155.87, 155.47, 150.22, 135.96, 135.70, 133.90, 133.74, 132.42, 131.39, 131.21, 130.63, 118.84, 117.71, 116.59, 103.05, 102.72, 96.26, 59.57, 50.50, 50.06, 46.76, 30.77, 23.67, 9.57. MALDI-TOF/MS, m/z: calc 440.17, found 441.13. $[M+H]^-$

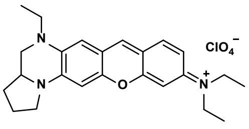

18: 478.1 mg. Yield: 78%. Atropurpureus solid. ^{1}H N MR (400 MHz, CD_3OD containing 20% $CDCl_3$) δ 8.40 (s, 1H), 7.79 (d, *J* = 9.1 Hz, 1H), 7.15 (d, *J* = 8.9 Hz, 1H), 6.91 (s, 1H), 6.84 (s, 1H), 6.70 (s, 1H), 4.01 – 3.90 (m, 1H), 3.86 – 3.79 (m, 1H), 3.73 (q, *J* = 6.8 Hz, 6H), 3.50 (m, 2H), 3.02 (m, 1H), 2.46 – 2.32 (m, 2H), 2.29 – 2.18 (m, 1H), 1.72 (m, 1H), 1.44 – 1.34 (m, 9H). ^{13}C NMR (100 MHz, CD_3OD containing 20% $CDCl_3$) δ 157.51, 155.96, 154.95, 148.72, 142.32, 135.18, 132.60, 117.77, 114.90, 114.53, 105.71, 96.75, 95.89, 59.21, 50.26, 46.62, 46.44, 46.26, 30.85, 23.75, 12.98, 10.15. MALDI-TOF/MS, m/z: calc 376.24, found 376.21.

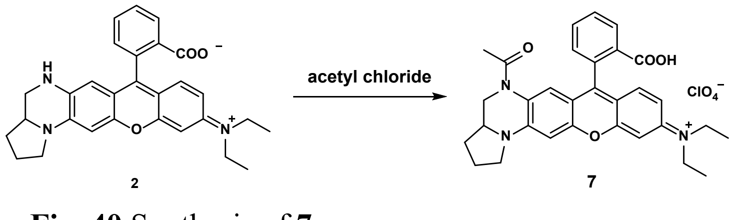

Supplementary Fig. 40 Synthesis of **7**.

2 (100.0 mg, 176.7 μmol, 1.0 equiv.) and DIPEA (87.0 μL, 530.1 μmol, 3.0 equiv.) was dissolved into CH_2Cl_2 (10.0 mL). Acetyl chloride (25.0 μL, 353.4 μmol, 2.0 equiv.) was added dropwise and the mixture was stirred at r.t. for 1 h. water (10.0 mL) was added carefully to the above mixture for quenching the reaction. The mixture was extracted with CH_2Cl_2 (20.0 mL) three times. The organic phases were washed with H_2O containing 0.5 mL $HClO_4$ for three times, dried, concentrated. And the crude was purified by column chromatography on silica gel chromatography using a CH_2Cl_2/EtOH mixture as the eluent.

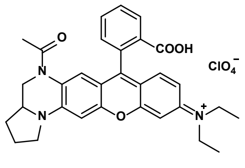

7: 67.7 mg. Yield 63%. Crimson solid. ^{1}H NMR (400 M Hz, CD_3OD) δ 8.25 (d, *J* = 6.7 Hz, 1H), 7.74 (m, 2H), 7.33 (d, *J* = 6.7 Hz, 1H), 7.23 (d, *J* = 9.2 Hz, 1H), 7.17 (d, *J* = 9.5 Hz, 1H), 6.95 (d, *J* = 9.8 Hz, 1H), 6.90 (s, 1H), 6.81 (s, 1H), 3.81 – 3.70 (m, 2H), 3.70 – 3.55 (m, 7H), 2.34 – 2.24 (m, 2H), 2.11 (m, 4H), 1.64 – 1.56 (m, 1H), 1.32 –

1.28 (m, 6H). Analytical HPLC: >95% purity (4.6 mm × 250 mm 5 μm C18 column; 100 μL injection: 5-95% CH_3CN/H_2O, linear gradient, with constant 0.1% v/v TFA additive; 10 min run; 1 mL/min flow; UV detection at 560 nm). ESI-MS m/z calcd for $C_{31}H_{36}N_3O_4$ [M], 510.24; Found, 510.30.

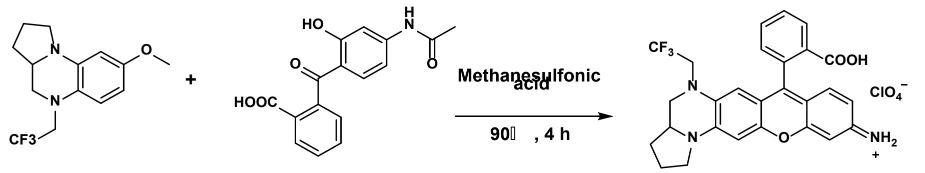

S-6 S-29 8

Supplementary Fig. 41 Synthesis of **8**.

S-6 (300.0 mg, 1.05 mmol, 1.0 equiv.) and S-29 (345.3 mg, 1.2 mmol, 1.1 equiv) were dissolved in methanesulfonic acid (3.0 mL). The mixture was stirred and heated at 90 °C for 3-6 h. After the reaction completing monitored by TLC analysis, the mixture was poured into ice water, and then perchloric acid (0.5 mL) was added. The resulting precipitate was filtered off and washed with water (200 mL). After the sample dried, purification by silica gel chromatography using a CH_2Cl_2/EtOH mixture as the eluent.

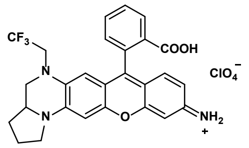

8: 155.0 mg. Yield: 24.9%. Atropurpureus solid. ^{1}H NMR (400 MHz, DMSO-d^6) δ 8.17 (s, 1H), 7.77 (m, 2H), 7.56 (s, 2H), 7.34 (d, J = 5.1 Hz, 1H), 6.93 (t, J = 9.0 Hz, 1H), 6.83 (s, 1H), 6.77 (d, J = 11.6 Hz, 2H), 6.06 (s, 1H), 4.03 (m, 1H), 3.87 (m, 1H), 3.78 – 3.64 (m, 3H), 3.57 – 3.49 (m, 1H), 2.98 (m, 1H), 2.18 – 2.05 (m, 2H), 2.01 – 1.90 (m, 1H), 1.55 – 1.42 (m, 1H). ^{13}C NMR (100 MHz, DMSO-d^6) δ 166.31, 157.28, 156.00, 153.44, 145.61, 132.59, 132.48, 132.36, 130.95, 130.70, 130.27, 130.00, 126.86, 124.03, 116.27, 113.50, 112.84, 104.43, 96.78, 95.34, 57.16, 51.18, 48.43, 39.51, 29.12, 28.99, 22.38. MALDI-TOF/MS, m/z calc. for $C_{27}H_{23}F_3N_3O_3$ [M] calc 494.17, found 494.11.

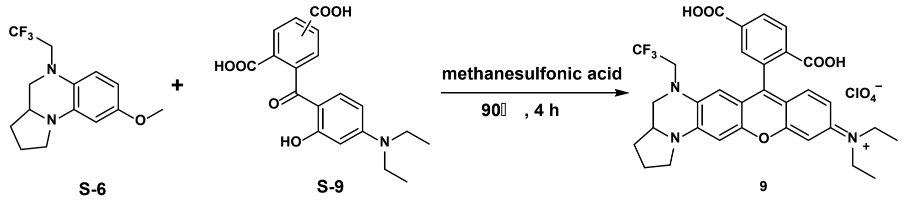

S-6 S-9 9

Supplementary Fig. 42 Synthesis of **9.**

S-6 (150.0 mg, 524.4 μmol, 1.0 equiv.) and 2 (225.0 mg, 629.4 μmol, 1.2 equiv.) were dissolved in methanesulfonic acid (4.0 mL) and then stirred at 90 °C for 4 h. The mixture was cooled to r.t. and poured into ice-water (100.0 mL), and then perchloric acid (0.5 mL) was added. The resulting precipitate was filtered off and washed with ice water (100 mL). After the sample dried, purification by silica gel chromatography using a CH_2Cl_2/EtOH mixture as the eluent.

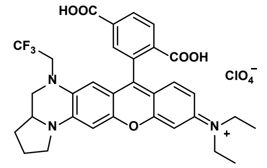

9: 127.0 mg. Yield 35%. Atropurpureus solid. ^{1}H N MR (400 MHz, CD_3OD) δ 8.38 (m, 2H), 7.95 (s, 1H), 7.15 – 7.04 (m, 1H), 6.99 (s, 1H), 6.93 (s, 1H), 6.76 (d, J = 7.1 Hz, 1H), 6.19 (s, 1H),

3.92 (m, 1H), 3.74 (d, J = 10.0 Hz, 4H), 3.66 – 3.57 (m, 5H), 3.04 (t, J = 9.8 Hz, 1H), 2.29 – 2.20 (m, 2H), 2.12 (s, 1H), 1.64 – 1.54 (m, 1H), 1.27 (s, 6H). ^{13}C NMR (100 MHz, CD_3OD) δ 167.53, 167.17, 157.67, 155.87, 155.62, 155.19, 147.51, 136.01, 135.74, 135.60, 135.47, 134.09, 132.53, 132.27, 131.91, 130.73, 127.90, 125.09, 115.54, 114.92, 114.28, 105.55, 96.81, 96.39, 58.66, 52.95, 46.36, 30.49, 27.75, 23.62, 23.59, 12.64. HRMS (ESI): m/z calc. for $C_{32}H_{31}F_3N_3O_5$ [M] 594.2210; found 594.2223.

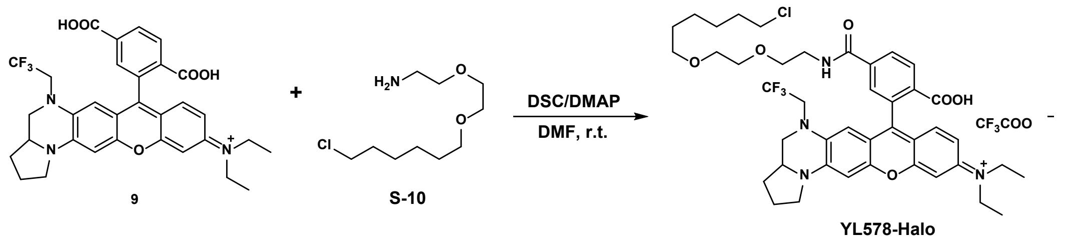

Supplementary Fig. 43 Synthesis of **YL578-Halo.**

9 (50.0 mg, 72.3 μmol, 1.0 equiv.), N,N'-Disuccinimidyl carbonate (DSC, 28.0 mg, 108.4 μmol, 1.5 equiv.), DMAP (0.9 mg, 7.2 μmol, 0.1 equiv.) and DIPEA (35.8 μL, 216.9 μmol, 3.0 equiv.) were dissolved in DMF (3.0 mL) and stirred at 60 °C for 4 h. The solvent was removed under reduced pressure and the residue was dissolved in THF. 3 (18.8 mg, 79.5 μmol, 1.1 equiv.) and DIPEA (35.8 μL, 216.9 μmol, 3.0 equiv.) were added. The mixture was stirred at r.t. for 5 h. The solvent was removed and the residue was purified by Prep-HPLC.

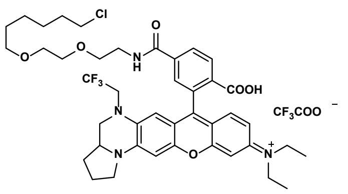

YL578-Halo. 35.0 mg. Yield: 54%. Atropurpureus solid. ^{1}H NMR (400 MHz, CD_3OD) δ 8.26 (s, J = 7.2 Hz, 1H), 8.15 (d, J = 7.2 Hz, 1H), 7.79 (s, 1H), 7.18 (d, J = 9.2 Hz, 1H), 6.98 (d, J = 8.2 Hz, 1H), 6.93 (s, 1H), 6.78 (s, 1H), 6.32 (d, J = 9.0 Hz, 1H), 3.90 (m, 1H), 3.85 – 3.69 (m, 5H), 3.64 (m, 11H), 3.59 – 3.48 (m, 6H), 3.42 (t, J = 6.3 Hz, 2H), 3.21 (q, J = 7.3 Hz, 1H), 3.11 – 3.02 (m, 1H), 2.30 – 2.20 (m, 2H), 2.19 – 2.06 (m, 1H), 1.75 – 1.67 (m, 2H), 1.66 – 1.55 (m, 1H), 1.48 (m, 2H), 1.27 (t, J = 5.9 Hz, 6H). ^{13}C NMR (100 MHz, CD_3OD) δ 166.92, 156.60, 156.30, 154.53, 154.00, 146.33, 136.42, 136.21, 132.92, 132.66, 130.49, 130.14, 128.81, 128.22, 114.46, 113.52, 113.26, 105.06, 95.54, 95.08, 70.72, 69.73, 69.02, 57.53, 54.43, 52.19, 45.15, 44.38, 42.41, 39.74, 32.33, 29.38, 29.04, 26.29, 25.03, 22.48, 11.76, 11.49. HRMS (ESI): m/z calc. for $C_{42}H_{51}ClF_3N_4O_6$ [M] 799.3444; found 799.3452.

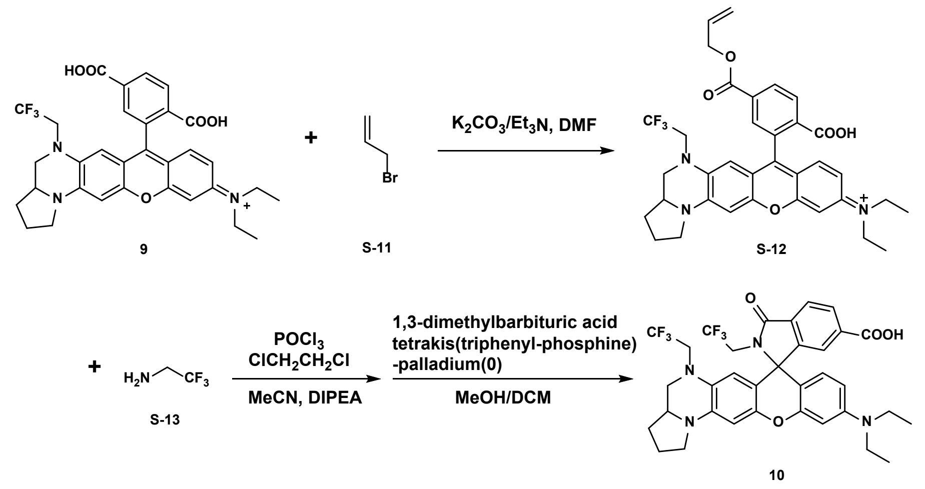

Supplementary Fig. 44 Synthesis of **10.**

9 (100.0 mg, 144.5 µmol, 1.0 equiv.) was dissolved in DMF (3.0 mL) and K_2CO_3 (39.9 mg, 289.0 µmol, 2.0 equiv.) and Et_3N (40.0 µL, 289.0 µmol, 2.0 equiv.) were added subsequently. The reaction was cooled down in an ice bath and allyl bromide (S-11, 26.2 mg, 216.8 µmol, 1.5 equiv.) was slowly added. Then the mixture was allowed to warm up to r.t. and stirred for 2 h. The reaction was diluted with water and extracted with CH_2Cl_2 (20.0 mL) three times. The combined organic phase was washed with brine, dried with $MgSO_4$, filtered, and concentrated in vacuo. The mixture was purified by fast flash chromatography on silica gel using a CH_2Cl_2/EtOH mixture as the eluent, obtaining the crude product S-12. The crude product was dissolved in CH_2ClCH_2Cl (4.0 mL). $POCl_3$ (67.3 µL, 722.5 µmol, 5.0 equiv.) was added and the reaction was refluxed for 3 h. The solution was removed by rotary evaporator and the crude acyl chloride residue was dissolved in dry MeCN (3.0 mL). DIPEA (119.4 µL, 722.5 µmol, 5.0 equiv.) and 2,2,2-Trifluoroethylamine (S-13, 28.6 mg, 289.0 µmol, 2.0 equiv.) were added quickly and the reaction was stirred at 90 ºC for 2 h. The solution was removed by rotary evaporator and the residue was mixed with 1,3-dimethylbarbituric acid (67.7 µg, 433.5 µmol, 3.0 equiv.), and tetrakis(triphenyl-phosphine)palladium(0) (83.5 mg, 72.3 µmol, 0.5 equiv.) in MeOH/ CH_2Cl_2 (5/1) and stirred at r.t for 1 h. The solvent was removed and the residue was purified by silica gel flash chromatography using a CH_2Cl_2/EtOH mixture containing 0.5% triethylamine as the eluent.

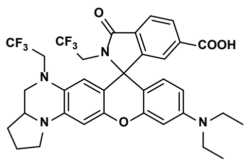

10. 34.6 mg. Yield:31%. Purple solid. ^1H NMR (400 MHz, $CDCl_3$) δ 8.12 (d, J = 6.2 Hz, 1H), 7.87 (d, J = 6.5 Hz, 1H), 7.81 – 7.56 (m, 2H), 6.37 – 6.13 (m, 3H), 5.74 (m, 1H), 3.75 – 3.56 (m, 1H), 3.46 – 3.36 (m, 2H), 3.34 – 3.25 (m, 5H), 3.03 – 2.97 (m, 4H), 2.82 (m, 1H), 2.12 – 1.98 (m, 2H), 2.00 – 1.87 (m, 1H), 1.39 (m, 1H), 1.23 – 1.19 (m, 6H). Analytical HPLC: >95% purity (4.6 mm × 250 mm 5 µm C18 column; 100 µL injection; 5-95% CH_3CN/H_2O, linear gradient, with constant 0.1% v/v TFA additive; 10 min run; 1 mL/min flow; UV detection at 580 nm). HRMS (ESI): m/z calc. for $C_{34}H_{32}F_6N_4O_4$ $[M+H]^+$ 674.2401; found 674.2405.

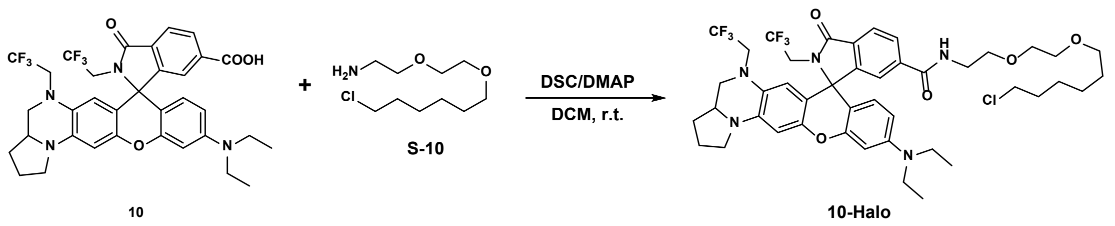

Supplementary Fig. 45 Synthesis of **10-Halo.**

Please refer to the synthesis of **9-Halo**. After purification, the solution was evaporated and dissolved in CH_2Cl_2 (10.0 mL). The mixture was washed with 0.5mM NaOH aqueous solution for three times. The organic phase was dried over anhydrous $MgSO_4$, filtered, evaporated, to obtain final product.

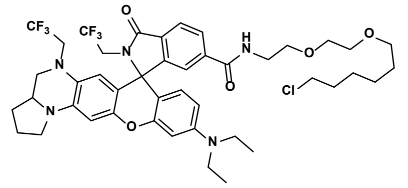

10-Halo: 7.6 mg. Yield 67%. Purple solid. ^{1}H NMR (400 MHz, CD_3OD containing 20% $CDCl_3$) δ 9.11 – 9.04 (t, *J* = 6.0 Hz, 1H), 8.35 – 8.28 (d, *J* = 8.0 Hz, 1H), 8.09 (t, *J* = 8.0 Hz, 1H), 7.98 (d, *J* =10.2 Hz, 1H), 7.34 – 7.24 (t, *J* = 9.2 Hz, 1H), 7.09 – 7.03 (d, *J* = 9.2 Hz, 1H), 6.99 (s, 1H), 6.86 (s, 1H), 6.41 – 6.36 (d, *J* = 9.2 Hz, 1H), 3.93 – 3.85 (m, 5H), 3.78 – 3.69 (m, 15H), 3.61 (t, *J* = 6.6 Hz, 2H), 3.55 (t, *J* = 6.6 Hz, 2H), 3.21 (q, *J* =9.9 Hz, 1H), 2.44 – 2.36 (m, 2H), 2.32 – 2.19 (m, 1H), 1.86 – 1.80 (m, 2H), 1.76 – 1.69 (m, 1H), 1.65 – 1.60 (m, 2H), 1.54 – 1.46 (m, 4H), 1.40 (s, 6H). Analytical HPLC: >95% purity (4.6 mm × 250 mm 5 μm C18 column; 100 μL injection; 5-95% CH_3CN/H_2O, linear gradient, with constant 0.1% v/v TFA additive; 10 min run; 1 mL/min flow; UV detection at 580 nm). HRMS (ESI): m/z calc. for $C_{44}H_{52}ClF_6N_5O_5$ $[M+H]^+$ 880.3639; found 880.3641.

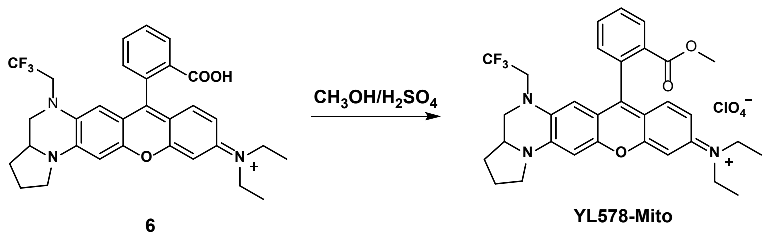

Supplementary Fig. 46 Synthesis of **YL578-Mito.**

6 (100.0 mg, 154.3 μmol, 1.0 equiv.) was dissolved in CH_3OH (2.0 mL). The reaction was stirred and H_2SO_4 (0.5 mL) was added dropwise. Then the mixture was refluxed for 6-10 h. The mixture was poured into H_2O (10.0 mL) and extracted with CH_2Cl_2 (20.0 mL) three times. The combined organic phase was washed with H_2O (10 mL) containing 0.5 mL $HClO_4$ for two times and dried over anhydrous $MgSO_4$, filtered, evaporated and the crude was purified by column chromatography on silica gel chromatography using a CH_2Cl_2/EtOH mixture as the eluent.

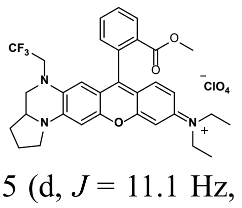

YL578-Mito: 70.5 mg. Yield:69%. Atropurpureus solid. ^{1}H NMR (400 MHz, CD_3OD containing 20% $CDCl_3$) δ 8.44 – 8.33 (t, *J* = 6.4 Hz, 1H), 7.93 (t, *J* = 7.1 Hz, 1H), 7.86 (t, *J* = 7.3 Hz, 1H), 7.44 (t, *J* = 8.4 Hz, 1H), 7.19 (t, *J* = 9.7 Hz, 1H), 7.05 – 6.97 (m, 2H), 6.85 (s, 1H), 6.23 (d, *J* = 4.4 Hz, 1H), 3.97-3.89 (m, 2H), 3.85 (d, *J* = 11.1 Hz,

3H), 3.75-3.65 (m, 8H), 3.20 (q, J = 9.9 Hz, 1H), 2.42-2.32 (m, 2H), 2.31 – 2.17 (m, 1H), 1.76 – 1.64 (m, 1H), 1.37 (d, J = 7.7 Hz, 6H). ^{13}C NMR (100 MHz, CD_3OD containing 20% $CDCl_3$) δ 166.84, 157.81, 156.83, 156.71, 155.57, 155.23, 147.46, 135.37, 135.23, 133.99, 133.89, 132.08, 131.31, 131.19, 130.96, 115.58, 114.88, 114.37, 105.63, 96.92, 96.46, 58.58, 55.15, 53.01, 46.47, 32.01, 30.76, 30.62, 23.72, 12.95. HRMS (ESI): m/z calc. for $C_{32}H_{33}F_3N_3O_3$ [M] 564.2469; found 564.2482.

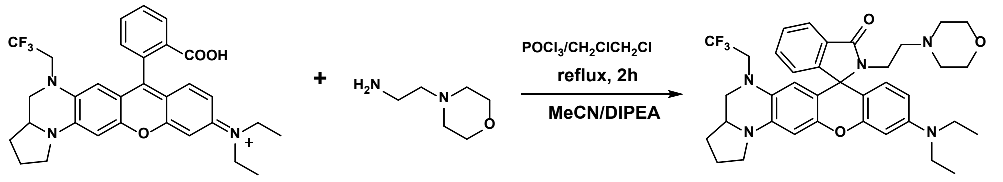

6 S-14 YL578-Lyso

Supplementary Fig. 47 Synthesis of **YL578-Lyso.**

6 (50.0 mg, 77.2 μmol, 1.0 equiv.) was dissolved in CH_2ClCH_2Cl (4.0 mL). $POCl_3$ (36 μL, 385.8 μmol, 5.0 equiv.) was added and the reaction was refluxed for 3 h. The solvent was removed under reduced pressure and the residue was dissolved in MeCN (2.0 mL). The 2-morpholin-4-ylethanamine (S-14, 30.2 mg, 231.6 μmol, 3.0 equiv.) and DIPEA (127.6 μL, 772.0 μmol, 10.0 equiv.) were dissolved in dry MeCN (2.0 mL), then transferred into the above solution. The reaction was stirred at 70 ºC for 2 h. The solvent was removed under reduced pressure to give the crude product, which was purified by silica gel flash chromatography using a CH_2Cl_2/EtOH mixture containing 0.5% triethylamine as the eluent.

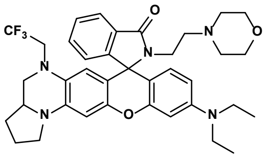

YL578-Lyso: 28.6 mg. Yield: 56%. Off-white solid. ^{1}H NMR (400 MHz, CD_3OD) δ 7.87 (d, J = 4.9 Hz, 1H), 7.58 – 7.48 (t, J = 3.2 Hz, 2H), 7.11 – 7.02 (d, J = 4.0 Hz, 1H), 6.40 (s, 1H), 6.36 (s, 2H), 6.24 (s, 1H), 5.74 – 5.68 (m, 1H), 3.54 (s, 4H), 3.47 (d, J = 9.9 Hz, 1H), 3.39 – 3.35 (m, 4H), 3.26 – 3.18 (m, 2H), 2.79 (m, 1H), 2.22 (s, 4H), 2.13 – 1.99 (m, 5H), 1.45 (s, 2H), 1.35 (d, J = 7.1 Hz, 1H), 1.29 (s, 3H), 1.15 (t, J = 6.9 Hz, 6H). ^{13}C NMR (100 MHz, CD_3OD) δ 171.08, 157.63, 157.52, 155.95, 154.96, 147.69, 135.62, 134.74, 133.26, 132.10, 131.05, 130.97, 130.81, 116.01, 114.53, 114.39, 104.75, 96.65, 95.84, 71.18, 59.18, 53.55, 52.77, 52.46, 46.22, 30.60, 23.70, 12.59. HRMS (ESI): m/z calc. for $C_{37}H_{42}F_3N_5O_3$ $[M+H]^+$ 662.3318; found 662.3317.

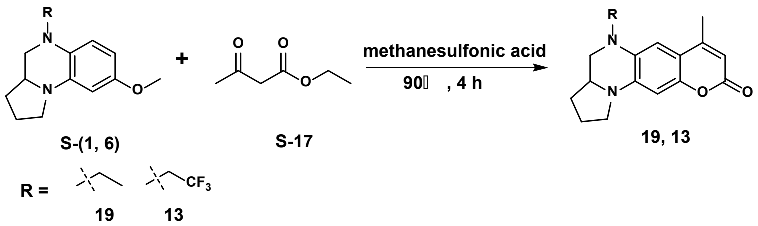

Supplementary Fig. 48 Synthesis of **13, 19.**

Ethyl acetoacetate (S-17, 61.7 mg/50.1 mg, 474.1 μmol/384.7μmol, 1.1 equiv.) and **S-1** or **S-6** (100.0 mg, 431.0 μmol/349.7 μmol, 1.0 equiv.) were dissolved in methanesulfonic acid (3.0 mL). The mixture was stirred and heated at 90°C for 3 h and

then was poured into ice water. Perchloric acid (0.5 mL) was dropwise added when the solution was stirred. The resulting mixture was extracted with CH_2Cl_2 (30.0 mL). The organic phase was dried over anhydrous $MgSO_4$, filtered, evaporated and the crude was purified by column chromatography on silica gel chromatography using an EA/PE mixture as the eluent.

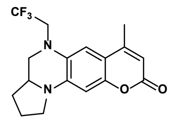

13: 83.9 mg. Yield: 71%. Orange solid. ^{1}H NMR (400 M Hz, $CDCl_3$) δ 6.69 (s, 1H), 6.29 (s, 1H), 5.95 (s, 1H), 3.97-3.86 (m, 1H), 3.83-3.72 (m, 1H), 3.55 (d, J = 9.1 Hz, 2H), 3.45-3.29 (m, 2H), 3.00-2.90 (m, 1H), 2.32 (s, 3H), 2.18 – 2.10 (m, 2H), 2.08 – 1.97 (m, 1H), 1.50-1.40 (m, 1H). ^{13}C NMR (100 MHz, $CDCl_3$) δ 162.35, 152.69, 150.11, 139.11, 129.33, 126.85, 124.04, 109.07, 105.74, 97.24, 55.31, 54.28, 53.39, 47.20, 29.92, 23.12, 18.64. MALDI-TOF/MS, m/z: calc 338.12, found 228.10.

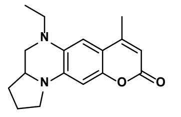

19: 78.3 mg. Yield: 64%. Red solid. ^{1}H NMR (400 MHz, $CDCl_3$) δ 6.55 (s, 1H), 6.25 (s, 1H), 5.93 (s, 1H), 3.66 (s, 1H), 3.44 (s, 3H), 3.28 (s, 2H), 2.70 (s, 1H), 2.33 (s, 3H), 2.17 – 2.08 (m, 2H), 2.06 – 1.94 (m, 1H), 1.51 – 1.41 (m, 1H), 1.23 – 1.15 (m, 3H). ^{13}C NMR (100 MHz, $CDCl_3$) δ 162.67, 152.85, 149.58, 139.67, 130.56, 108.99, 108.41, 103.57, 96.52, 56.60, 50.61, 47.32, 45.71, 30.24, 23.31, 18.80, 10.06. MALDI-TOF/MS, m/z: calc 284.15, found 284.13.

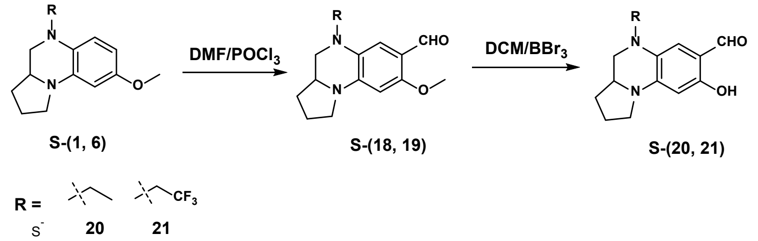

Supplementary Fig. 49 Synthesis of **S-(20, 21).**

DMF (0.5 mL) and CH_2Cl_2 (2.0 mL) was mixed in a 25 mL vial. The mixture was cooled to 0°C and stirred under N_2. And following $POCl_3$ (326.2 μL, 3.5 mmol, 3.0-4.0 equiv.) was added dropwise. The reaction was carried out at 0°C for 1 h. Then CH_2Cl_2 (2.0 mL) containing **S-1** or **S-6** (300.0 mg, 1.29 mmol/1.05 mmol, 1.0 equiv.) was added dropwise to the above mixture. the reaction was then slowly warmed to r.t. and stirred for 2 h. TLC was used to monitor the reaction. When **S-1** or **S-6** disappeared from the reaction, NaOH (aq, 0.5 mol/L, 10 mL) was added to the mixture. And the reaction was stirred at r.t. for 10 min. The mixture was extracted with CH_2Cl_2 (20.0 mL) three times. The organic phase was washed once with saturated NaCl solution and then was evaporated in vacuo. The resulting residue was subjected to fast column chromatography using an EA/PE mixture as the eluent to obtain the crude product of S-18 or S-19. And then the crude product was dissolved in CH_2Cl_2 (3.0 mL) and cooled to 0°C. BBr_3 (337.3 μL, 3.5 mmol, 3.0-4.0 equiv.) was added to the mixture. The reaction was slowly warmed to r.t. and continue stirred overnight. After completing, the mixture was poured into H_2O (10.0 mL), followed by the adjustment of pH of the solution to neutral with Na_2CO_3 aqueous solution. The mixture was extracted with EA (20.0 mL) three times. The organic phase was washed once with saturated NaCl solution, dried over anhydrous $MgSO_4$, filtered, evaporated and the crude was purified

by column chromatography on silica gel chromatography using an EA/PE mixture as the eluent.

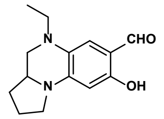

S-20: 200.4 mg. Yield: 63%. Red solid. ^{1}H NMR (400 MHz, $CDCl_3$) δ 11.66 (s, 1H), 9.42 (s, 1H), 6.45 (s, 1H), 5.83 (s, 1H), 3.67 (s, 1H), 3.45 – 3.29 (m, 3H), 3.28 – 3.17 (m, 2H), 2.62-2.50 (m, 1H), 2.15 – 2.03 (m, 2H), 2.01 – 1.91 (m, 1H), 1.48 – 1.35 (m, 1H), 1.16 (t, J = 5.8 Hz, 3H). ^{13}C NMR (100 MHz, $CDCl_3$) δ 191.65, 159.46, 144.34, 127.26, 112.62, 110.33, 96.08, 57.49, 50.34, 47.53, 45.69, 30.28, 23.45, 10.33.

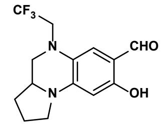

S-21: 173.1 mg. Yield: 55%. Orange solid. ^{1}H NMR (400 MHz, $CDCl_3$) δ 11.58 (s, 1H), 9.45 (s, 1H), 6.63 (s, 1H), 5.90 (s, 1H), 3.83-3.66 (m, 2H), 3.59 (s, 1H), 3.53 – 3.40 (m, 2H), 3.33 (q, J = 8.4 Hz, 1H), 2.91-2.81 (m, 1H), 2.20 – 2.08 (m, 2H), 2.06 – 1.94 (m, 1H), 1.54-1.38 (m, 1H). ^{13}C NMR (100 MHz, $CDCl_3$) δ 192.08, 159.87, 143.75, 126.95, 126.03, 115.37, 110.46, 96.83, 55.91, 54.63, 53.08, 47.53, 30.06, 23.35.

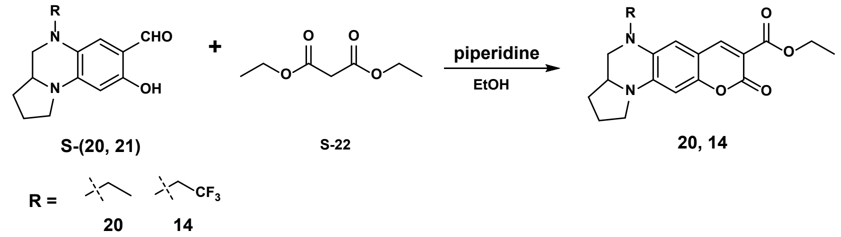

Supplementary Fig. 50 Synthesis of **20, 14.**

Diethyl malonate (S-22, 97.5 mg/80.1 mg, 609.0 μmol/500.0 μmol, 1.5 equiv.) and piperidine (118.8 μL, 1.2 mmol, 3.0-4.0 equiv.) were added to a solution of S-20 or S-21 (100.0 mg, 406.0 μmol/333.3 μmol, 1.0 equiv.) in EtOH (3.0 mL). The mixture was heated to reflux and stirred for 4 h. The solvent was evaporated in vacuo. The crude product was purified by silica gel chromatography using an EA/PE mixture as the eluent. Orange or dark red solid.

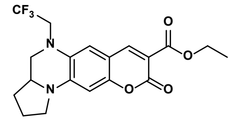

14: 106.9 mg. Yield: 81%. Yellow solid. ^{1}H NMR (400 MHz, $CDCl_3$) δ 8.40 (s, 1H), 6.64 (s, 1H), 6.27 (s, 1H), 4.36 (q, J = 7.1 Hz, 2H), 3.98-3.73 (m, 2H), 3.65 (m, 1H), 3.61-3.54 (m, 1H), 3.49 (t, J = 9.6 Hz, 1H), 3.37 (q, J = 9.6 Hz, 1H), 3.05 – 2.95 (m, 1H), 2.24-2.14 (m, 2H), 2.11 – 1.98 (m, 1H), 1.55 – 1.46 (m, 1H), 1.38 (t, J = 7.1 Hz, 3H). ^{13}C NMR (100 MHz, $CDCl_3$) δ 164.86, 159.07, 153.29, 149.11, 142.88, 131.36, 108.16, 107.90, 107.18, 95.67, 61.06, 57.28, 50.21, 47.62, 45.75, 30.34, 23.35, 14.61, 10.03. MALDI-TOF/MS, m/z: calc 396.13, found 419.14.[M+Na]

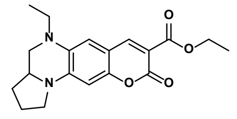

20: 115.4 mg. Yield: 83%. Orange solid. ^{1}H N MR (400 MHz, $CDCl_3$) δ 8.38 (s, 1H), 6.45 (s, 1H), 6.18 (s, 1H), 4.34 (q, J = 7.1 Hz, 2H), 3.71 (m, 1H), 3.49-3.39 (m, 3H), 3.34-3.21 (m, 2H), 2.76 – 2.66 (m, 1H), 2.22-2.10 (m, 2H), 2.08 – 1.94 (m, 1H), 1.51 – 1.43 (m, 1H), 1.37 (t, J = 7.1 Hz, 3H), 1.17 (t, J = 7.0 Hz, 3H). ^{13}C NMR (100 MHz, $CDCl_3$) δ 164.36, 158.53, 153.33, 148.97, 141.91, 129.88, 109.02, 108.98, 107.68, 96.32, 61.07, 56.02, 53.92, 53.59, 52.76, 47.43, 29.93, 23.05, 14.40. MALDI-TOF/MS, m/z: calc 342.16, found

342.13.

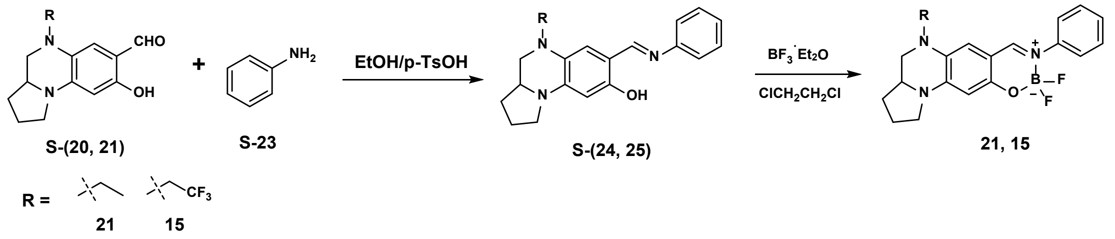

Supplementary Fig. 51 Synthesis of **21, 15.**

EtOH (2.0 mL) dissolving S-20 or S-21 (100.0 mg, 406.0 μmol/333.3 μmol, 1.0 equiv.) was added to a solution of phenylamine (S-23, 55.5 μL/45.6 μL, 609.0 μmol/500.0 μmol, 1.5 equiv.) and p-TsOH (6.4 mg/5.3 mg, 40.6 μmol/33.3 μmol, 0.1 equiv.) in ethanol (4.0 mL). The mixture refluxed for 4 h and then the solvent was evaporated in vacuo. The resulting residue was dissolved with CH_2ClCH_2Cl (4.0 mL). $BF_3 \cdot Et_2O$ (812.0 μmol/666.6 μmol, 2.0 equiv.) was added to the above solution. And the mixture was stirred at r.t. for 3 h. DIPEA (165.3 μL/137.7 μL, 1.0 mmol/833.3 μmol, 2.5 equiv.) was added. The mixture was continuously stirred until the color of the solution became yellow or orange. The mixture was poured into H_2O (10.0 mL), followed by the adjustment of pH of the solution to neutral with Na_2CO_3 aqueous solution. The mixture was extracted with EA (10.0 mL) three times. The organic phase was washed once with saturated NaCl solution, dried over anhydrous $MgSO_4$, filtered, evaporated and the crude was purified by column chromatography on silica gel chromatography using an EA/PE mixture as the eluent. Orange or dark red solid.

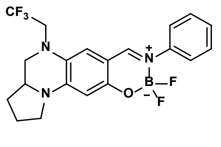

15: 122.7 mg. Yield: 87%. Yellow solid. ^{1}H NMR (400 MHz, $CDCl_3$) δ 7.99 (s, 1H), 7.48 (d, J = 7.6 Hz, 2H), 7.40 (t, J = 7.5 Hz, 2H), 7.3 1 (d, J = 6.8 Hz, 1H), 6.50 (s, 1H), 6.03 (s, 1H), 3.87 – 3.59 (m, 3H), 3.56-3.44 (m, 2H), 3.37 (q, J = 9.3 Hz, 1H), 2.99-2.87 (m, 1H), 2.23-2.10 (m, 2H), 2.10 – 1.93 (m, 1H), 1.55 – 1.41 (m, 1H). ^{13}C NMR (100 MHz, $CDCl_3$) δ 158.13, 157.89, 146.27, 143.54, 129.48, 127.54, 126.88, 124.07, 123.33, 111.69, 106.34, 97.92, 56.54, 54.33, 52.93, 47.76, 30.03, 23.22. MALDI-TOF/MS, m/z: calc 423.15, found 423.15.

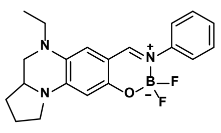

21: 110.7 mg. Yield: 74%. Orange solid. ^{1}H NMR (400 MHz, $CDCl_3$) δ 7.98 (s, 1H), 7.50 (d, J = 6.3 Hz, 2H), 7.40 (s, 2H), 7.29 (s, 1H), 6.33 (s, 1H), 6.01 (s, 1H), 3.75 (s, 1H), 3.51 (s, 1H), 3.38 (s, 2H), 3.24 (s, 1H), 2.67 (s, 1H), 2.15 (s, 2H), 2.02 (s, 1H), 1.49 (s, 1H), 1.26 (s, 2H), 1.17 (s, 3H). ^{13}C NMR (100 MHz, $CDCl_3$) δ 158.03, 157.20, 147.09, 143.81, 129.42, 128.80, 127.20, 123.30, 109.38, 106.62, 97.36, 57.84, 50.31, 47.78, 45.67, 30.30, 23.36, 10.12. MALDI-TOF/MS, m/z: calc 369.18, found 369.22.

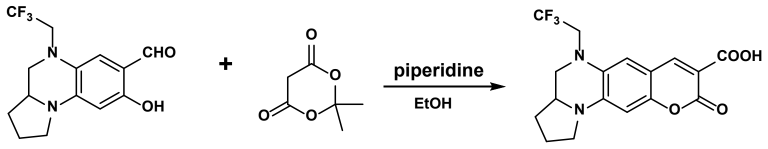

S-21 S-26 16

Supplementary Fig. 52 Synthesis of **16.**

Meldrum's acid (S-26, 72.1 mg, 500 μmol, 1.5 equiv.) and piperidine (99.0 μL, 1.0 mmol, 3.0 equiv.) were added to a solution of S-21 (100.0 mg, 333.3 μmol, 1.0 equiv.) in ethanol (3.0 mL). The mixture was heated to reflux and stirred for 4 h. The resulting solution was acidified with hydrochloric acid. The mixture was extracted with CH_2Cl_2 (20.0 mL) three times. The combined organic phase was washed with brine, dried with $MgSO_4$, filtered, and concentrated in vacuo. The residue was purified by silica gel flash chromatography using a CH_2Cl_2/EtOH mixture as the eluent.

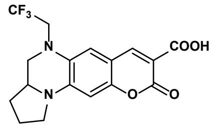

16: 95.7 mg. Yield: 78%. Yellow solid. ^{1}H NMR (400 MHz, $CDCl_3$) δ 12.48 (s, 1H), 8.52 (s, 1H), 6.64 (s, 1H), 6.31 (s, 1H), 3.97 (m, 1H), 3.89 – 3.77 (m, 1H), 3.71 (s, 1H), 3.64-3.51 (m, 2H), 3.48-3.38 (m, 1H), 3.06 (t, J = 7.6 Hz, 1H), 2.28 – 2.18 (m, 2H), 2.16 – 2.05 (m, 1H), 1.55 (s, 1H). ^{13}C NMR (100 MHz, $CDCl_3$) δ 165.78, 164.87, 153.21, 149.71, 143.19, 131.02, 126.75, 108.98, 108.75, 105.54, 96.43, 56.58, 53.49, 52.59, 47.88, 30.12, 23.23. HRMS (ESI): m/z calc. for $C_{17}H_{15}F_3N_2O_4$ [M+H]+ 369.1062; found 369.1065.

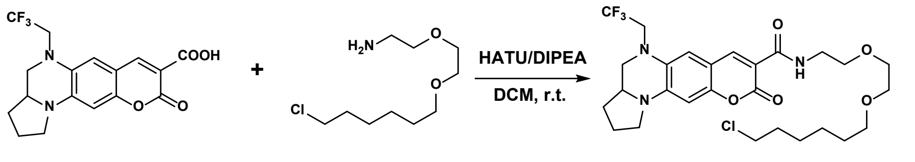

16 S-10 16-Halo

Supplementary Fig. 53 Synthesis of **16-Halo.**

16 (50.0 mg, 135.9 μmol, 1.0 equiv.) was dissolved in CH_2Cl_2 (4.0 mL). DIPEA (112.3 μL, 679.3 μmol, 5.0 equiv.) and HATU (103.3 mg, 271.8 μmol, 2.0 equiv.) were added and the mixture was stirred at r.t. for 10 min. S-10 (48.3 mg, 203.9 μmol, 1.5 equiv.) was added and the reaction was continue stirred at r.t. for 4 h. The solvent was removed and the residue was purified by silica gel flash chromatography using EA/PE mixture as the eluent.

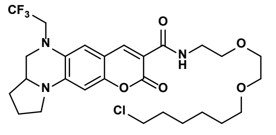

16-Halo: 53.7 mg. Yield 69%. Yellow-brown s olid. ^{1}H NMR (400 MHz, CD_3OD) δ 9.25 (s, 1H), 8.40 (s, 1H), 6.75 (s, 1H), 6.12 (s, 1H), 4.16-3.89 (m, 2H), 3.67 – 3.63 (m, 5H), 3.62 – 3.54 (m, 8H), 3.48 (m, 4H), 2.93 – 2.83 (m, 1H), 2.17 – 2.10 (m, 2H), 2.07 – 1.98 (m, 1H), 1.73 – 1.67 (m, 2H), 1.58 – 1.50 (m, 3H), 1.40 – 1.35 (m, 3H). ^{13}C NMR (100 MHz, CD_3OD) δ 165.67, 164.12, 153.81, 148.86, 143.75, 132.05, 128.74, 125.93, 109.95, 109.67, 96.63, 72.43, 71.72, 71.40, 70.73, 57.71, 53.62, 45.87, 40.74, 33.93, 30.95, 30.80, 27.95, 26.66, 24.11. HRMS (ESI): m/z calc. for $C_{27}H_{35}ClF_3N_3O_5$ $[M+H]^+$ 574.2296; found 574.2303.

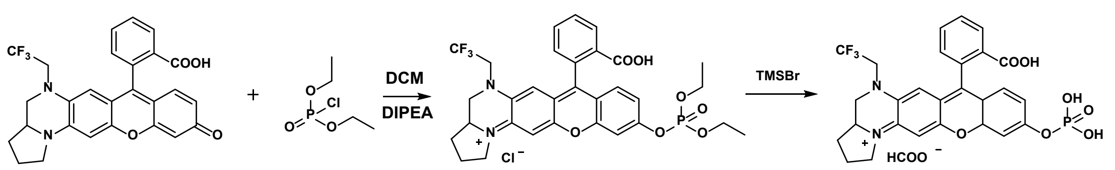

11 S-27 S-28 11-ALP

Supplementary Fig. 54 Synthesis of **11-ALP.**

11 (100.0 mg, 202.4 μmol, 1.0 equiv.) was dissolved in CH_2Cl_2 (4.0 mL). DIPEA (100.4 μL, 607.3 μmol, 3.0 equiv.) and diethyl chlorophosphate (S-27, 52.4 mg, 303.6 μmol, 1.5 equiv.) were added. The reaction was stirred at r.t. for 4 h. Ice water (2.0 mL) was added to quench the reaction. The mixture was extracted with CH_2Cl_2 (20 mL) three times. The combined organic phase was washed with brine, dried with $MgSO_4$, filtered, and concentrated in vacuo. The residue was dissolved in CH_2Cl_2 (5.0 mL) and bromo(trimethyl)silane (TMSBr, 464.8 mg, 3.0 mmol, 15.0 equiv.) was added dropwise under N_2 atmosphere. The mixture was stirred overnight at room temperature and monitored by TLC. When the reaction was complete, the intermediate product was obtained by removing the organic solvent in vacuum. **11-ALP** was obtained by silica gel flash chromatography using a CH_2Cl_2/EtOH mixture (containing 0.5% HCOOH) as the eluent.

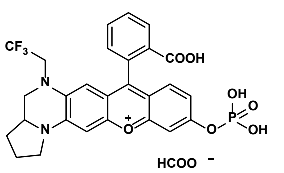

11-ALP: 41.5 mg. Yield: 33%. Red solid. ^1H NMR (4 00 MHz, CD_3OD containing 20% $CDCl_3$) δ 8.37 (s, 1H), 8.02 – 7.91 (s, 1H), 7.88 – 7.76 (t, J = 7.6 Hz, 2H), 7.44 – 7.16 (m, 3H), 6.93 (s, 1H), 6.22 (d, J = 6.1 Hz, 1H), 4.05 – 3.88 (m, 3H), 3.87 – 3.74 (m, 3H), 3.21 (m, 1H), 2.99 (s, 1H), 2.86 (s, 1H), 2.39 – 2.24 (m, 2H), 2.23 – 2.13 (m, 1H), 1.73 – 1.65 (m, 1H). Analytical HPLC: >95% purity (4.6 mm × 250 mm 5 μm C18 column; 100 μL injection; 5-95% CH_3CN/H_2O, linear gradient, with constant 0.1% v/v TFA additive; 10 min run; 1 mL/min flow; UV detection at 560 nm). HRMS (ESI): m/z calc. for $C_{27}H_{23}F_3N_2O_7P$ $[M+H]^+$ 576.1262; found 576.1251.

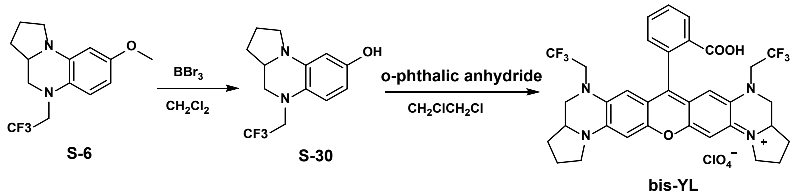

Supplementary Fig. 55 Synthesis of **bis-YL.**

S-6 (100 mg, 350.0 μmol, 1.0 equiv.) was dissolved in CH_2Cl_2 (4.0 mL). BBr_3 (40 μL, 454.5 μmol, 1.3 equiv.) was added dropwise and the mixture was stirred at r.t. for 2 h. H_2O (5.0 mL) was added to quench the reaction. Saturated sodium bicarbonate solution was carefully added to adjust the solution to neutral. The mixture was extracted with CH_2Cl_2 (5.0 mL) three times. The combined organic phase was washed with brine, dried with $MgSO_4$, filtered, and concentrated in vacuo. Since the demethylation product was unstable, the residue was directly put into the next step. The residue was dissolved in CH_2ClCH_2Cl (4.0 mL). o-phthalic anhydride (25.9 mg, 175.0 μmol, 0.5 equiv.) and A drop of concentrated sulfuric acid were added. The mixture was refluxed for 48 h. the reaction was diluted with H_2O (5.0 mL) and extracted with CH_2Cl_2 (10.0 mL) three times. The combined organic phase was washed with brine (10.0 mL) containing 0.5 mL $HClO_4$, dried with $MgSO_4$, filtered, and concentrated in vacuo. The mixture was purified by flash chromatography on silica gel using a CH_2Cl_2/EtOH mixture as the eluent.

bis-YL: 3.2 mg. yield 2.4%. Atropurpureus solid. ^1H NMR (400 MHz, CD_3OD) δ

8.25 (s, 1H), 7.75 (s, 2H), 7.31 (d, J = 6.9 Hz, 1H), 6.77 (s, 2H), 6.38 (t, J = 9.9 Hz, 2H), 4.08 – 3.97 (m, 1H), 3.86 (d, J = 6.0 Hz, 2H), 3.79 – 3.69 (m, 6H), 3.64 (s, 1H), 3.62 – 3.55 (m, 2H), 3.11 – 2.98 (m, 2H), 2.24 (m, 4H), 2.18 – 2.07 (m, 2H), 1.65 – 1.55 (m, 2H). Analytical HPLC: >95% purity (4.6 mm × 250 mm 5 μm C18 column; 100 μL injection; 5-95% CH_3CN/H_2O, linear gradient, with constant 0.1% v/v TFA additive; 10 min run; 1 mL/min flow; UV detection at 580 nm). HRMS (ESI): m/z calc. for $C_{31}H_{29}F_3N_3O_3$ [M] 657.2295; found 657.2296.

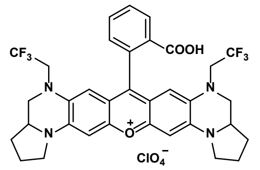

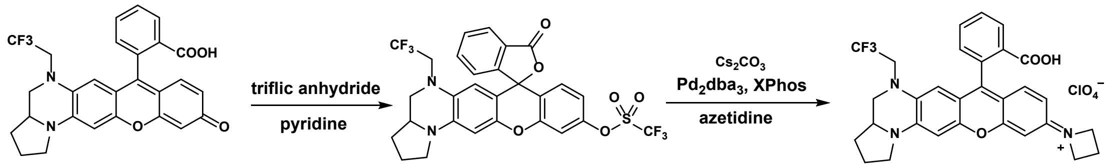

11

S-31

YL-Az

Supplementary Fig. 56 Synthesis of **S-31**.

A vial was charged with 11 (200 mg, 0.4 mmol, 1.0 equiv.). CH_2Cl_2 (5 mL) was added. The reaction was flushed again with nitrogen (3×) and stired at 0 °C. Following the addition of pyridine (98.0 μL, 3 equiv.) and triflic anhydride (136.8 mg, 81.6 μL, 0.48 mmol, 1.2 equiv.), the reaction was stirred at 0 °C for 5 min. It was then restored to r.t. and kept stirring for 4 h. The mixture was added H_2O to quench reaction, extracted with CH_2Cl_2 (10 mL) for three times. The combined organic phase was washed with saturated $CuSO_4$ aqueous solution three times and saturated NaCl aqueous solution one time, dried with $MgSO_4$, filtered and concentrated in vacuo. The residue was purified by silica gel flash chromatography using a CH_2Cl_2/MeOH mixture as the eluent.

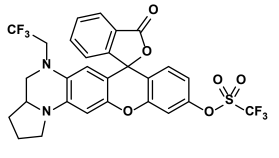

S-31: 87.0 mg. Yield: 34.4 %. Light yellow solid. ^{1}H NMR (400 MHz, $CDCl_3$) δ 8.03 (d, J = 7.6 Hz, 1H), 7.66 (m, 2H), 7.22 – 7.16 (m, 2H), 6.92 – 6.84 (m, 2H), 6.27 (d, J = 5.0 Hz, 1H), 5.83 (d, J = 4.0 Hz, 1H) 3.58 – 3.48 (m, 1H), 3.48 – 3.27 (m, 5H), 2.89 (m, 1H), 2.11 (m, 2H), 2.06 – 1.96 (m, 1H), 1.78 – 1.54 (m, 1H). ^{13}C NMR (100 MHz, $CDCl_3$) δ 169.72, 153.03, 150.22, 146.29, 138.50, 135.54, 130.54, 130.41, 129.94, 127.19, 125.54, 124.44, 120.74, 120.01, 117.55, 116.34, 110.70, 109.91, 109.05, 104.47, 97.73, 97.57, 77.50, 56.00, 55.38, 54.66, 47.62, 30.35, 23.61. MALDI-TOF/MS, m/z: $C_{28}H_{21}F_6N_2O_6S$ [M] calc 627.10, found 627.07.

Supplementary Fig. 57 Synthesis of **YL-Az.**

A vial was charged with **S-31** (50 mg, 80 μmol, 1.0 equiv.), Pd_2dba_3 (7.7 mg, 8.0 μmol, 0.1 equiv.), and XPhos (12.0 mg, 24.0 μmol, 0.3 equiv.), and Cs_2CO_3 (52.0 mg, 160.0 μmol, 2.0 equiv.). The vial was sealed and evacuated/backfilled with nitrogen (3×). Dioxane (1 mL) was added, and the reaction was flushed again with nitrogen (3×). Following the addition of azetidine (9.1 mg, 160.0 μmol, 2.0 equiv.), the reaction was stirred at 100 °C for 18 h. It was then cooled to room temperature, diluted with H_2O (5.0 mL, containing 100 μL $HClO_4$), extracted with CH_2Cl_2 (5 mL) for three times. The combined organic phase was washed with brine(10.0 mL) containing 0.5 mL $HClO_4$, dried with $MgSO_4$, filtered, and concentrated in vacuo. The residue was purified by silica gel flash chromatography using a CH_2Cl_2/EtOH mixture as the eluent.

YL-Az: 6.5 mg. Yield: 12.8%. Atropurpureus solid. ^{1}H NMR (400 MHz, CD_3OD) δ 7.76 – 7.64 (m, 3H), 7.38 (s, 1H), 7.08 (t, *J* = 8.7 Hz , 1H), 6.72 (d, *J* = 9.4 Hz, 1H), 6.67 (d, *J* = 7.1 Hz, 2H), 6.36 (d, *J* = 9.9 Hz, 1H), 4.19 (m, 1H), 4.09 – 4.01 (m, 1H), 3.94 – 3.88 (m, 1H), 3.76 (m, 2H), 3.69 (m, 2H), 3.58 (d, *J* = 9.0 Hz, 1H), 3.06 (m, 1H), 2.28 – 2.21 (m, 2H), 2.19 – 2.11 (m, 1H), 1.64 – 1.54 (m, 1H), 1.26 (s, 4H). Analytical HPLC: >95% purity (4.6 mm × 250 mm 5 μm C18 column; 100 μL injection; 5-95% CH_3CN/H_2O, linear gradient, with constant 0.1% v/v TFA additive; 10 min run; 1 mL/min flow; UV detection at 560 nm). HRMS (ESI): m/z calc. for $C_{31}H_{29}F_3N_3O_3$ [M] 534.1999; found 534.2003.

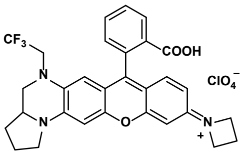

Measurements of UV-Vis, fluorescence spectroscopy, and quantum yields. Fluorescent molecules for spectroscopy were prepared as stock solutions (0.1 – 10 mM) in DMSO. Maximum absorption wavelength (λ_{abs}) and maximum emission wavelength (λ_{em}) were taken in 20 mM PBS buffer unless otherwise noted; reported values are averages of 3 measurements.

Calculation of fluorescence quantum yield. Fluorescence quantum yield was determined using an optically matching solution of cresol purple (Φ_f = 0.58 in ethanol)[6], RhB (Φ_f = 0.65 in ethanol)[7] or coumarin 102 (Φ_f = 0.93 in ethanol)[8] as the standard and the quantum yield was calculated using the following equation:

$$\Phi_s = \Phi_r(A_rF_s/A_sF_r)\ (n_s^2/n_r^2)$$

where, s and r denote sample and reference, respectively. A is the absorbance. F is the relative integrated fluorescence intensity and n is the refractive index of the solvent. Measurements were performed using dilute samples (A ≈ 0.05) and reported values are averages (n=3). Reference fluorophore: **1-6, 8, 11-14**, **YL-Az**, **bis-YL**, cresol purple; **7**, RhB; **16-20**, R 4-6, coumarin 102. R 1-3: According to the reported literatures[9, 10, 11].

Photostability of YL-6 and RhB. Solutions of 10 μM **6 (YL578)** and RhB in EtOH containing 0.1% trifluoracetic acid (TFA) were prepared. The solution was filled with oxygen for 10 min. The absorbance spectra were recorded using cock threaded quartz cuvette on a UV-1800 spectrophotometer (Shimadzu Corporation, Japan). The measurements were carried out under the irradiation of a laser at 530 nm (1W) at ambient temperature. The solution was continuously irradiated and the absorbance of the solution was recorded every 20 min. Absorbance was normalized to the maximum absorbance of each fluorophore and plotted against radiation time. Reported values are averages of 3 measurements.

Computational Methods. The calculations were carried out using the Gaussian 09 program package[12]. The geometries of dyes were optimized at the B3LYP/6-31+G(d) level using a CPCM solvation model with water as the solvent. Potential energy surfaces of diethylamino in **YL578** and RhB with increasing twist angle (φ) were calculated with CAM-B3LYP method at the LRC-TDDFT/6-31+G(d) level[13]. The rotation step is 10° in the range of 0°–90°. Coordinate-driven potential surface scans

were generated by fixing the bridge dihedral angle (Figure 2h) and minimizing all other degrees of freedom subject to this constraint.

Photostability of YLs in living cells. HeLa cells were incubated with DMEM containing 5 μM fluorophores for 30 min and next continuously illuminated under the confocal laser and imaged at a fixed time point (10-30 min). The relative mean intracellular fluorescence of cells was utilized for quantification and normalization. Error bars, ± s.e.m. from about 50 cells.

Measurements of UV absorbance spectra in water-dioxane mixtures. Solutions of 5 μM **9** and **10** in water-dioxane mixtures containing 10%, 20%, 30%, 40%, 50%, 60%, 70%, 80% and 90% of dioxane (by volume) were prepared. The absorbance spectra were recorded using a quartz cuvette on a UV-1800 spectrophotometer (Shimadzu Corporation, Japan). The measurements were taken at ambient temperature. Absorbance was normalized to the maximum absorbance of each carboxyl-fluorophores. Normalized absorbance was plotted against the dielectric constant of the water-dioxane mixture[14].

Measurement of absorption, emission spectra, and quantum yields after binding with proteins. Fluorogenic probes were diluted to 2.5 μM in 10 mM HEPES, pH 7.3. Then an aliquot of HaloTag protein (2.0 equiv.) was added and the resulting mixture was incubated for 1h. Absorbance measurements were performed in V-770 Spectrophotometer (Jasco), while fluorescence measurements were performed in a 96-well plate (Thermo Fisher Scientific) with an optical bottom. Reported values are averages of 3 measurements.

Time-dependent live-cell imaging. Live HeLa H2B-Halo-expressed cells were incubated with **YL578-Halo** and **10-Halo** (250 nM) and directly imaged every 0.5-1 h. Intracellular brightness of all the cells in the view field was recorded and averaged. Error bars, ± s.e.m. from about 50 cells.

Multi-colour confocal and STED imaging. U-2 OS stably expressing Vimentin-HaloTag cells seeded on glass coverslips were incubated in phenol red-free imaging medium that contained 500 nM **YL578-Halo** overnight at 37 °C and then cells were washed with DMEM without phenol red and FBS. Afterwards, the cells were incubated in phenol red-free imaging medium that contained 500 nM SiR-DNA or GeR-tubulin for 3 h, washed in DMEM without phenol red and FBS. The samples were imaged on an Infinity Line STED microscope equipped with 775 and 660 STED lines, and 518 nm, 561 nm, 640 nm, and multiphoton excitation lines (Abberior Instruments GmbH). Imaging conditions: for **YL578-Halo**, λ_{ex} 561 nm, detection range 570–700 nm, STED laser 775 nm; for GeR-tubulin and SiR-DNA, λ_{ex} 640 nm, detection range 655–760 nm, STED laser 775 nm. For three-colour STED imaging, after incubing cells with **YL578-Halo** and washing, the cells were labelled in phenol red-free imaging medium that contained 500 nM MaP555-actin and GeR-tubulin for 3 h, washed in DMEM without

phenol red and FBS. The samples were imaged on an Infinity Line STED microscope equipped with 775 and 660 STED lines, and 518 nm, 561 nm, 640 nm, and multiphoton excitation lines (Abberior Instruments GmbH). Imaging conditions: for **YL578-Halo**, λ_{ex} 561 nm, detection range 570–700 nm, STED laser 775 nm; for GeR-tubulin, λ_{ex} 640 nm, detection range 655–760 nm, STED laser 775 nm; for MaP555-actin, λ_{ex} 518 nm, detection range 540-565 nm, STED laser 775 nm.

Two-photon microscopy. U-2 OS stably expressing Vimentin-HaloTag cells seeded on glass coverslips were incubated in phenol red-free imaging medium that contained 50 nM **YL578-Halo** for 6 h at 37 °C. The cells were fixed with 4% PFA for 20 min and then quenched for 5 min in NH_4Cl and glycine (100 mM), washed in PBS, and mounted in Mowiol. Imaging was performed on an Abberior multiphoton Infinity Line microscope. Imaging conditions: λ_{ex} 870 nm, detection range 570–780 nm, STED laser 775 nm.

NMR and HPLC spectroscopy

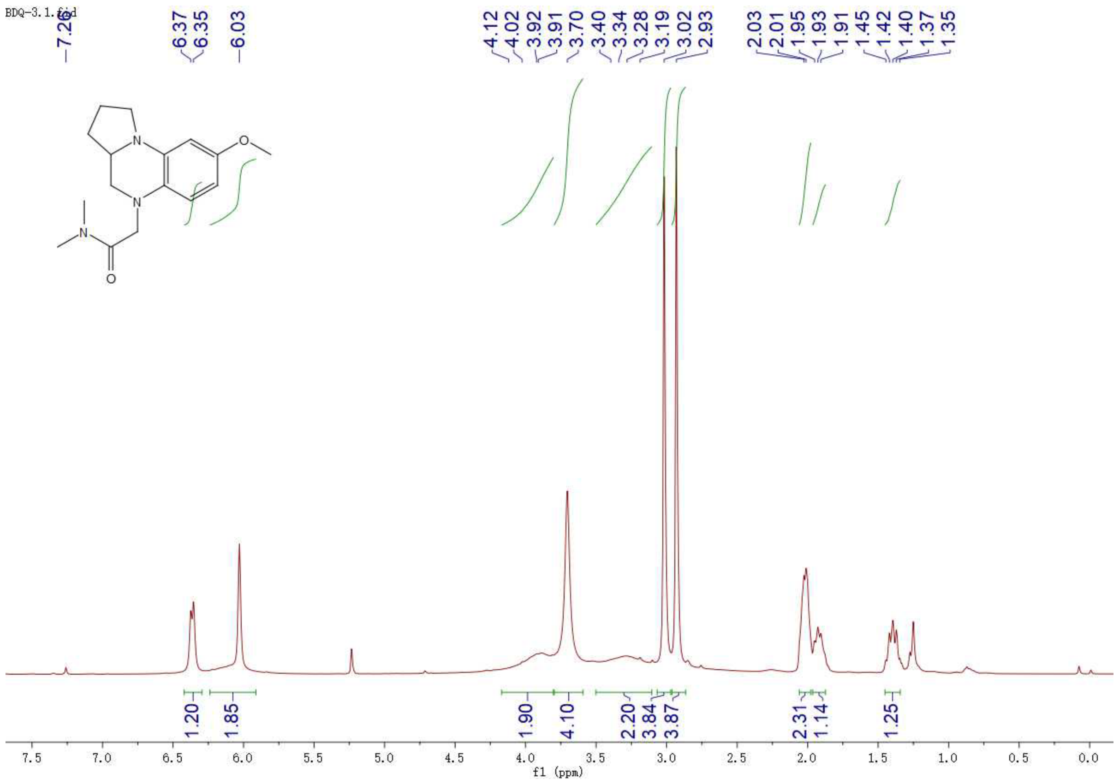

^{1}H NMR spectrum of **S-3** in $CDCl_3$

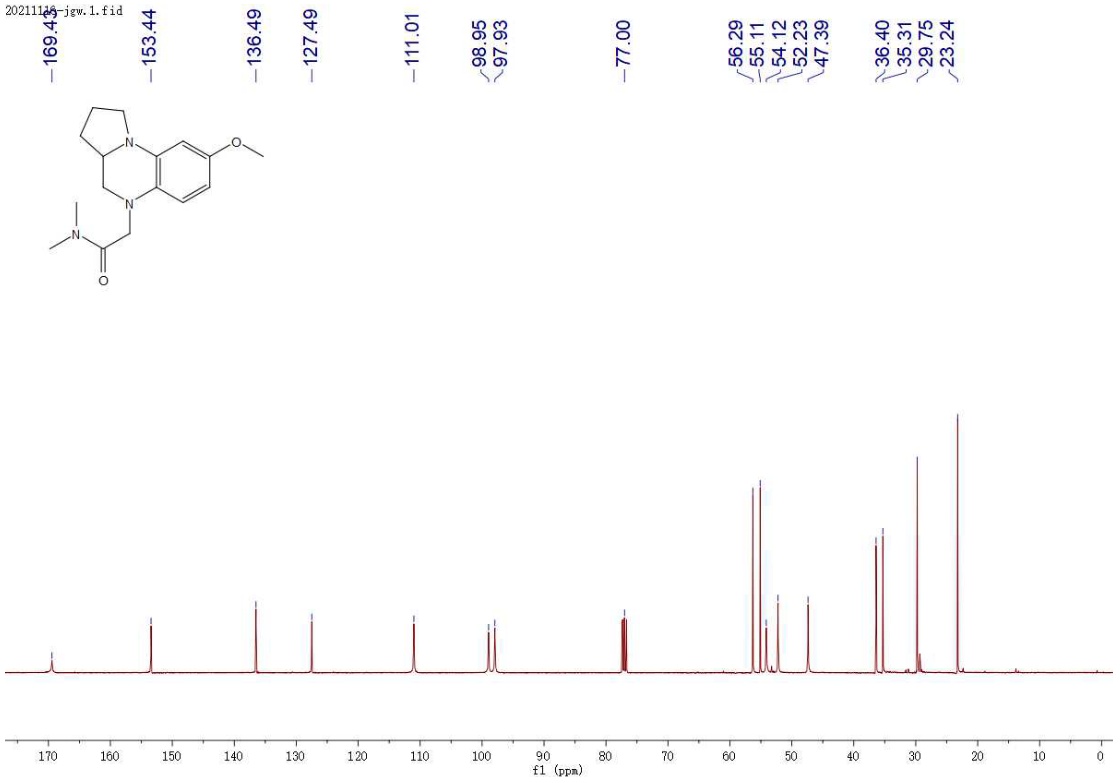

^{13}C NMR spectrum of **S-3** in $CDCl_3$

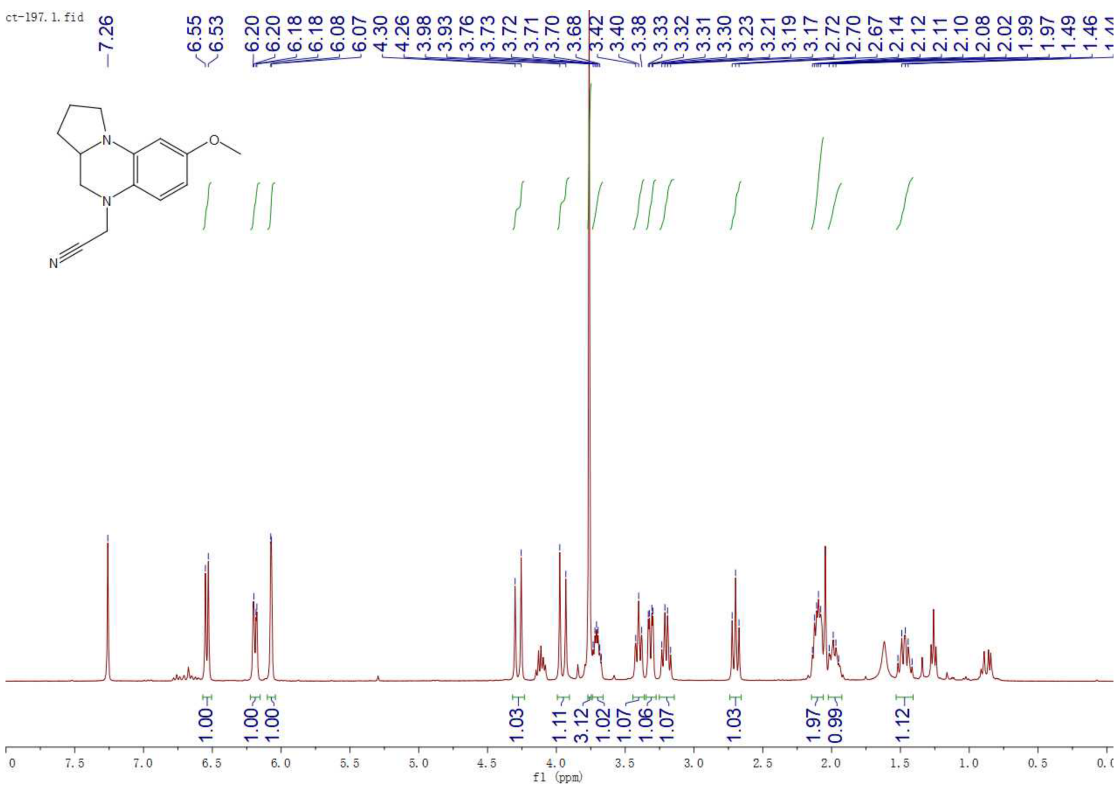

^{1}H NMR spectrum of **S-4** in $CDCl_3$

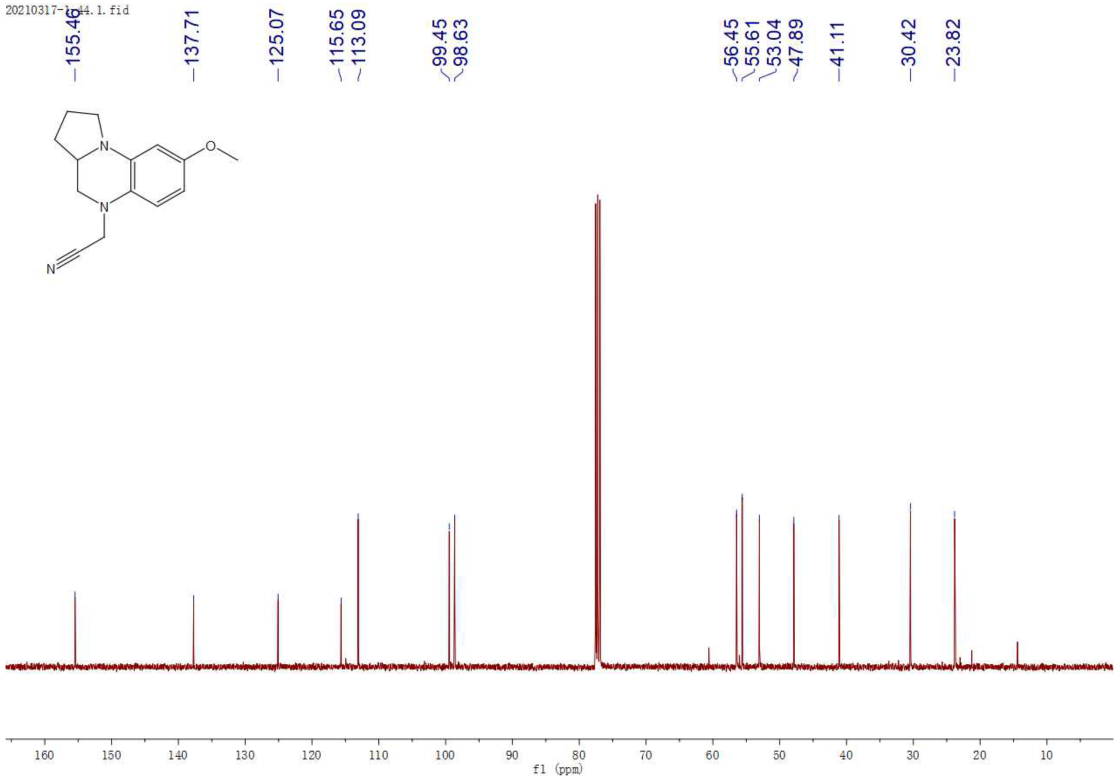

^{13}C NMR spectrum of **S-4** in $CDCl_3$

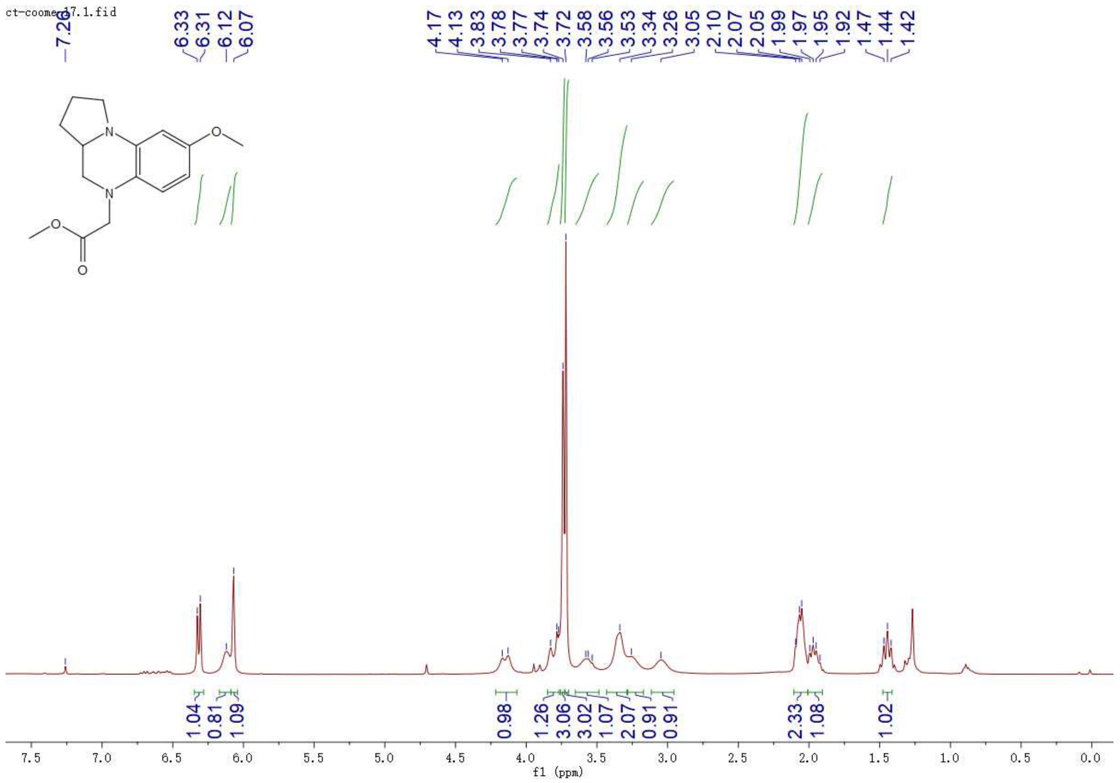

^{1}H NMR spectrum of **S-5** in $CDCl_3$

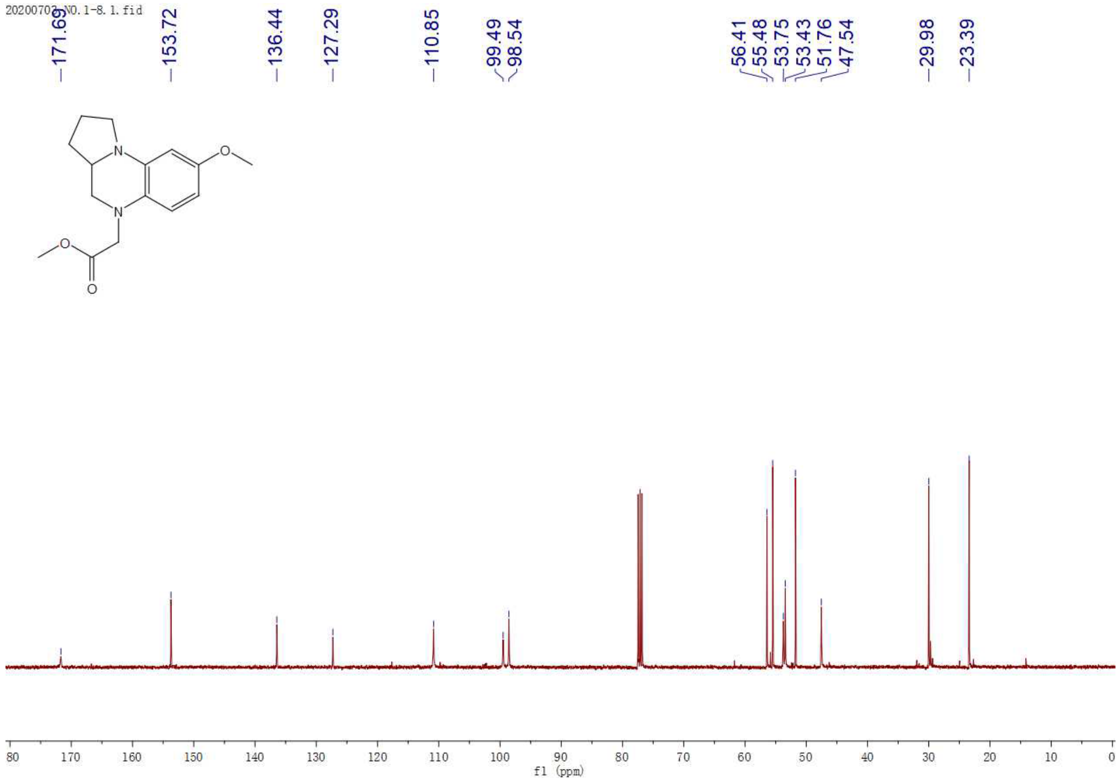

^{13}C NMR spectrum of **S-5** in $CDCl_3$

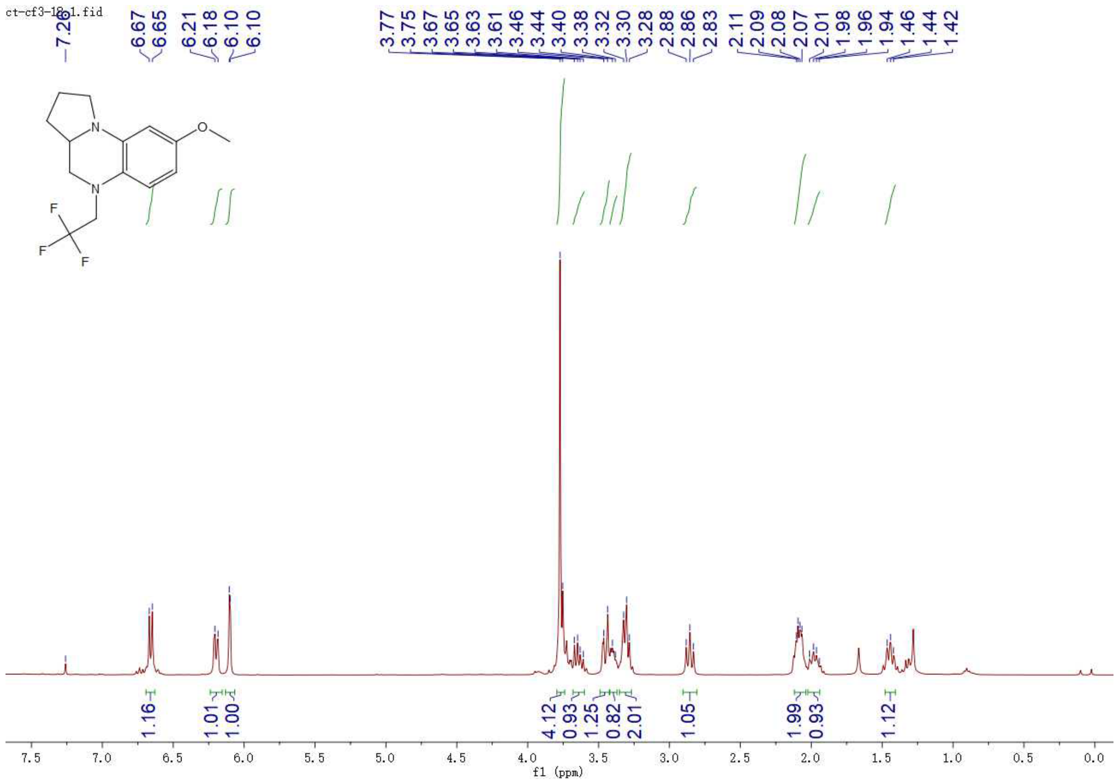

^{1}H NMR spectrum of **S-6** in $CDCl_3$

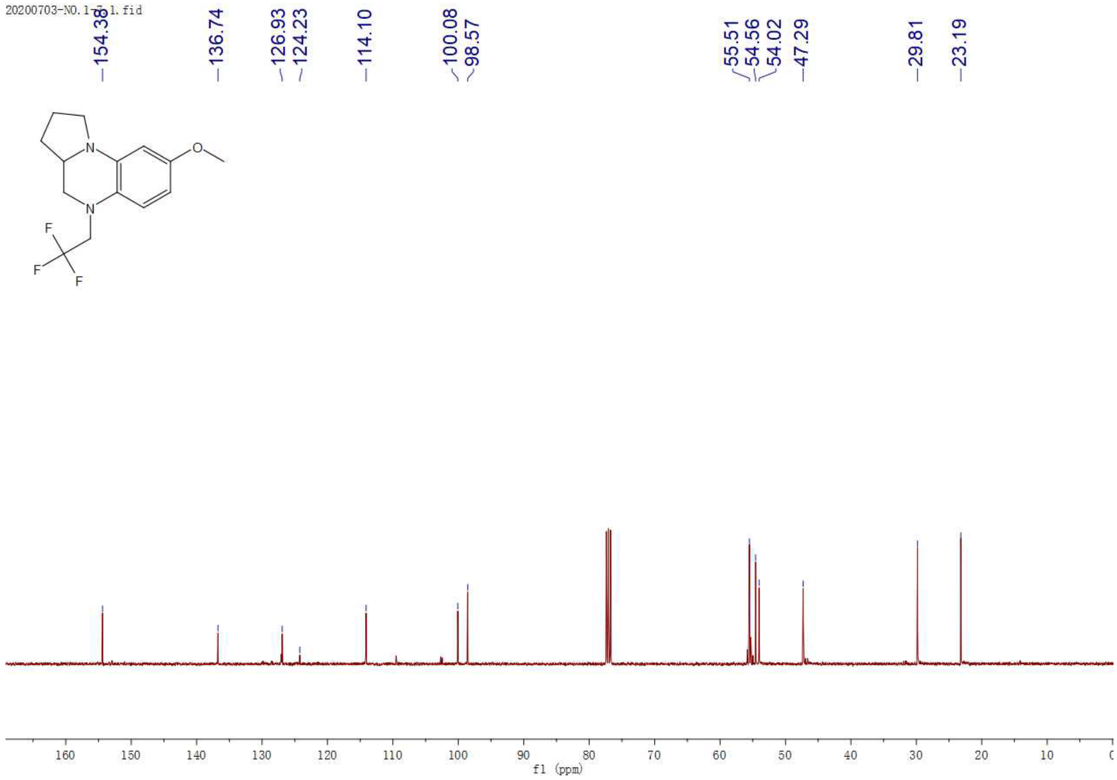

^{13}C NMR spectrum of **S-6** in $CDCl_3$

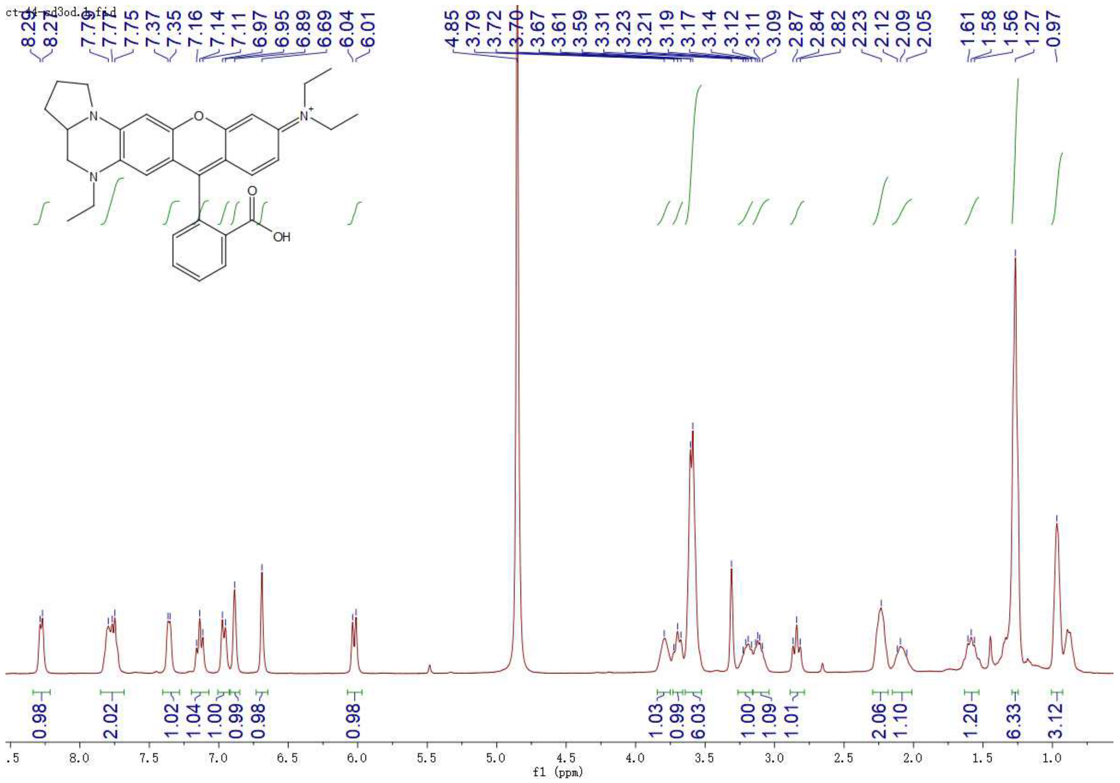

^{1}H NMR spectrum of **1** in CD_3OD

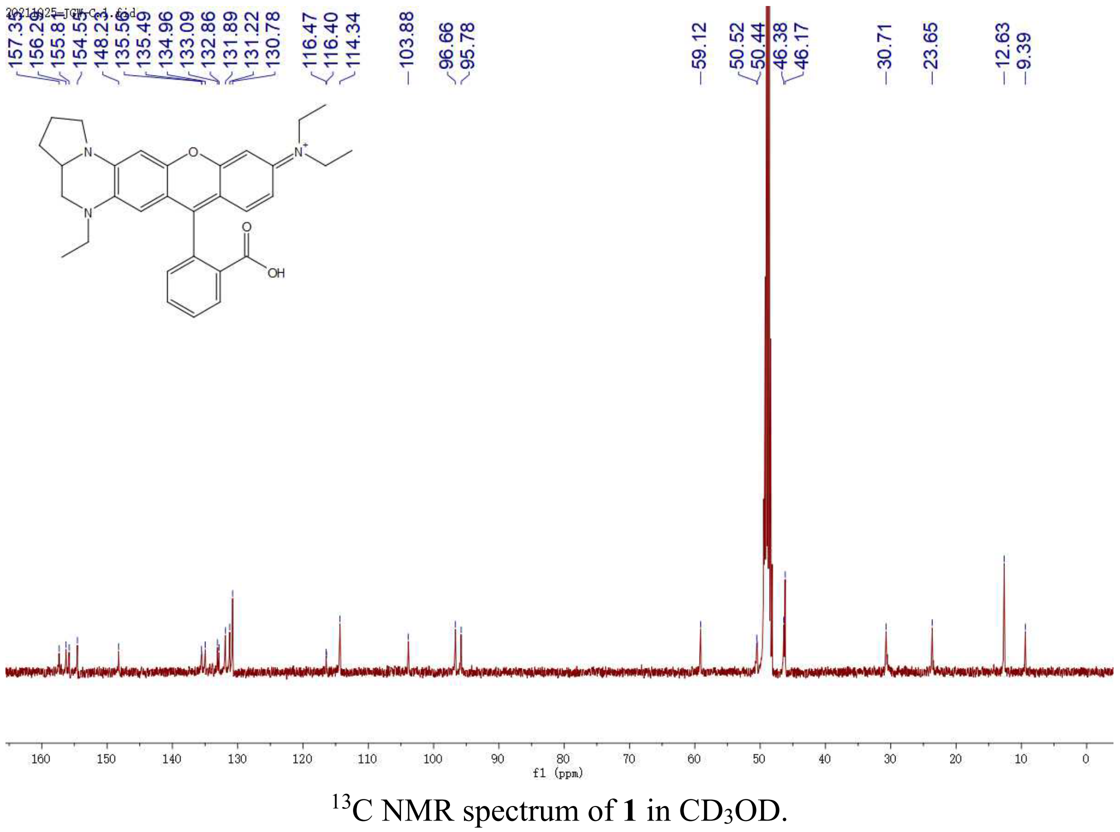

^{13}C NMR spectrum of **1** in CD_3OD.

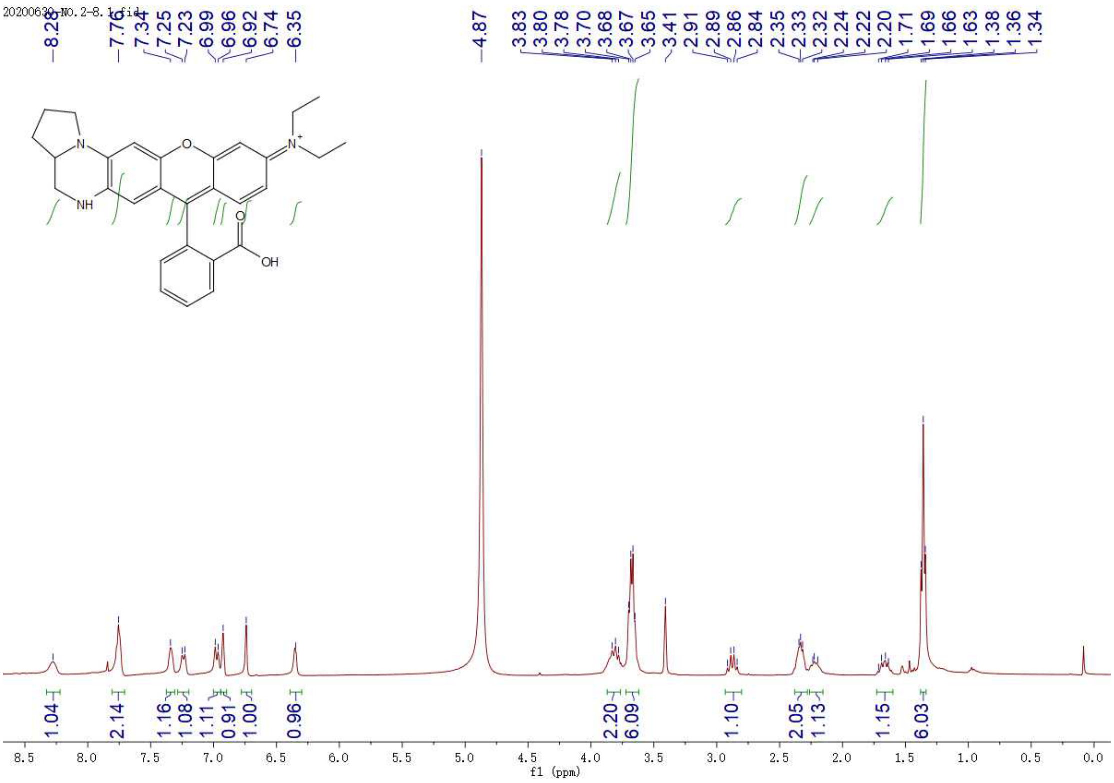

^{1}H NMR spectrum of **2** in CD_3OD containing 20 % $CDCl_3$

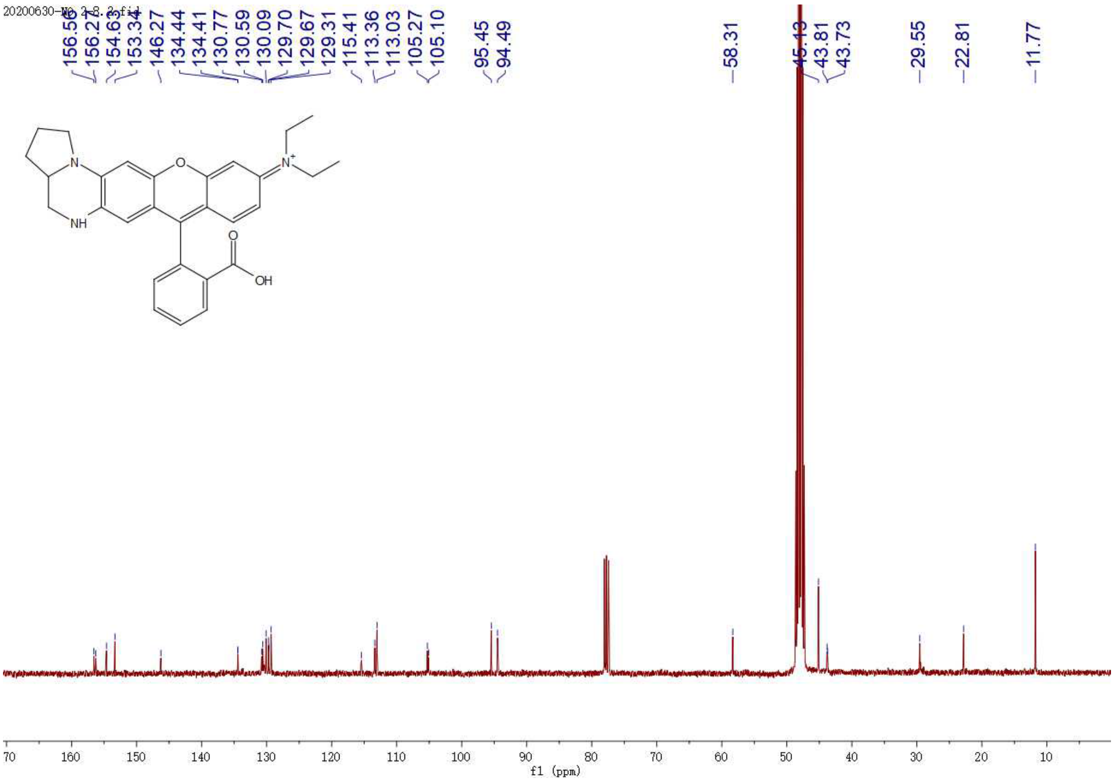

^{13}C NMR spectrum of **2** in CD_3OD containing 20 % $CDCl_3$

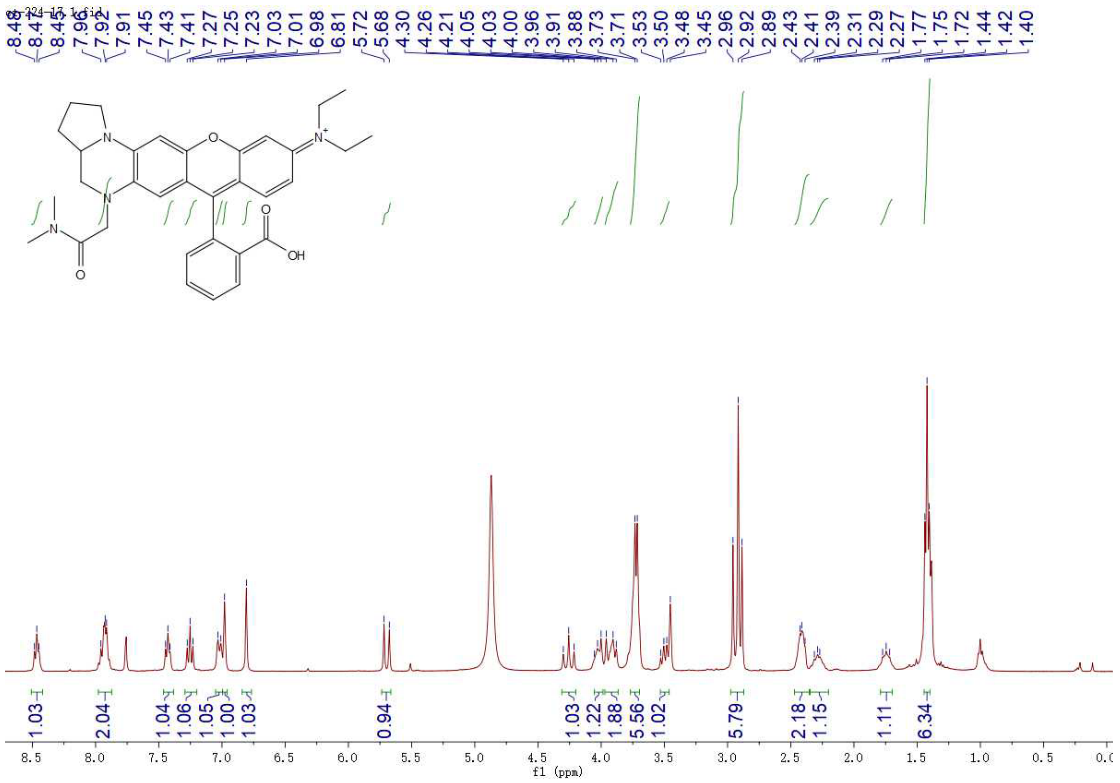

^{1}H NMR spectrum of **3** in CD_3OD containing 20 % $CDCl_3$

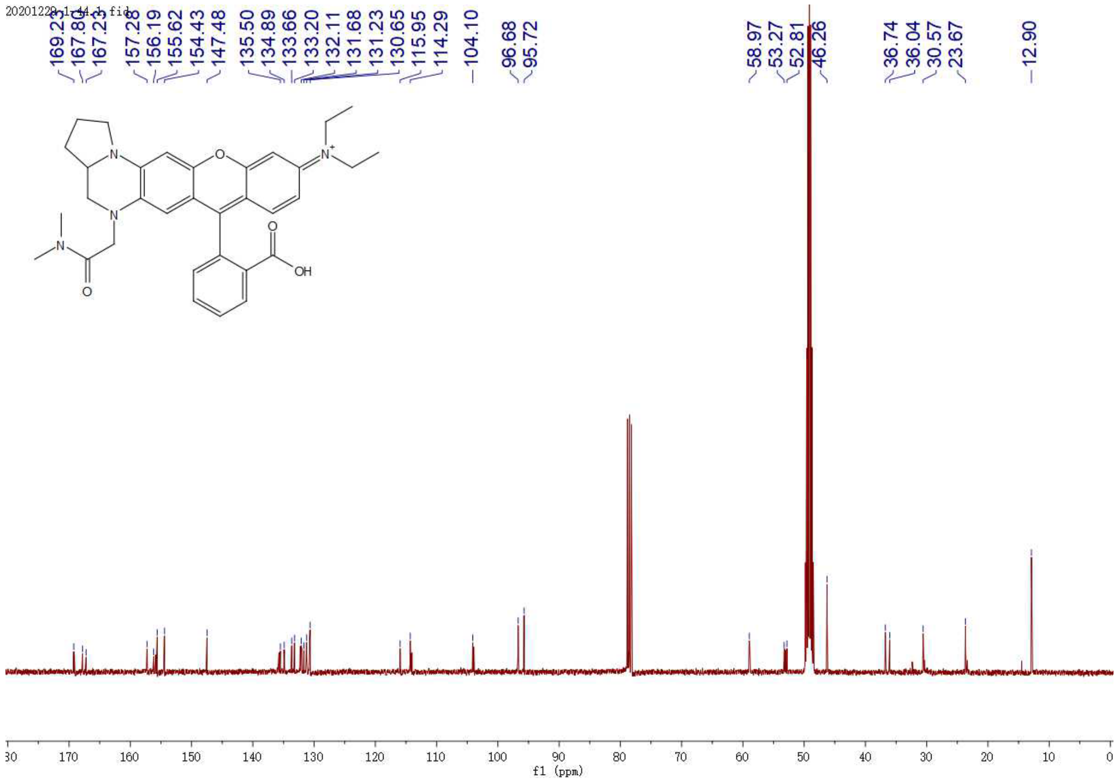

^{13}C NMR spectrum of **3** in CD_3OD containing 20 % $CDCl_3$

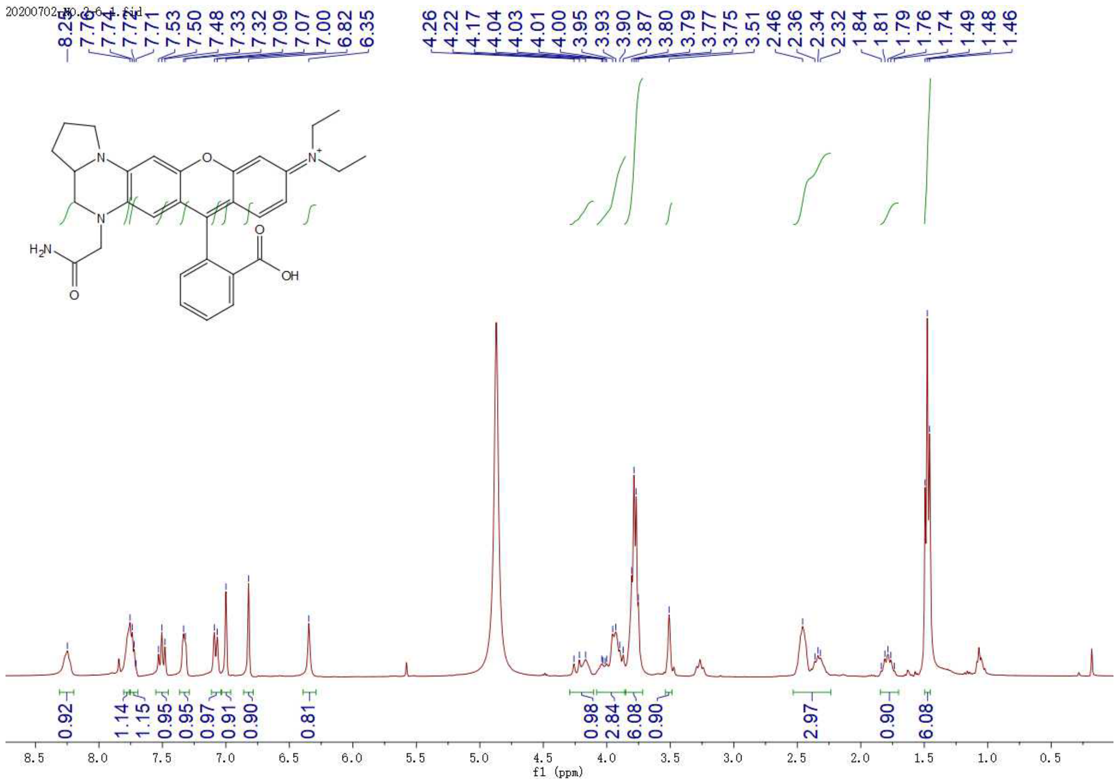

^{1}H NMR spectrum of **4** in CD_3OD containing 20 % $CDCl_3$

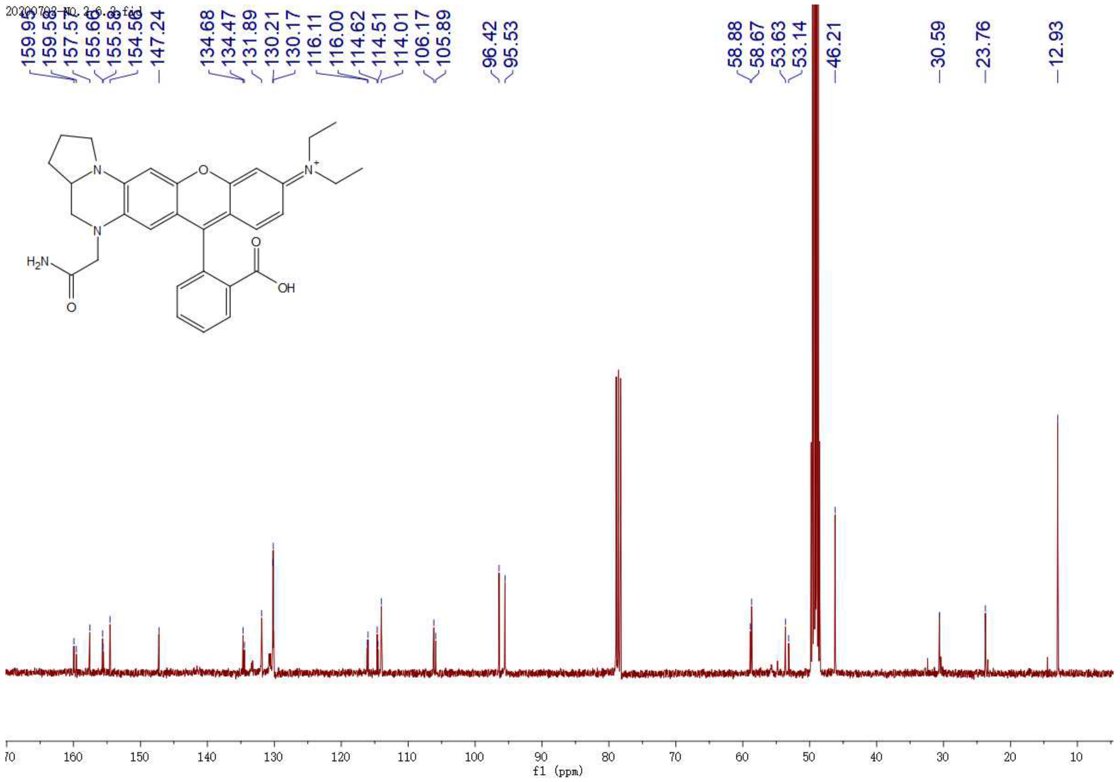

^{13}C NMR spectrum of **4** in CD_3OD containing 20 % $CDCl_3$

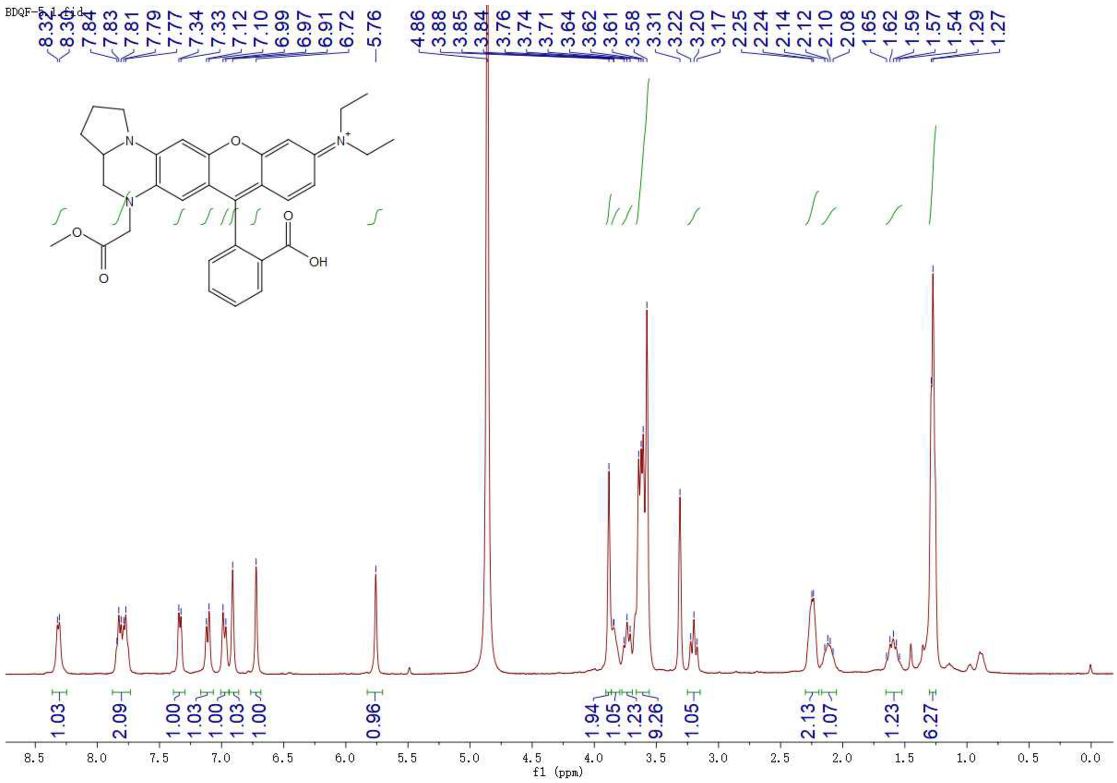

^{1}H NMR spectrum of **5** in CD_3OD

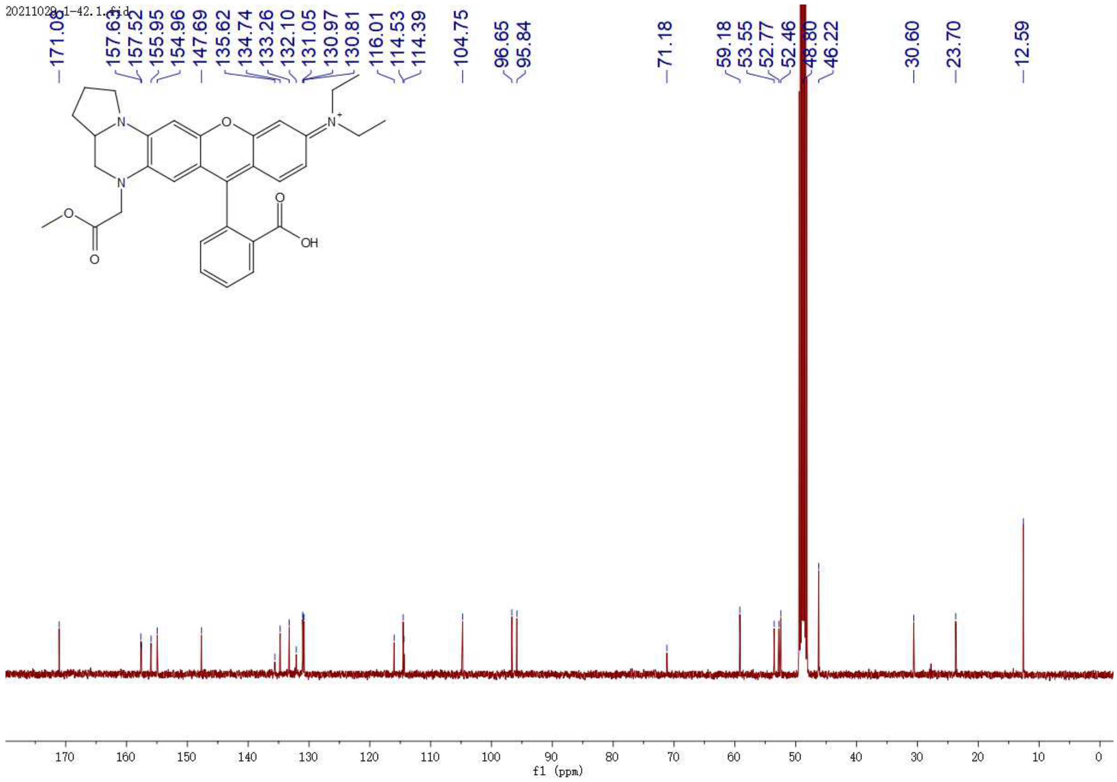

^{13}C NMR spectrum of **5** in CD_3OD

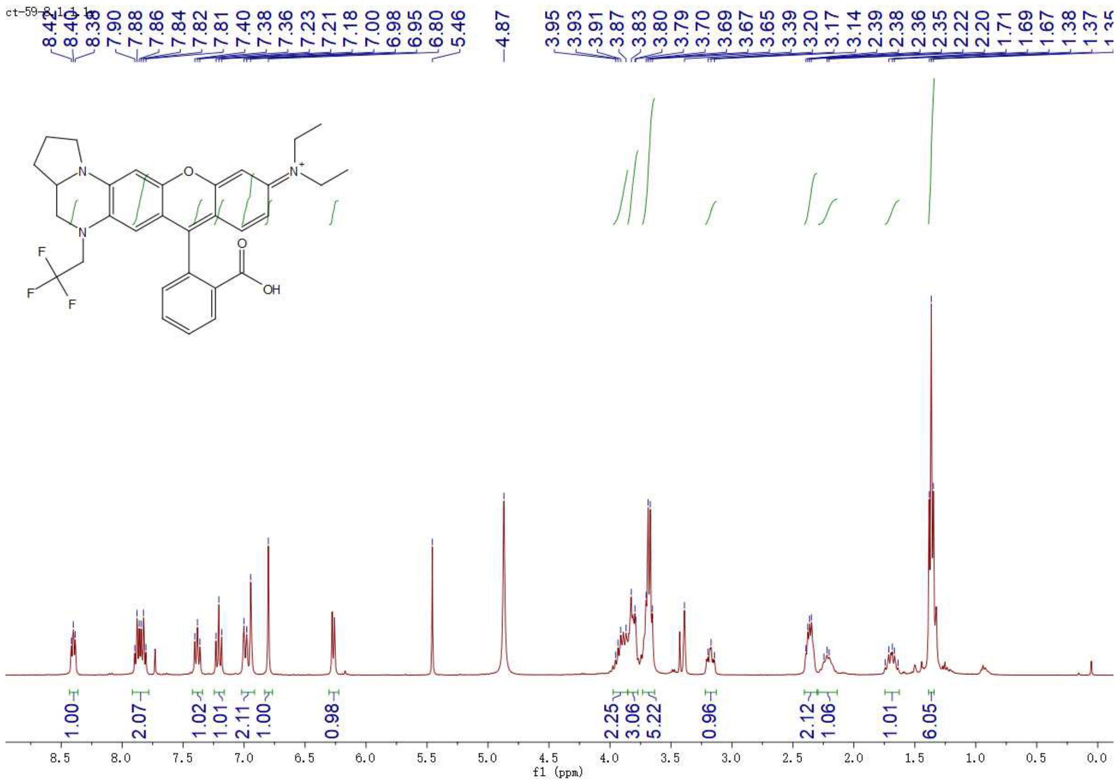

^{1}H NMR spectrum of **6 (YL578)** in CD_3OD containing 20 % $CDCl_3$

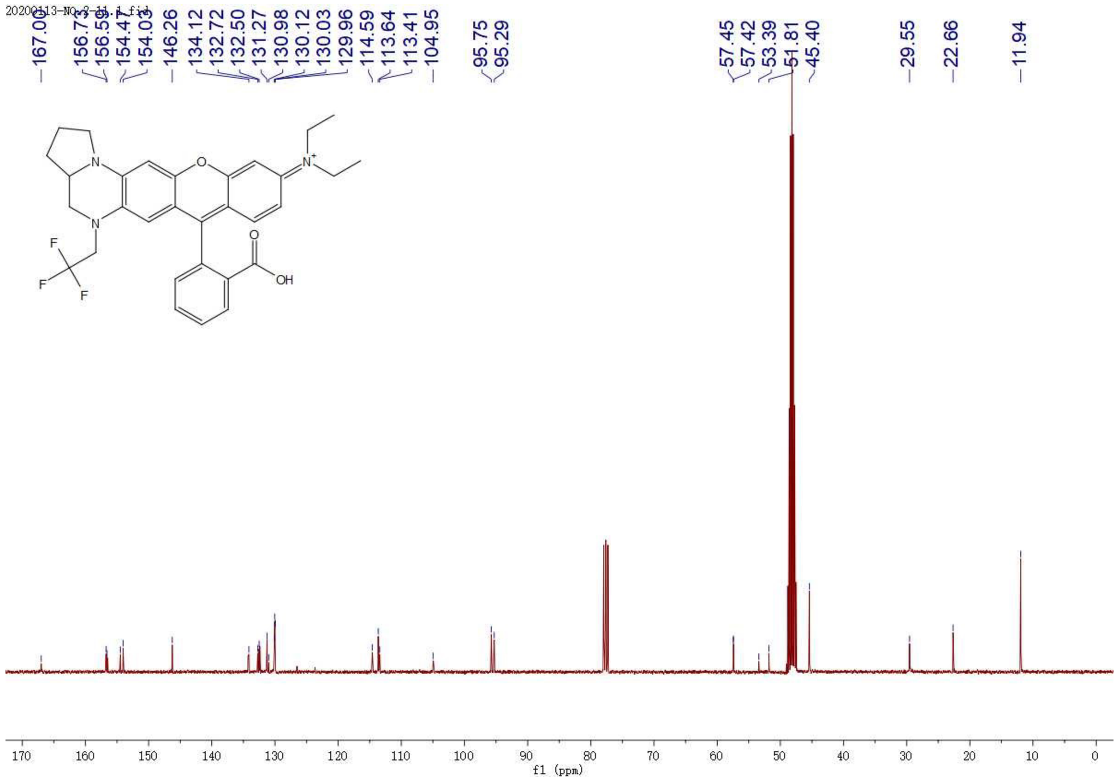

^{13}C NMR spectrum of **6 (YL578)** in CD_3OD containing 20 % $CDCl_3$

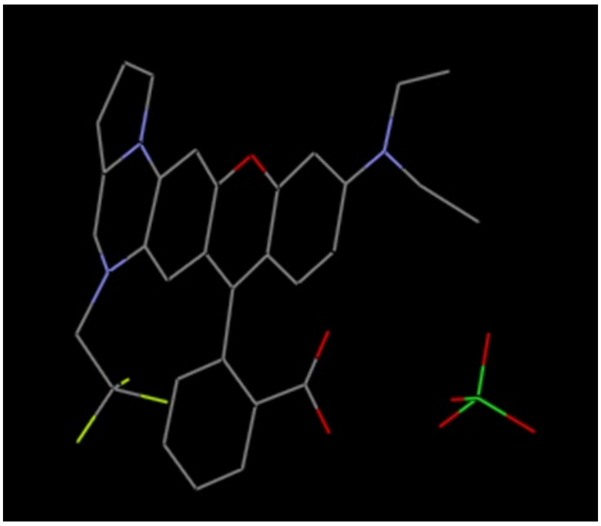

Crystal data of **YL578** (CCDC: 2132329. Yellow, green, red and violet respectively represents F, Cl, O and N atom)

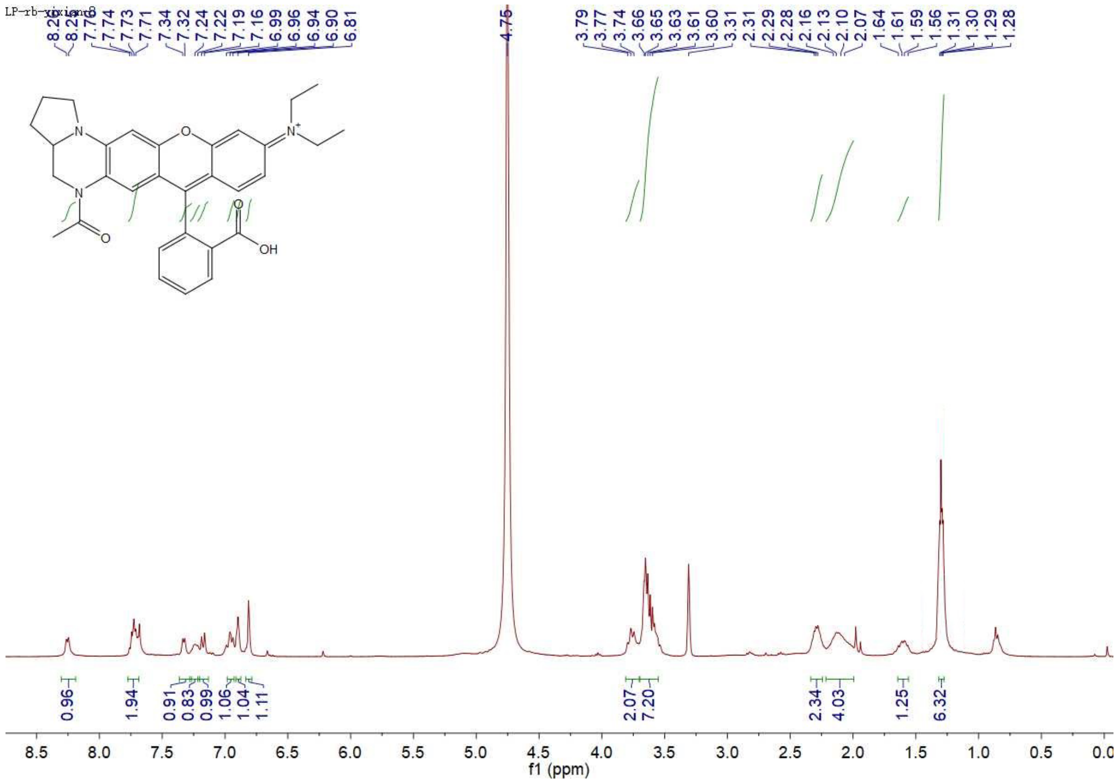

^{1}H NMR spectrum of **7** in CD_3OD

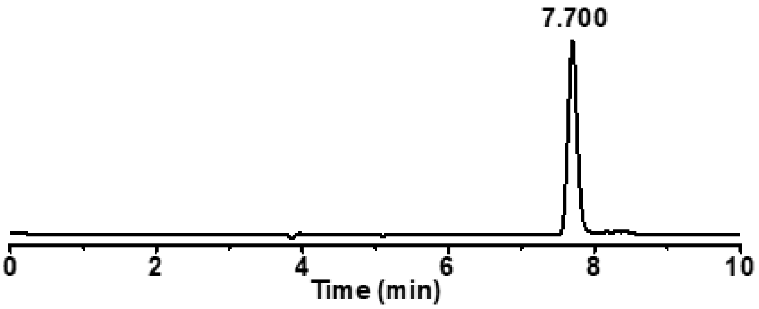

HPLC spectrum of **7**

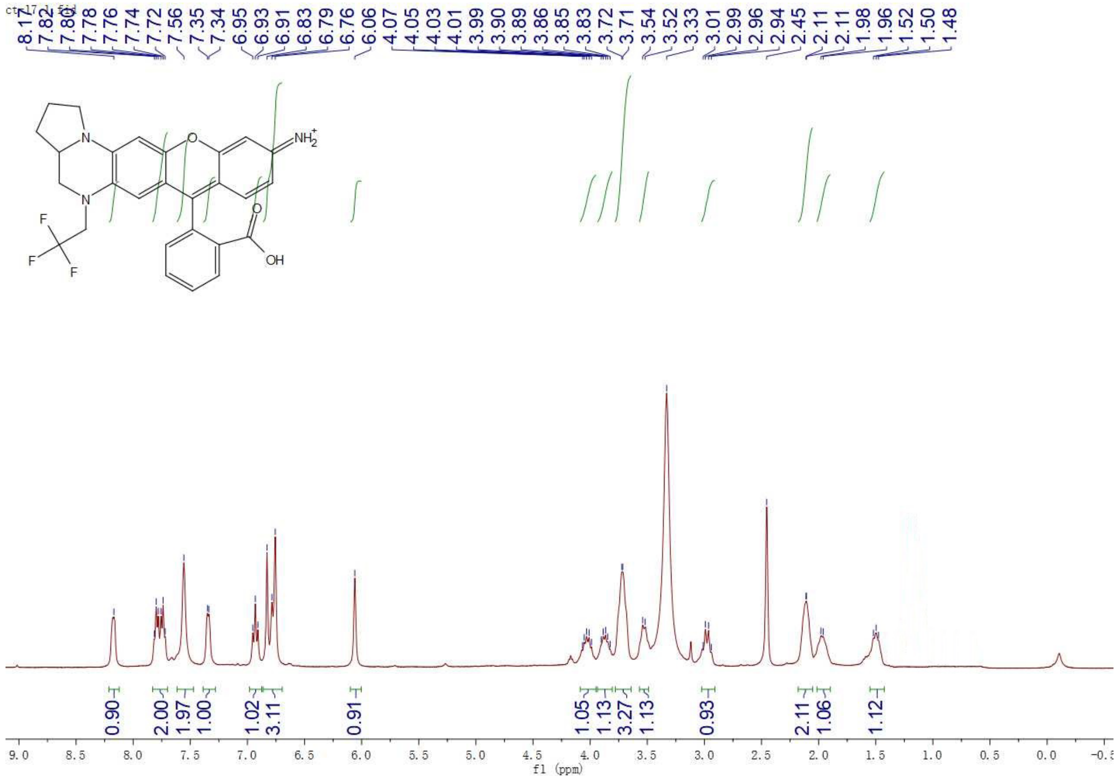

^{1}H NMR spectrum of **8** in DMSO-d_6

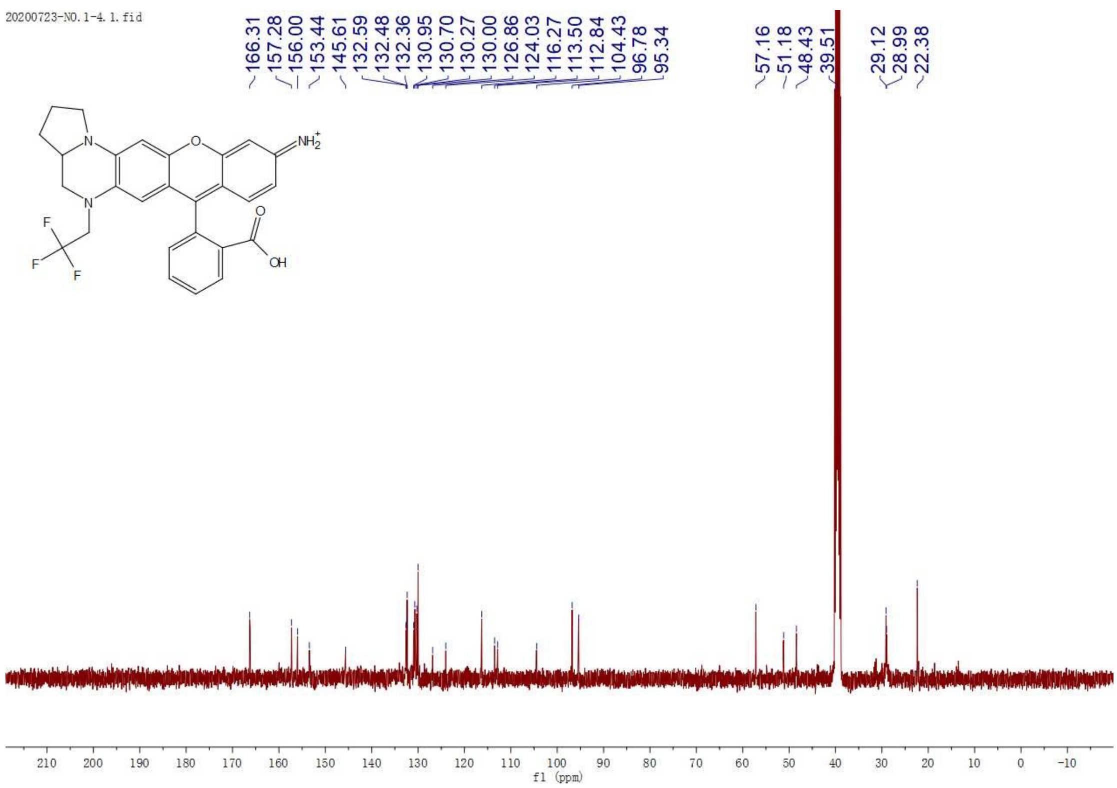

^{13}C NMR spectrum of **8** in DMSO-d$_6$

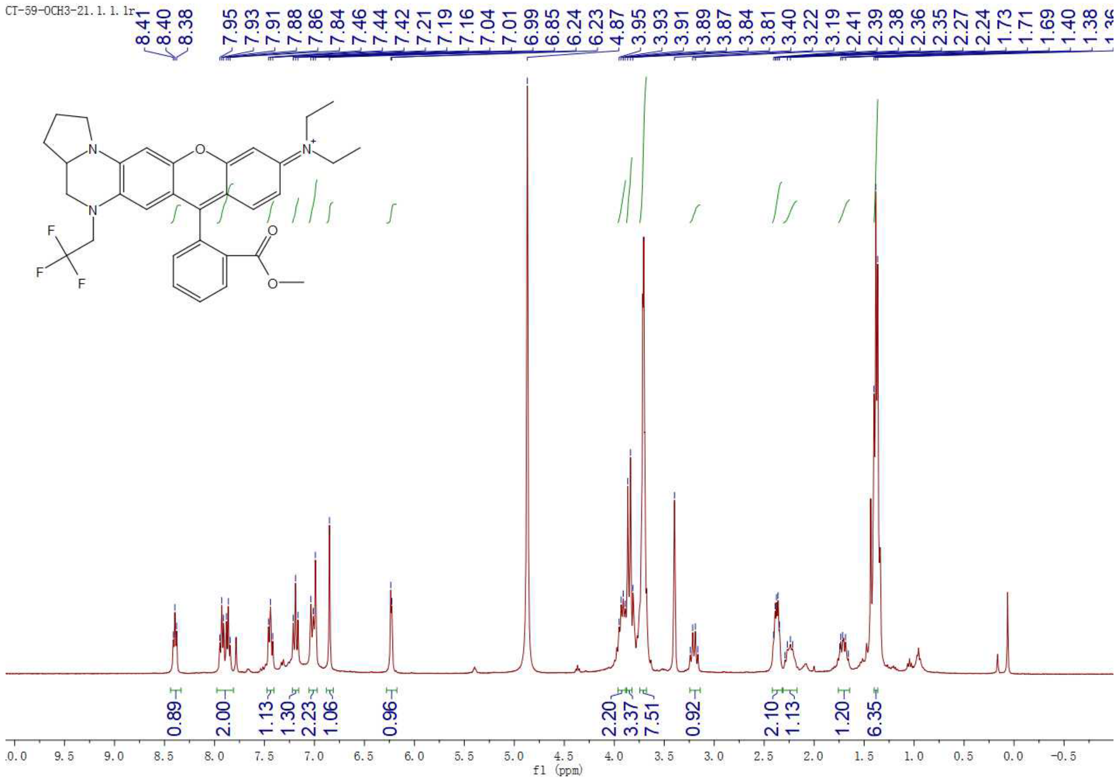

^{1}H NMR spectrum of **YL578-Mito** in CD_3OD containing 20 % $CDCl_3$

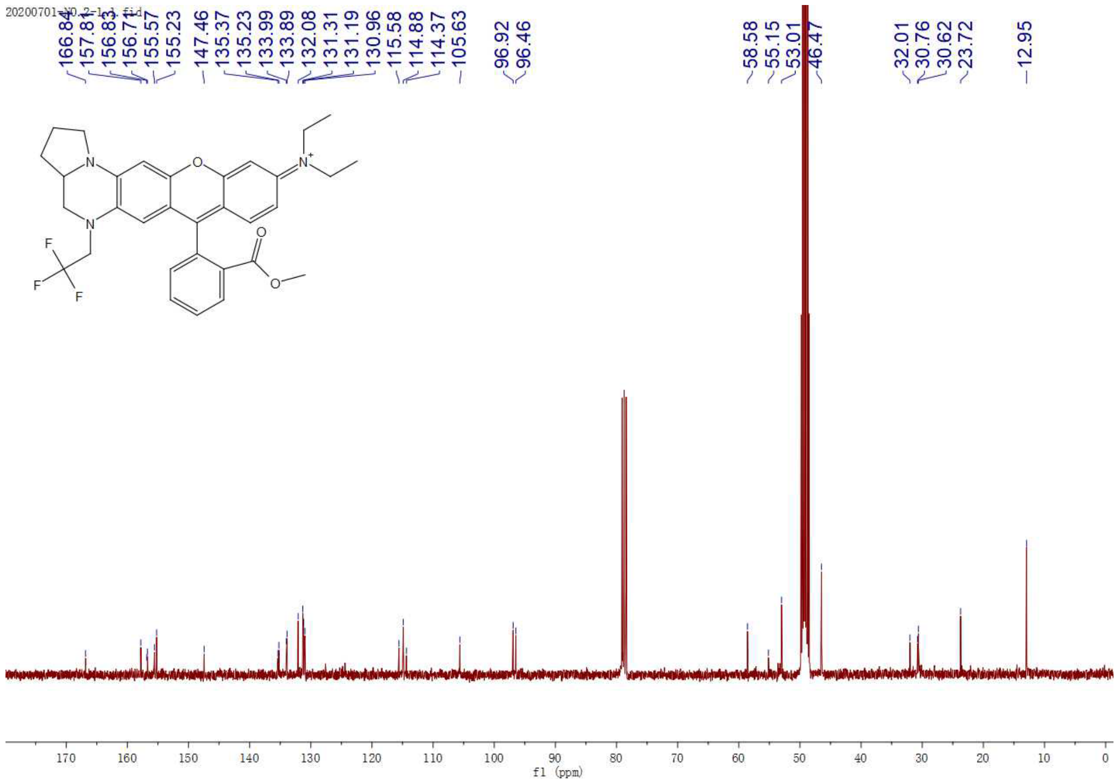

^{13}C NMR spectrum of **YL578-Mito** in CD_3OD containing 20 % $CDCl_3$

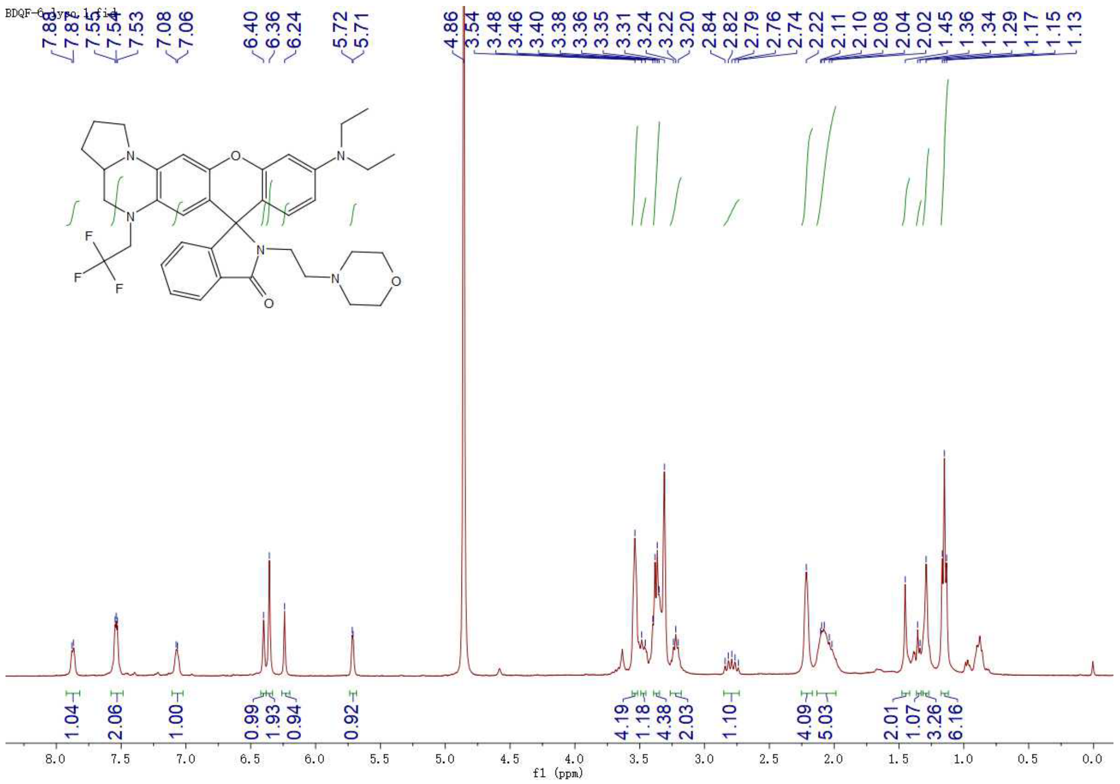

^{1}H NMR spectrum of **YL578-Lyso** in CD_3OD

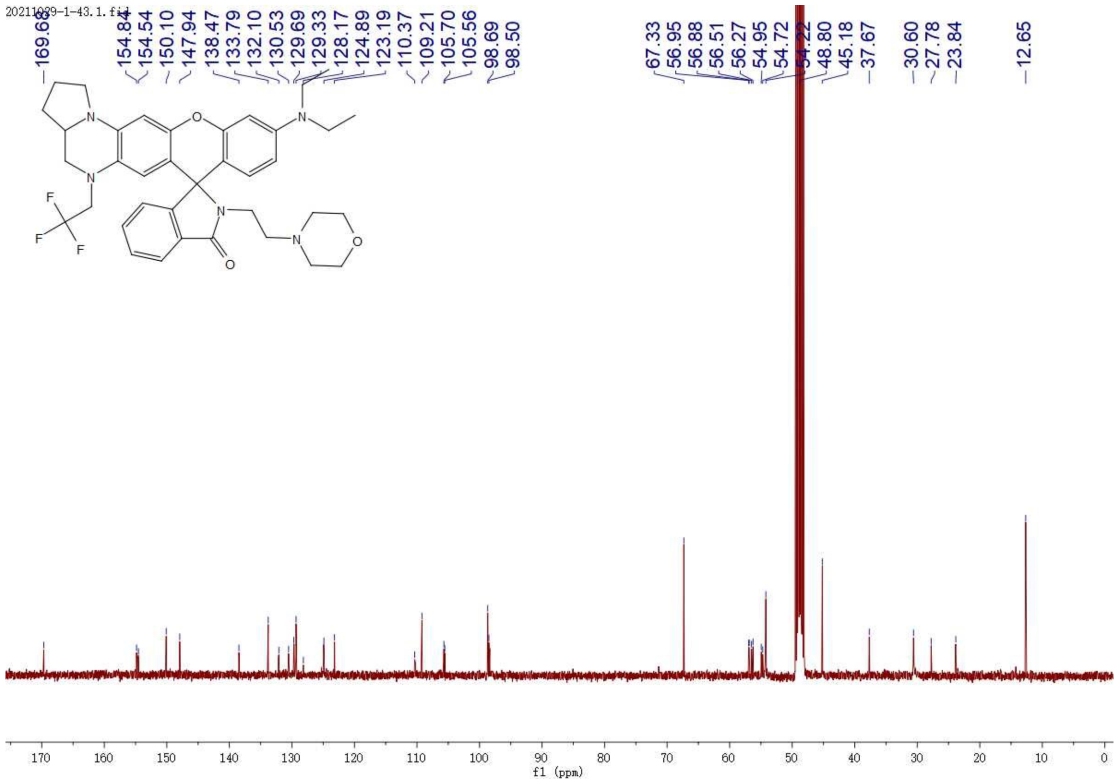

^{13}C NMR spectrum of **YL578-Lyso** in CD_3OD

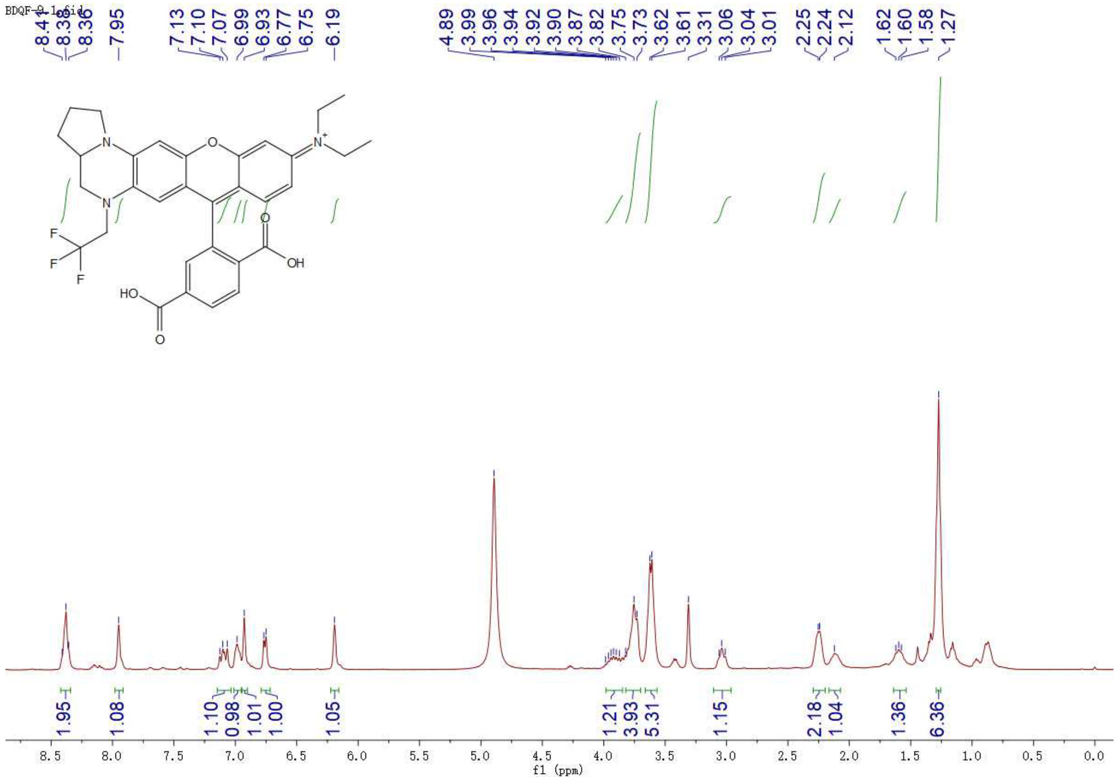

^{1}H NMR spectrum of **9** in CD_3OD

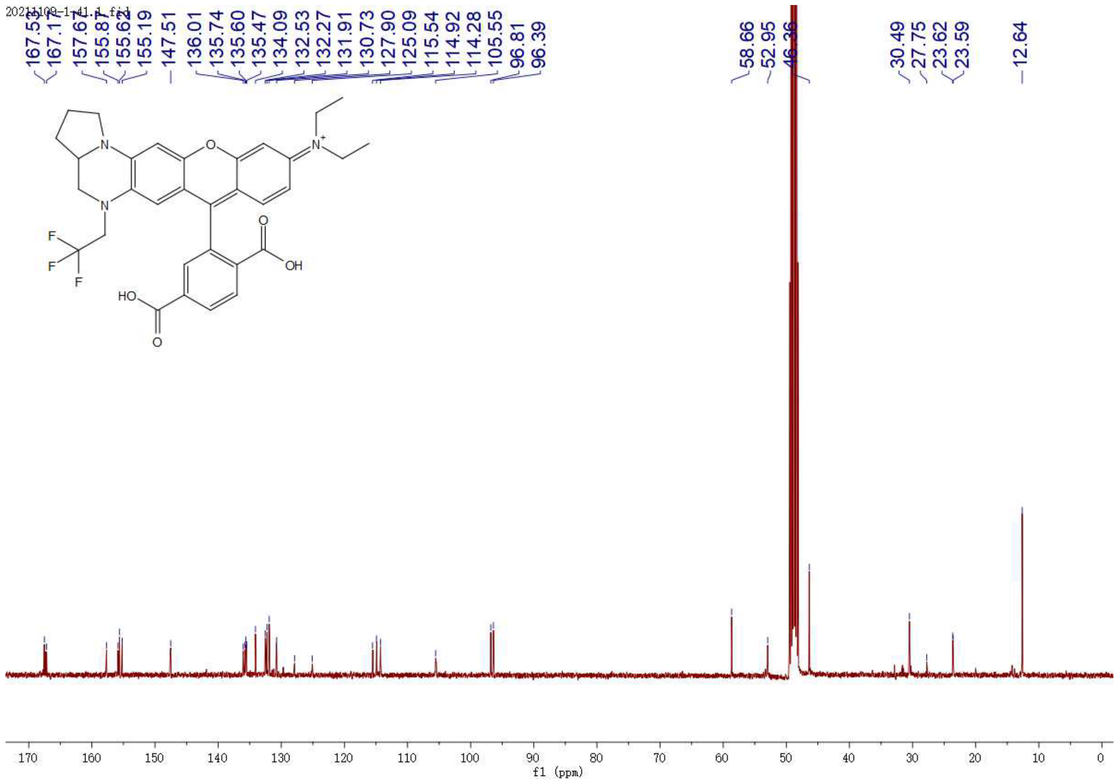

^{13}C NMR spectrum of **9** in CD_3OD

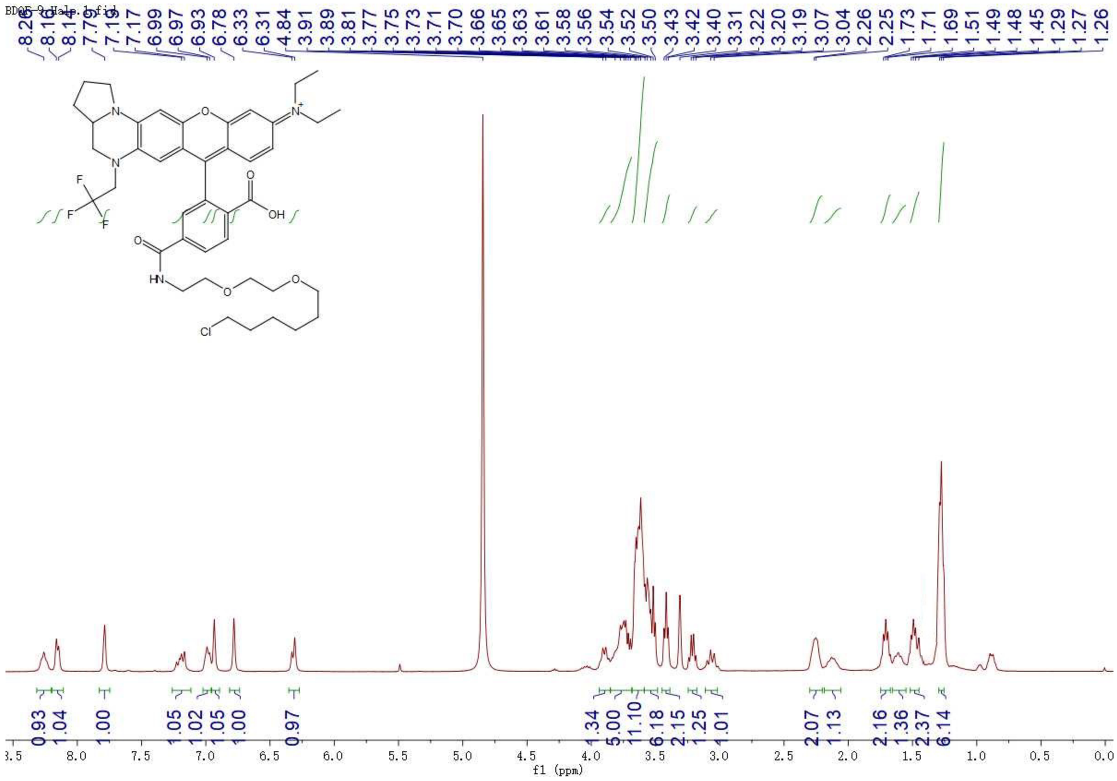

^{1}H NMR spectrum of **YL578-Halo** in CD_3OD.

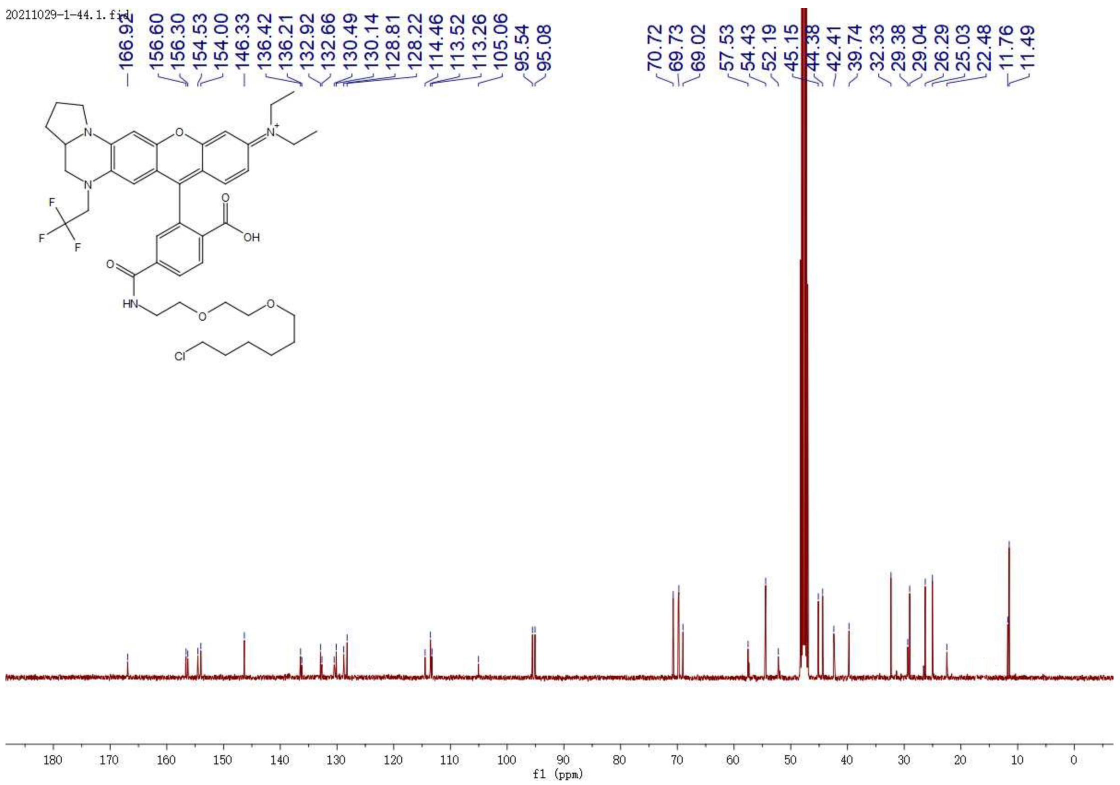

^{13}C NMR spectrum of **YL578-Halo** in CD_3OD.

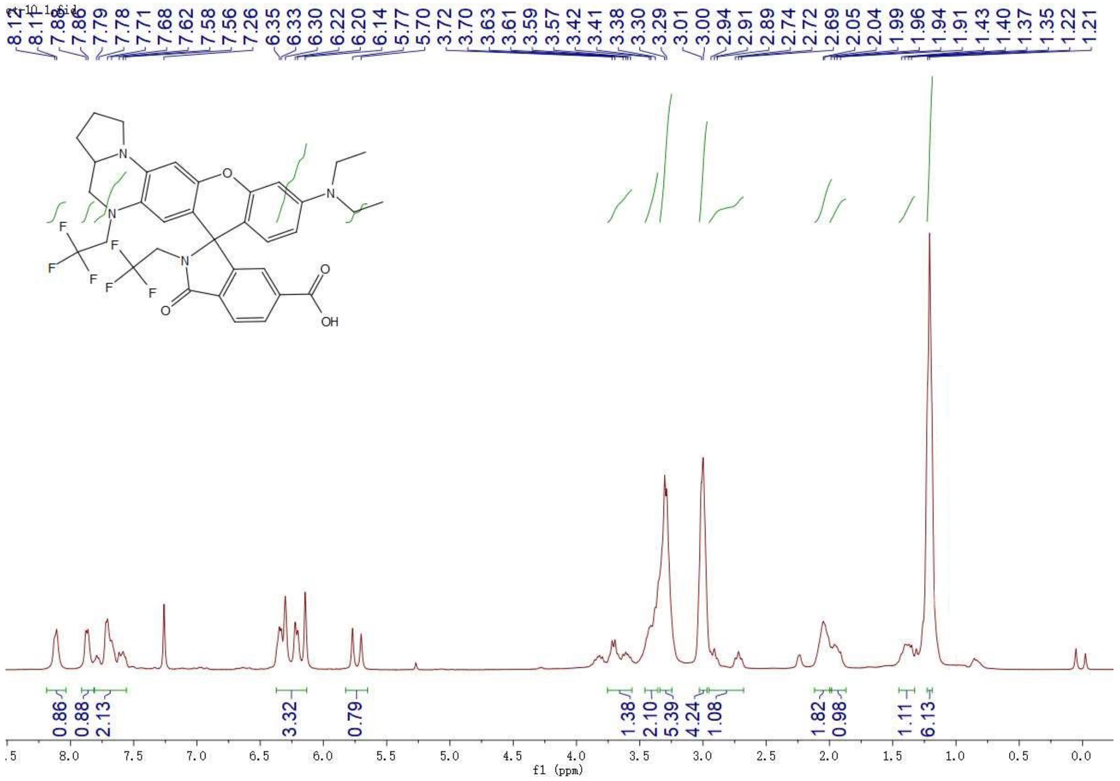

^{1}H NMR spectrum of **10** in $CDCl_3$.

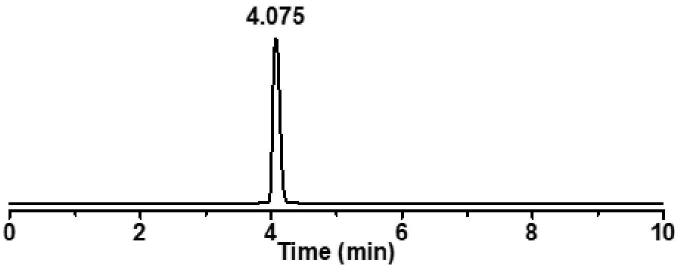

HPLC spectrum of **10**

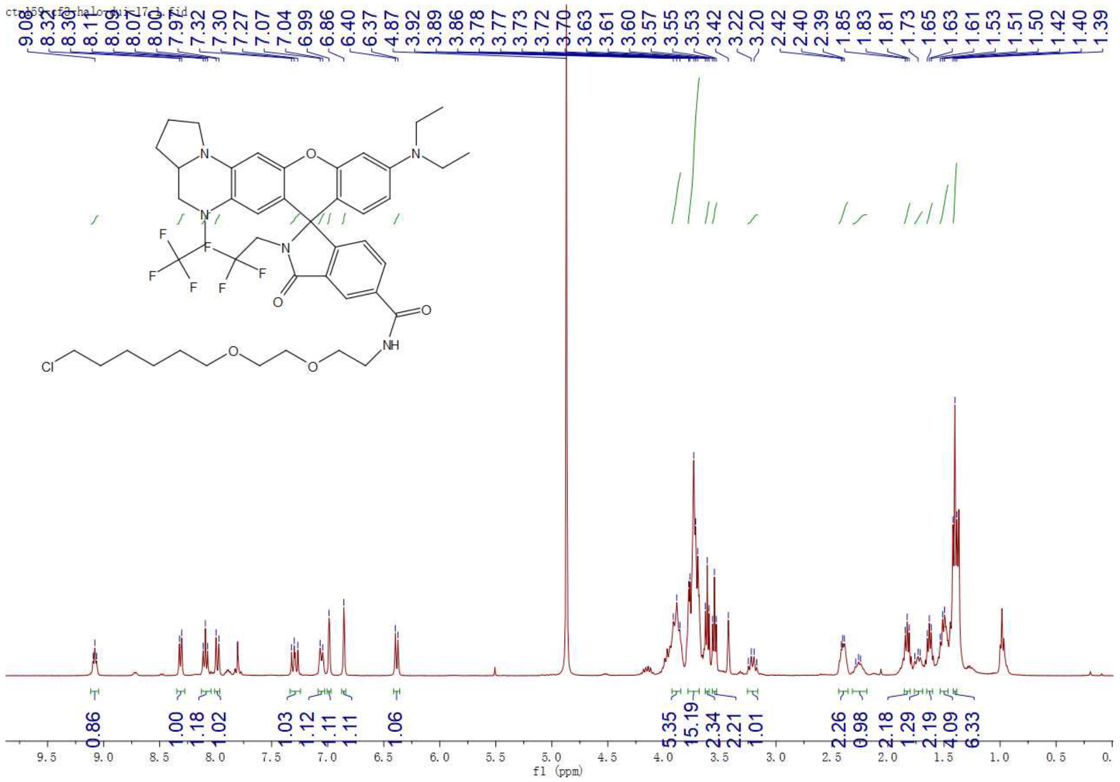

^{1}H NMR spectrum of **10-Halo** in CD_3OD containing 20 % $CDCl_3$

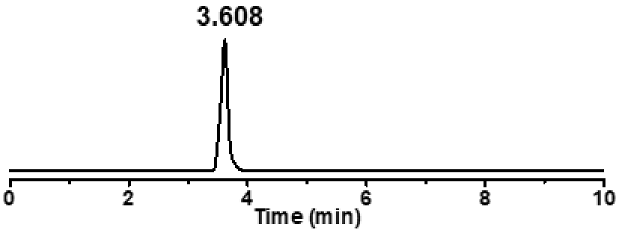

HPLC spectrum of **10-Halo**

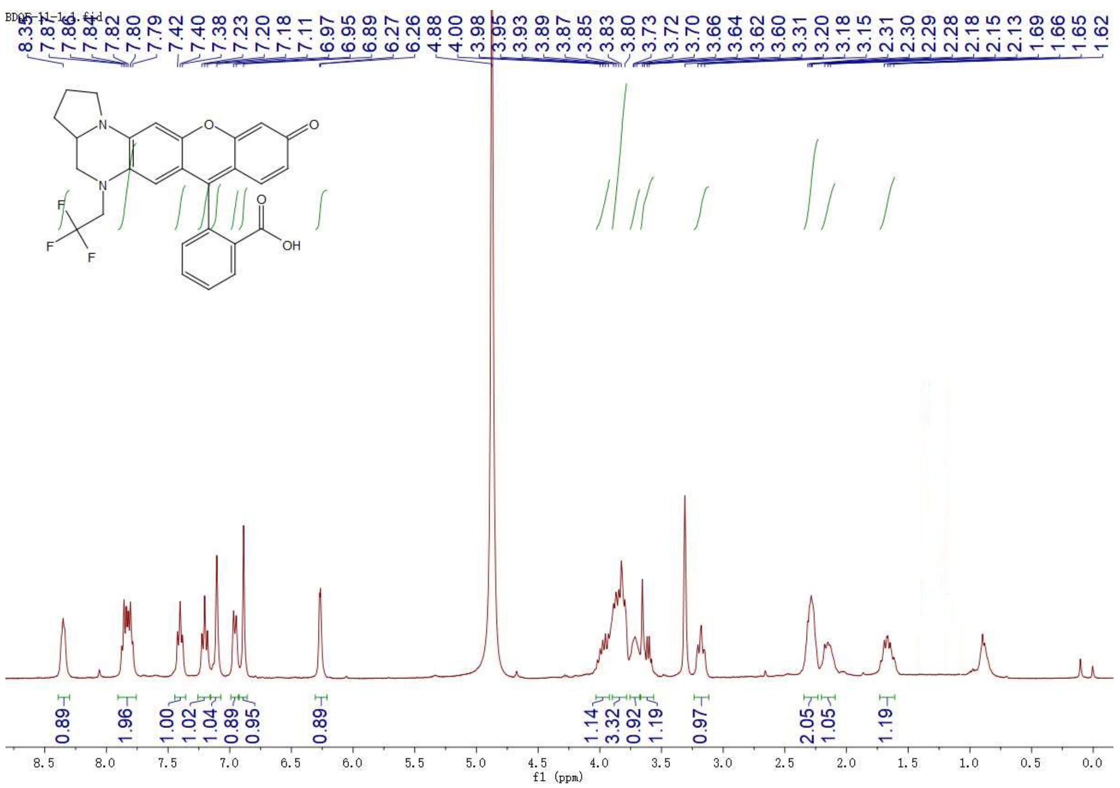

^{1}H NMR spectrum of **11** in CD_3OD containing 20 % $CDCl_3$

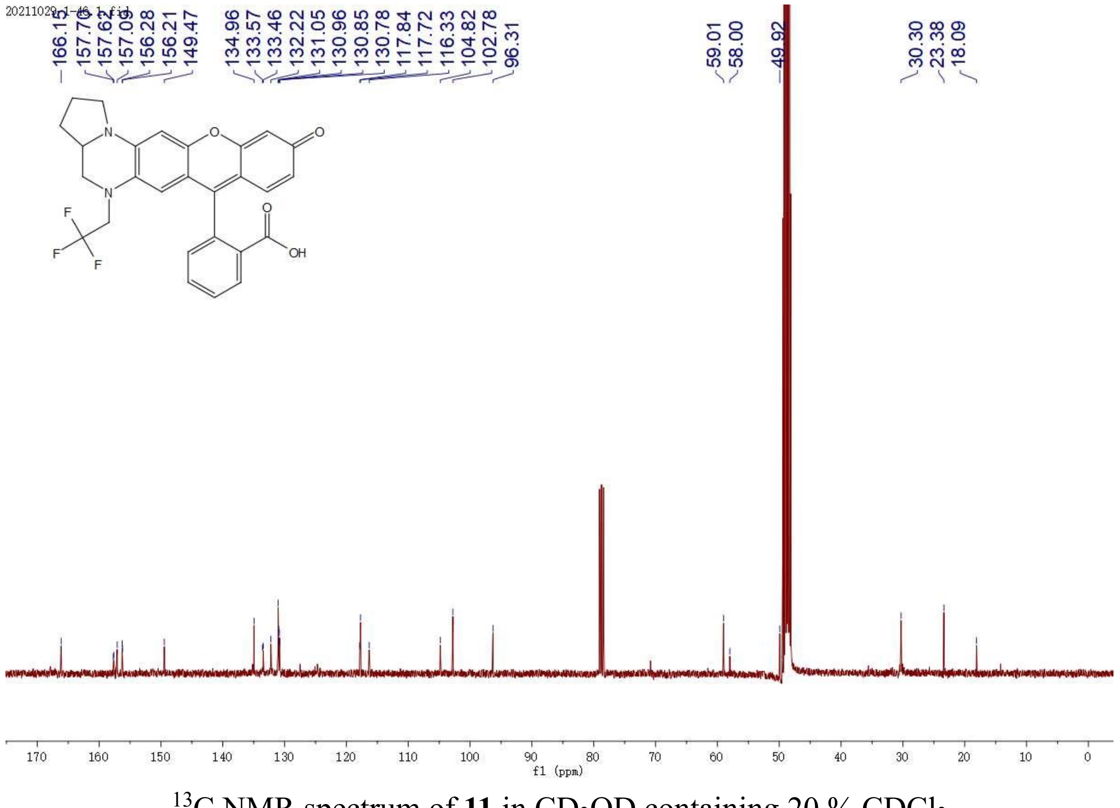

^{13}C NMR spectrum of **11** in CD_3OD containing 20 % $CDCl_3$

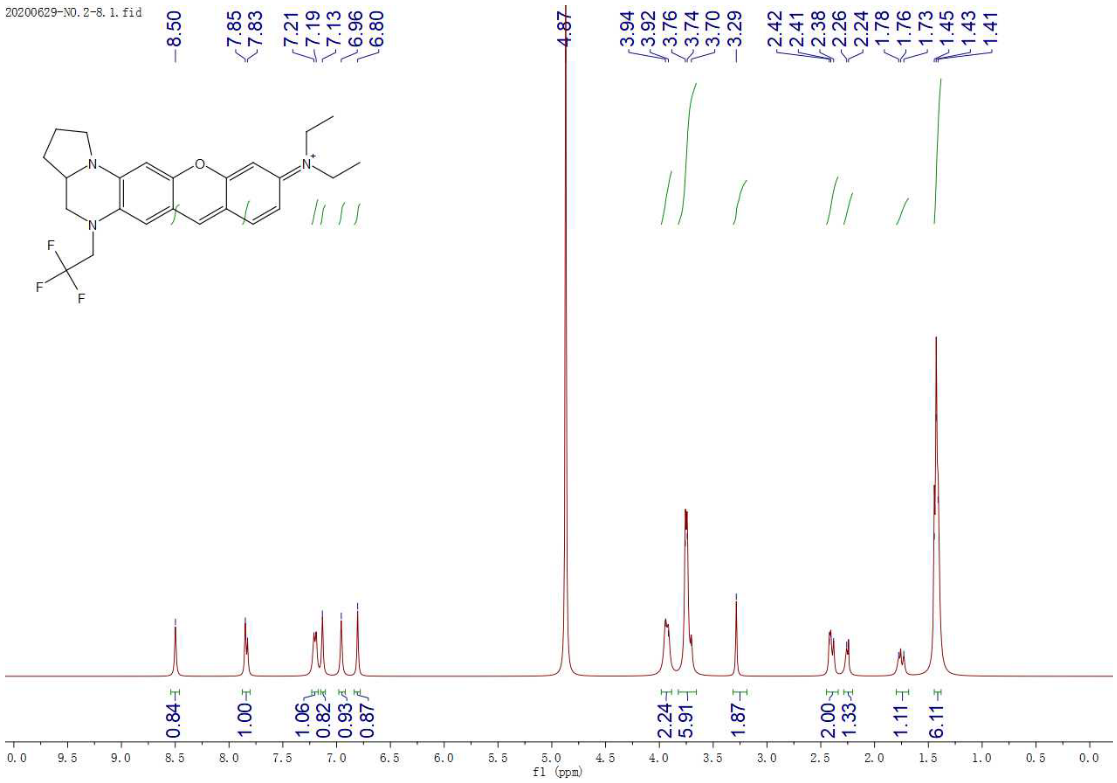

^{1}H NMR spectrum of **12** in CD_3OD containing 20 % $CDCl_3$

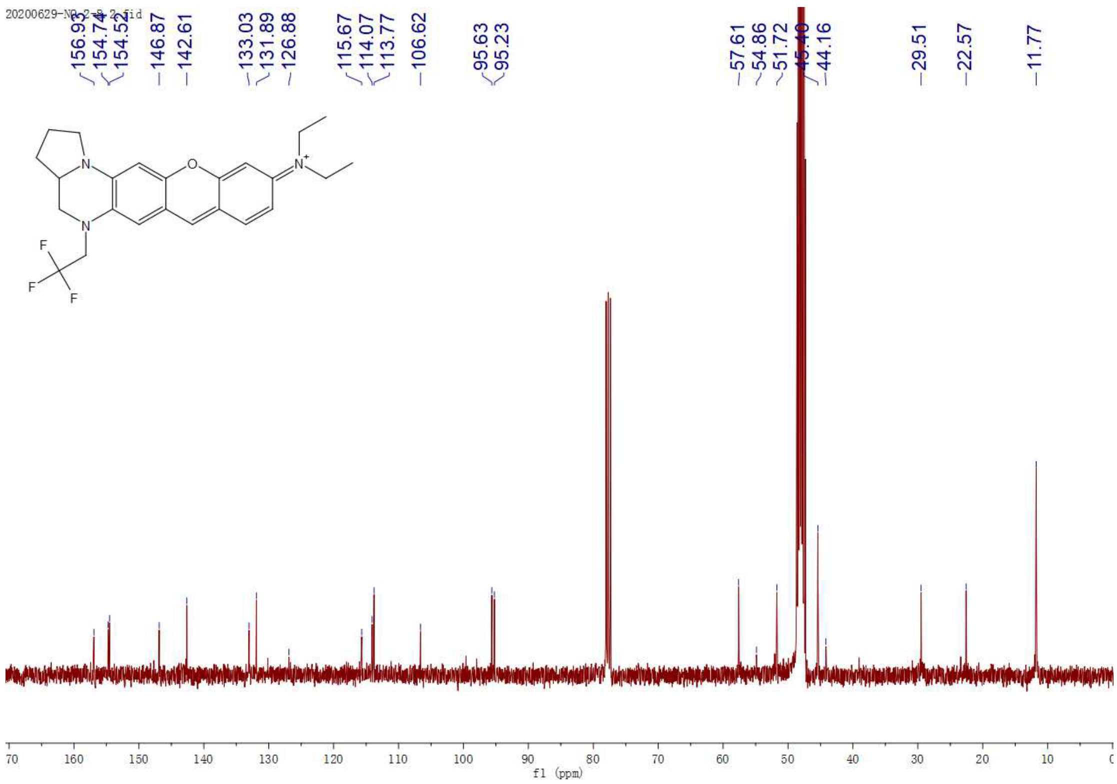

^{13}C NMR spectrum of **12** in CD_3OD containing 20 % $CDCl_3$

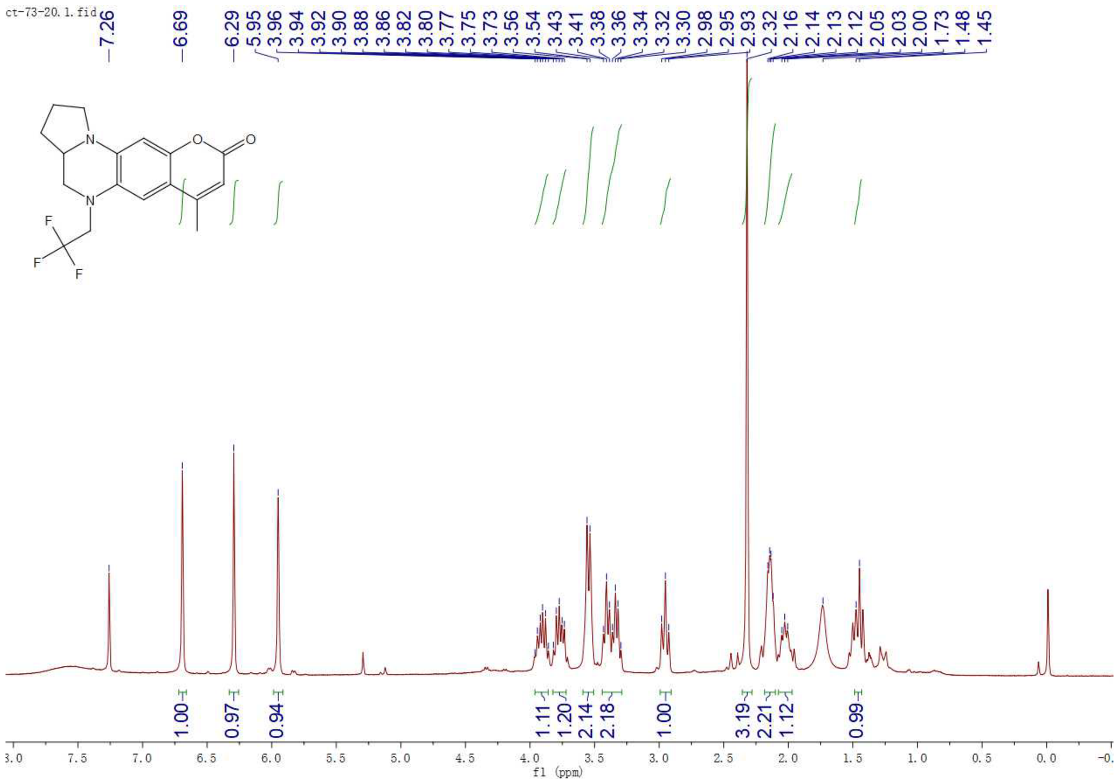

^{1}H NMR spectrum of **13** in $CDCl_3$

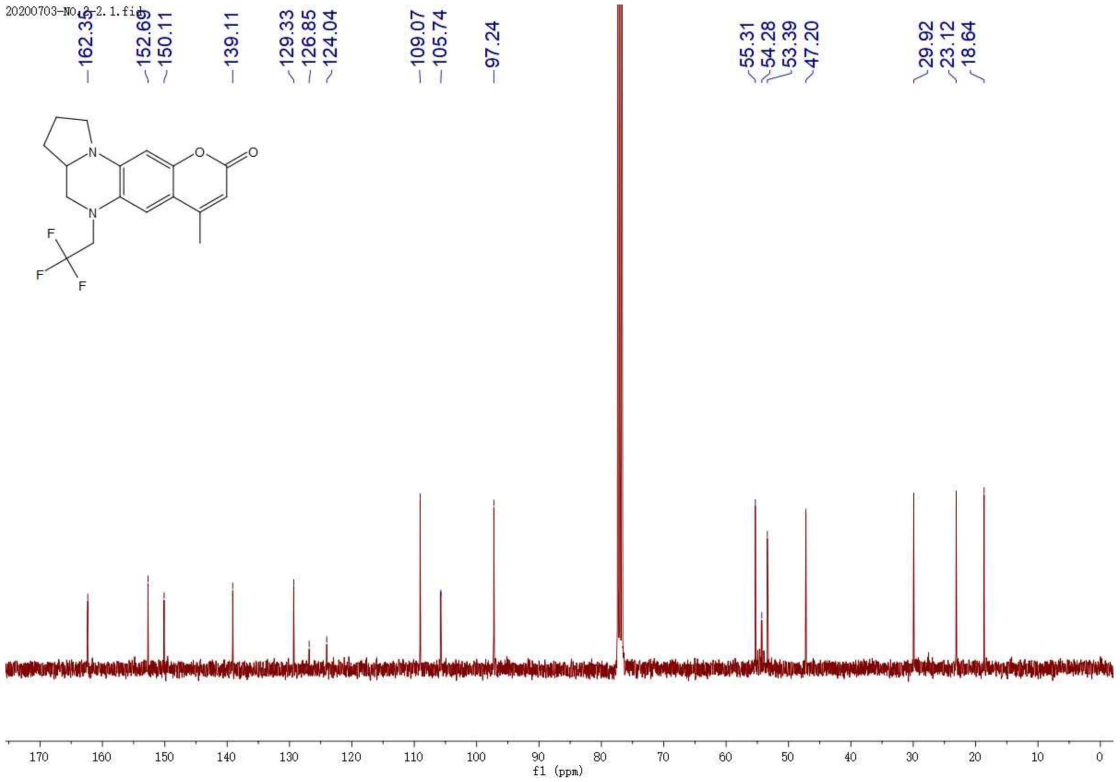

^{13}C NMR spectrum of **13** in $CDCl_3$

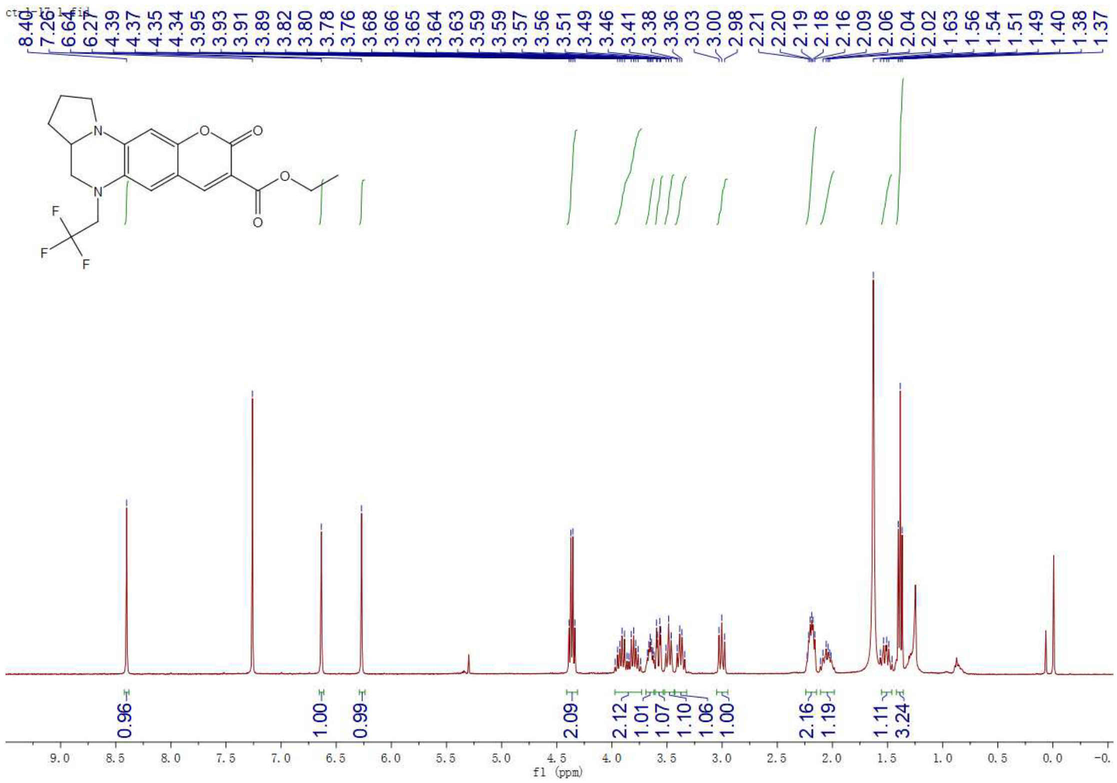

^{1}H NMR spectrum of **14** in $CDCl_3$

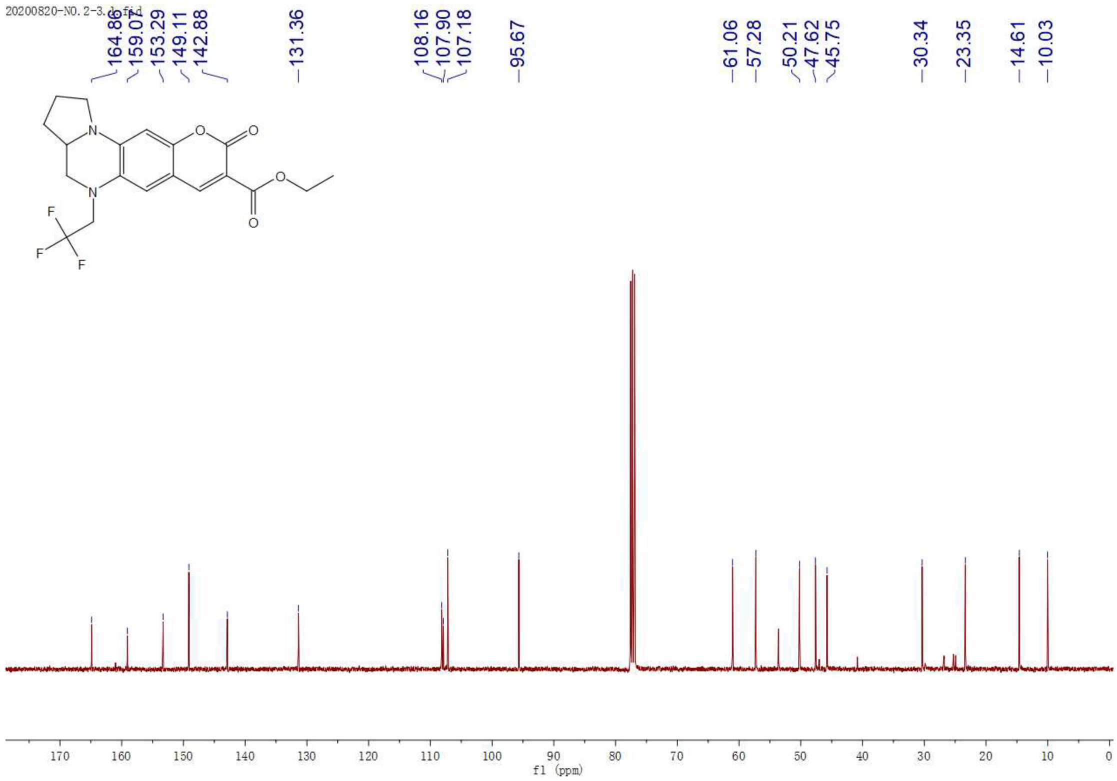

^{13}C NMR spectrum of **14** in $CDCl_3$

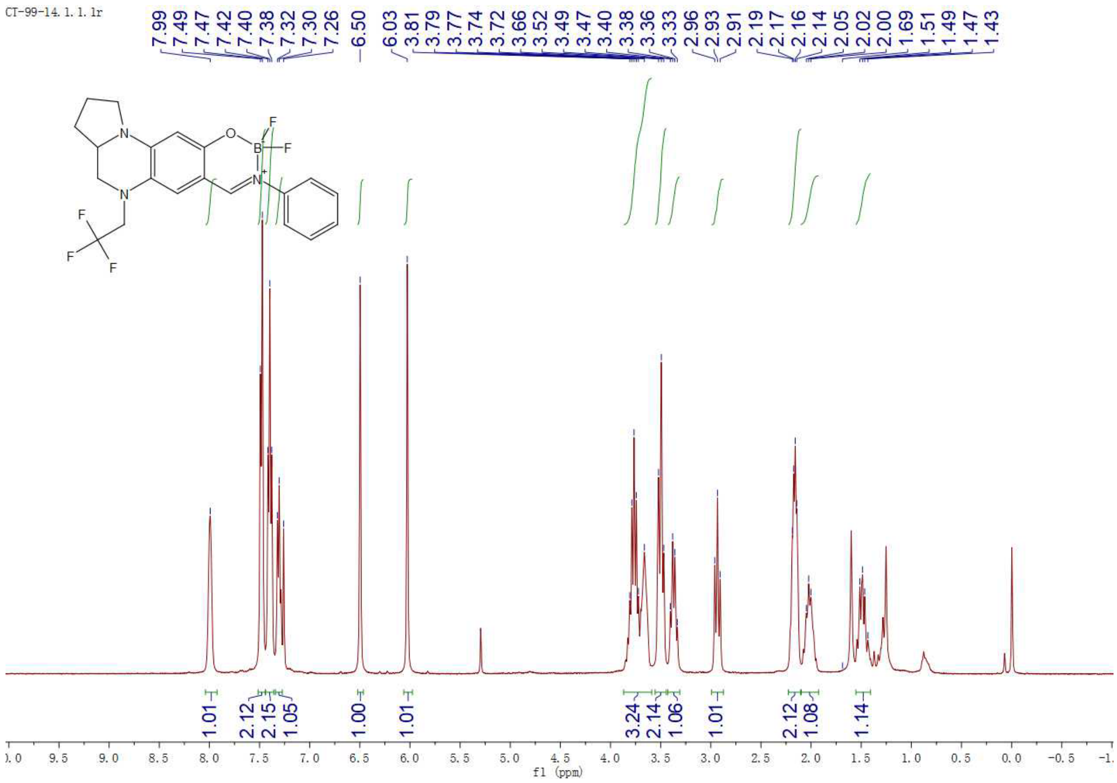

^{1}H NMR spectrum of **15** in $CDCl_3$

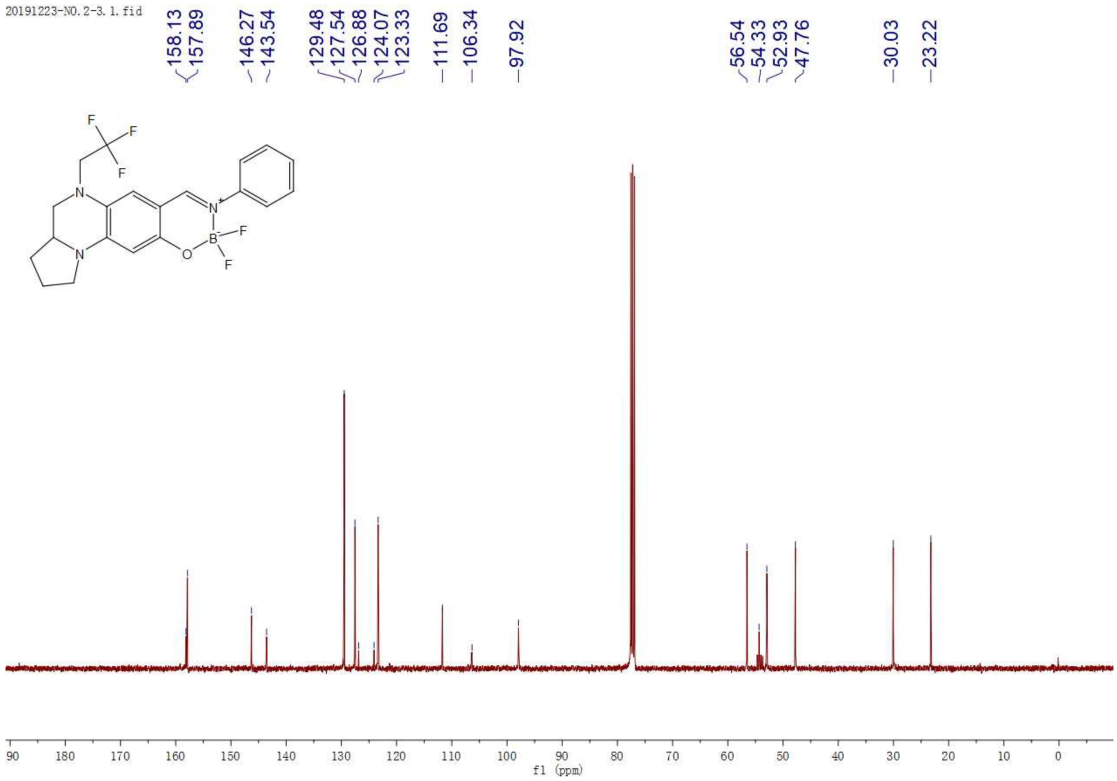

^{13}C NMR spectrum of **15** in $CDCl_3$

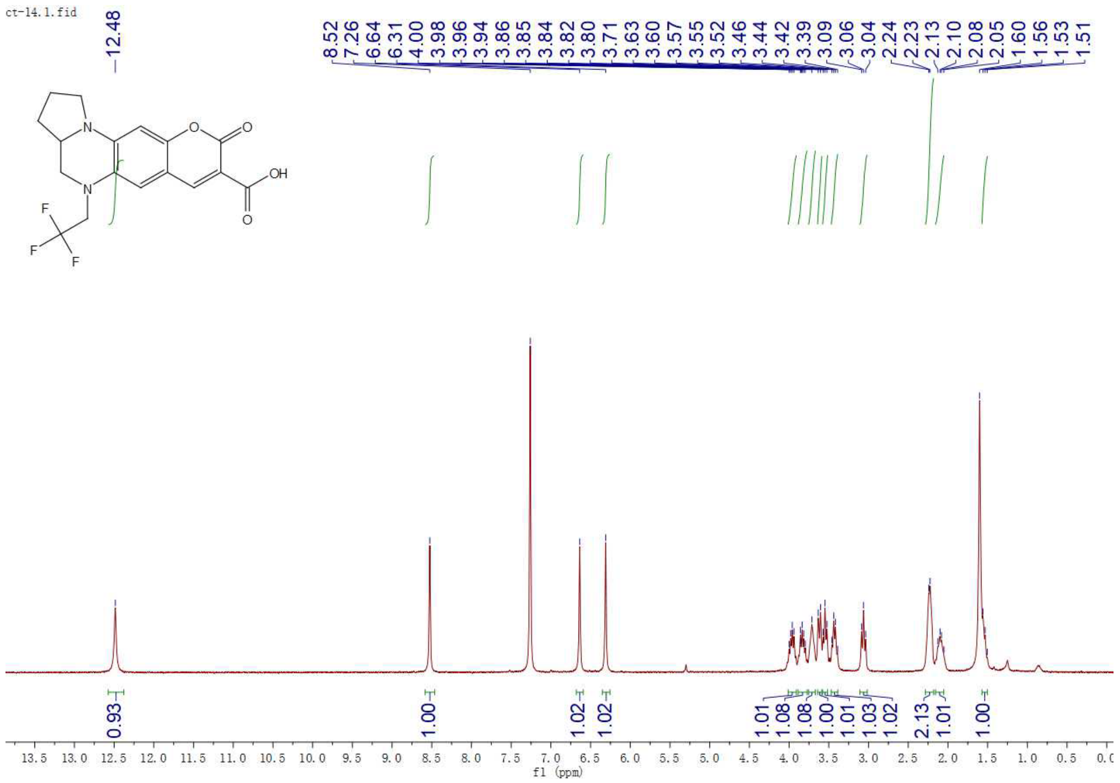

^{1}H NMR spectrum of **16** in $CDCl_3$

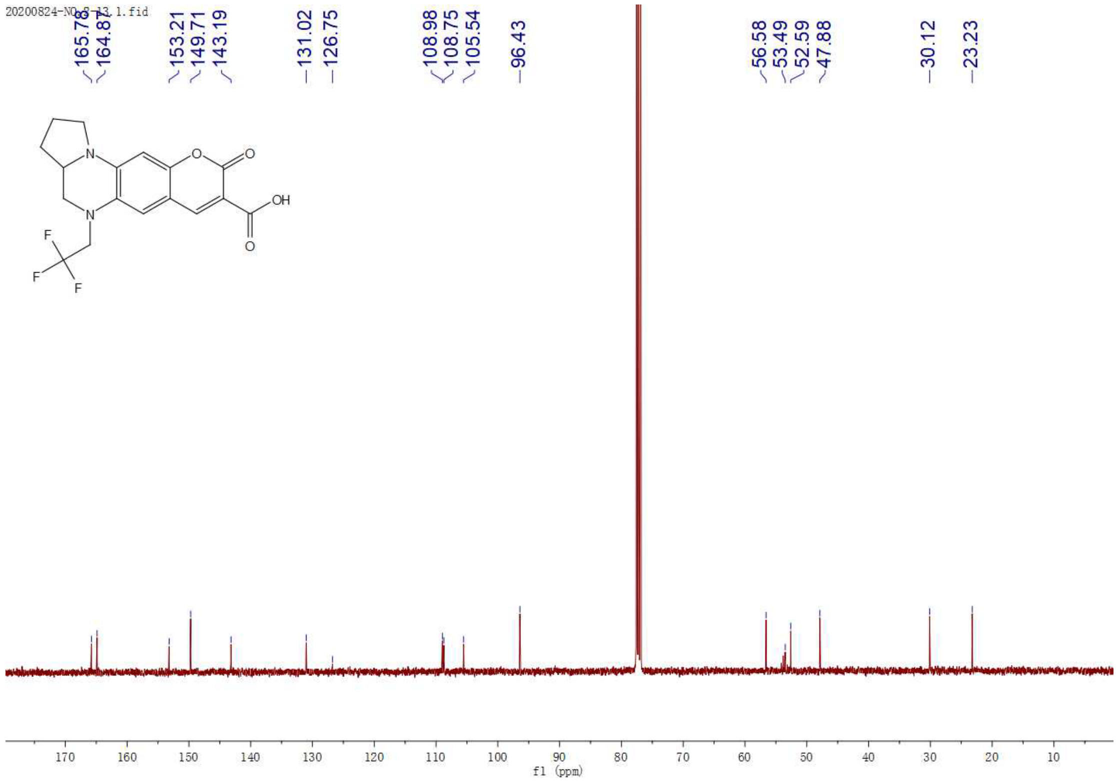

^{13}C NMR spectrum of **16** in $CDCl_3$

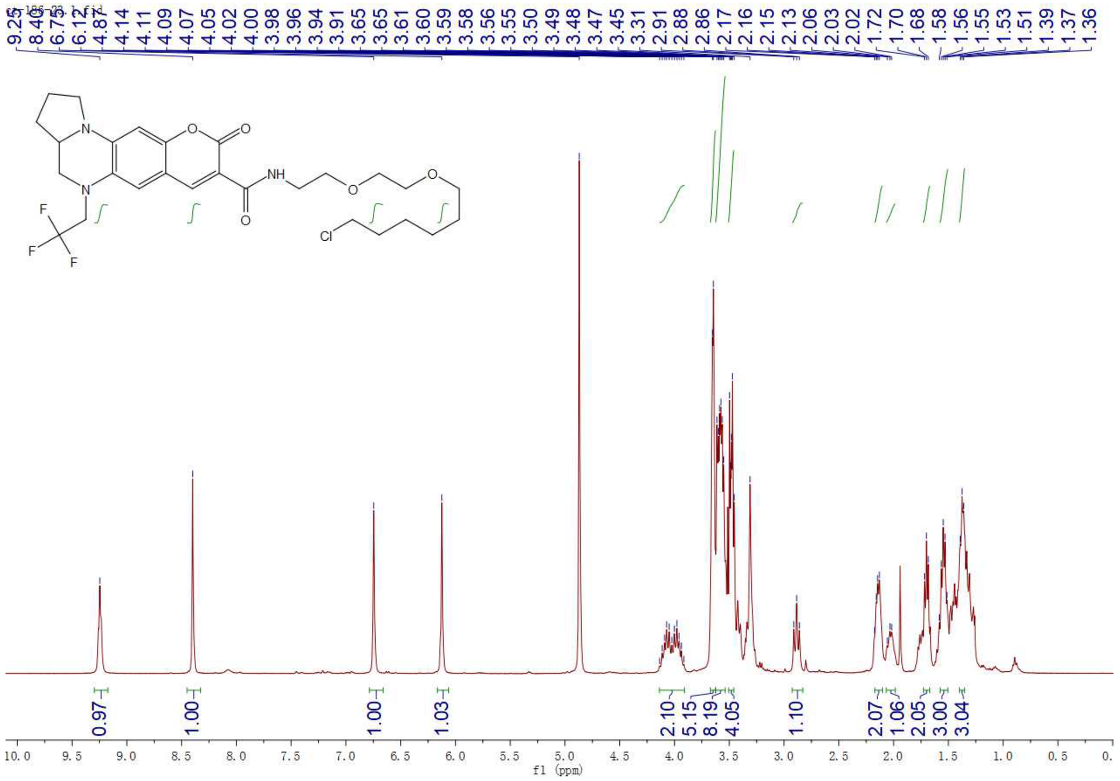

^{1}H NMR spectrum of **16-Halo** in CD_3OD

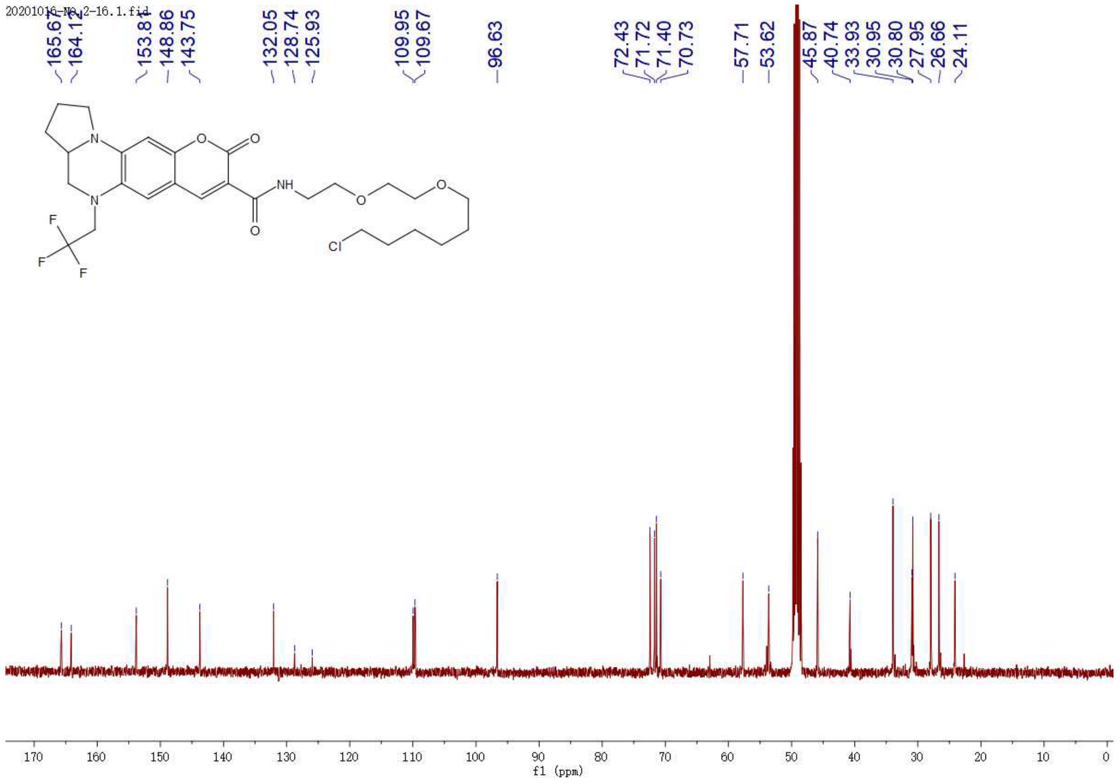

^{13}C NMR spectrum of **16-Halo** in CD_3OD

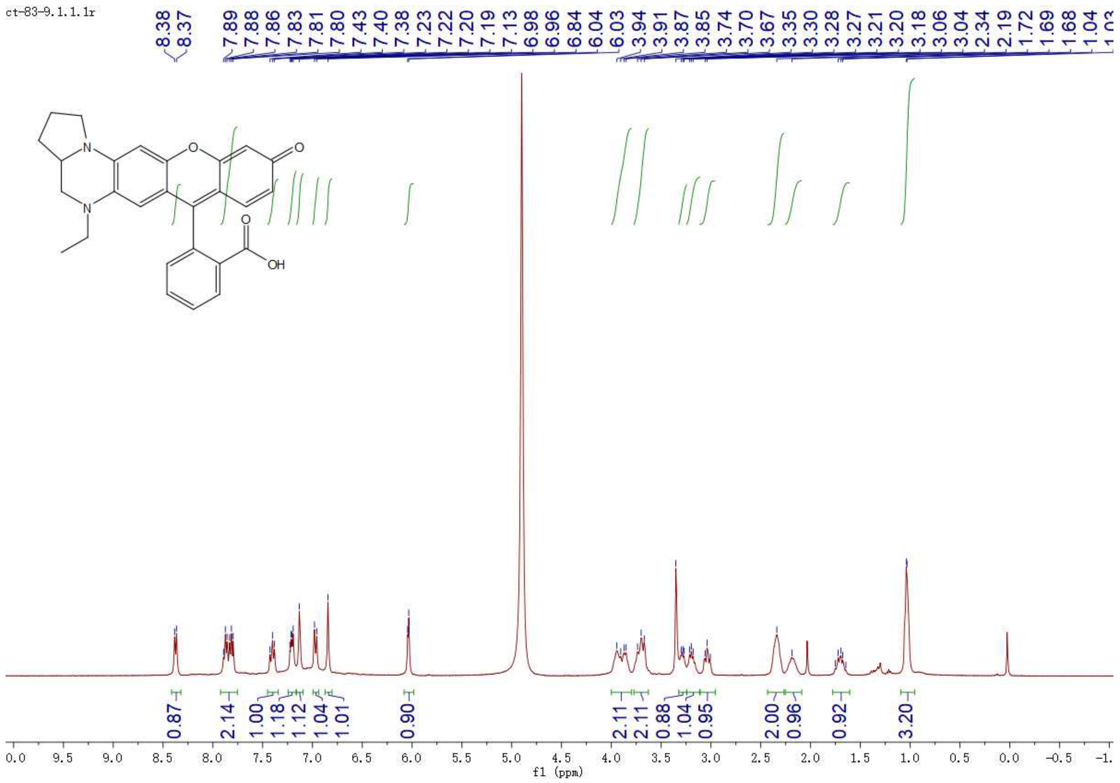

^{1}H NMR spectrum of **17** in CD_3OD containing 20 % $CDCl_3$

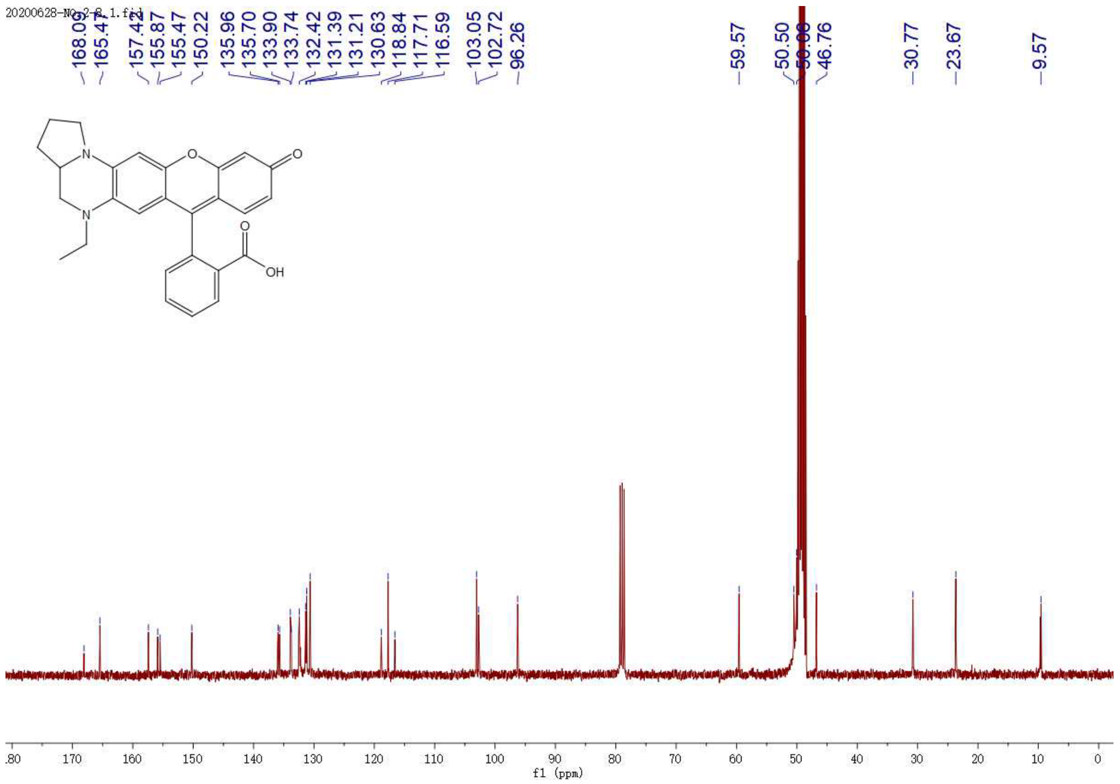

^{13}C NMR spectrum of **17** in CD_3OD containing 20 % $CDCl_3$

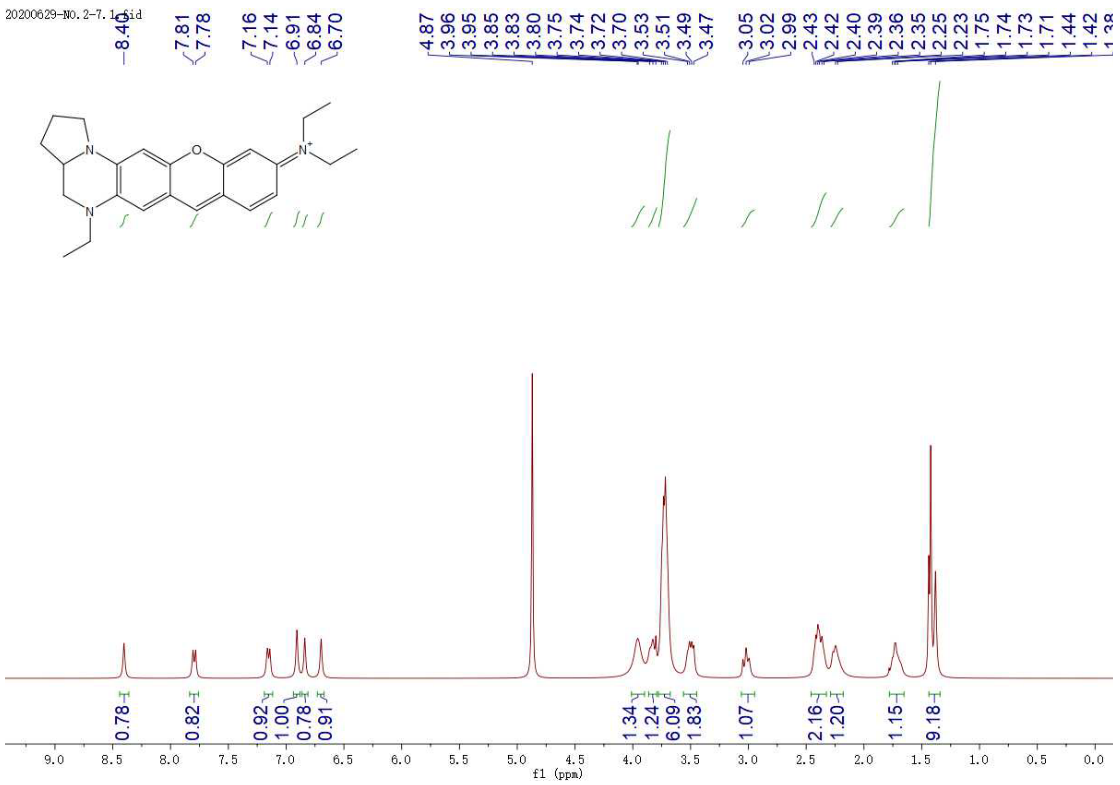

^{1}H NMR spectrum of **18** in CD_3OD containing 20 % $CDCl_3$

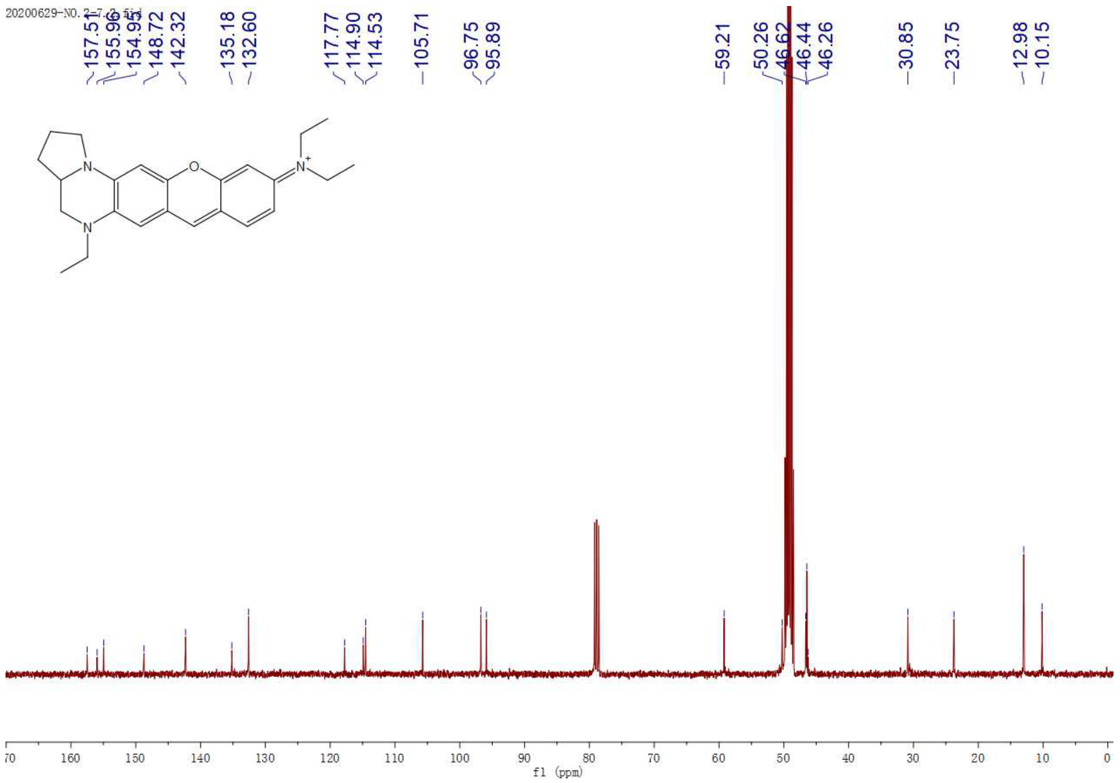

^{13}C NMR spectrum of **18** in CD_3OD containing 20 % $CDCl_3$

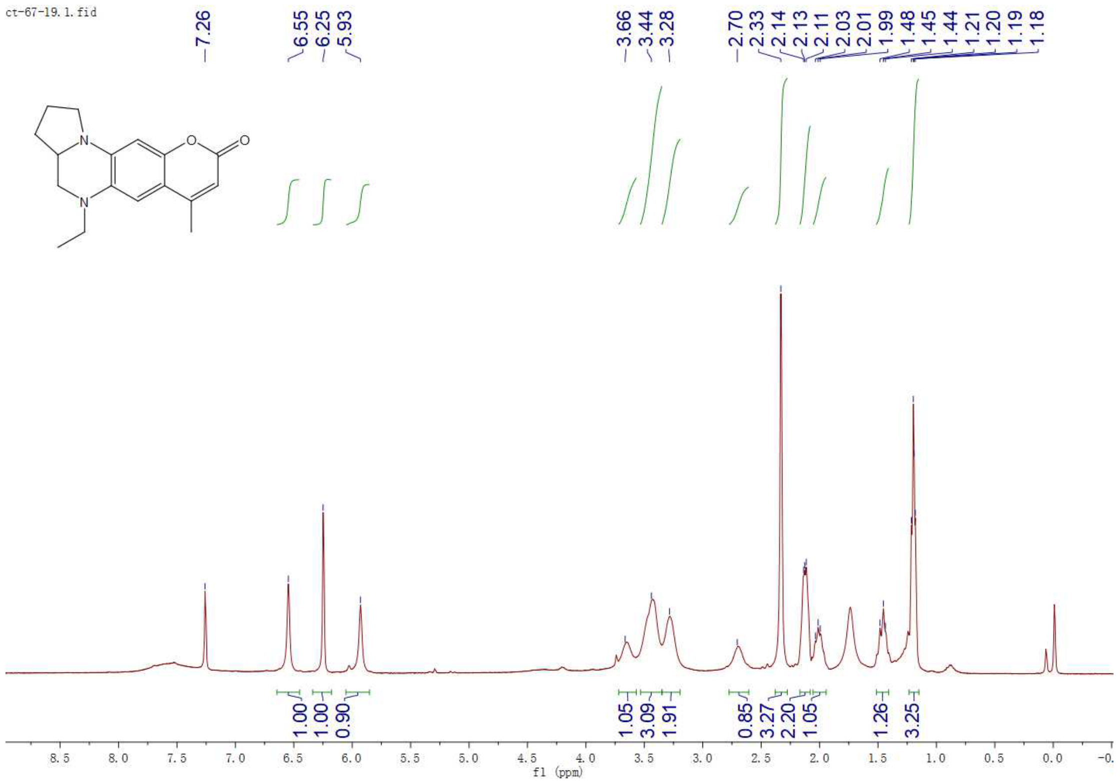

^{1}H NMR spectrum of **19** in $CDCl_3$

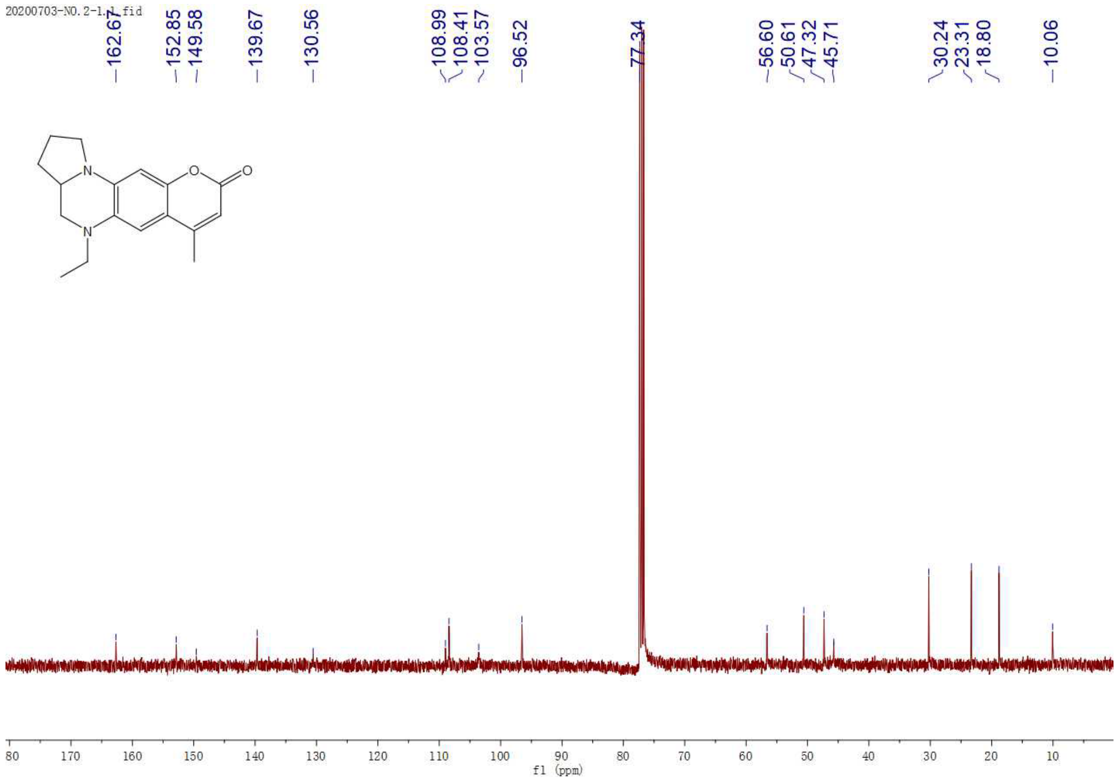

^{13}C NMR spectrum of **19** in $CDCl_3$

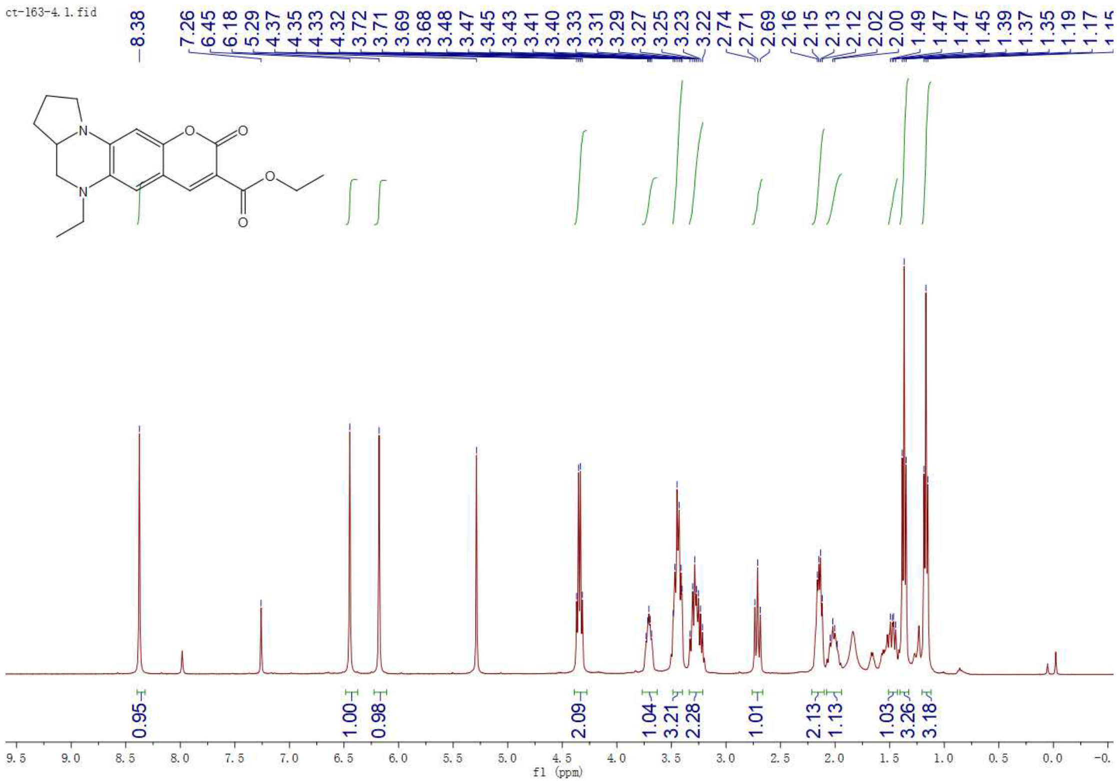

^{1}H NMR spectrum of **20** in $CDCl_3$

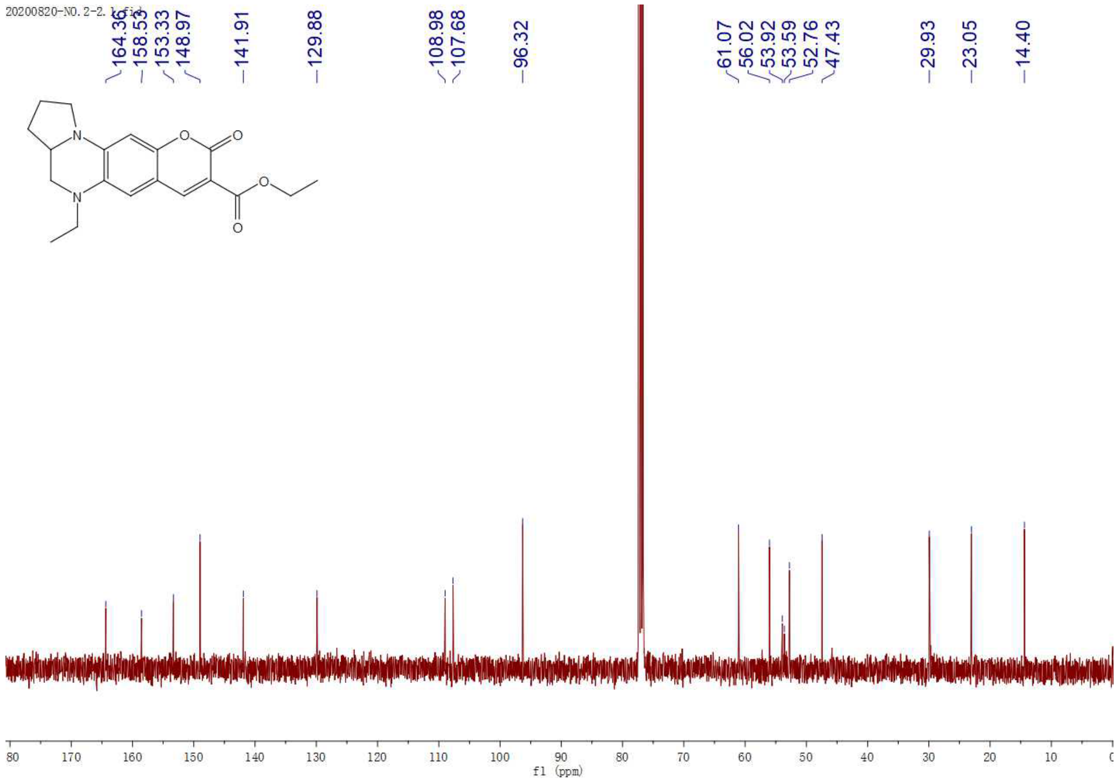

^{13}C NMR spectrum of **20** in $CDCl_3$

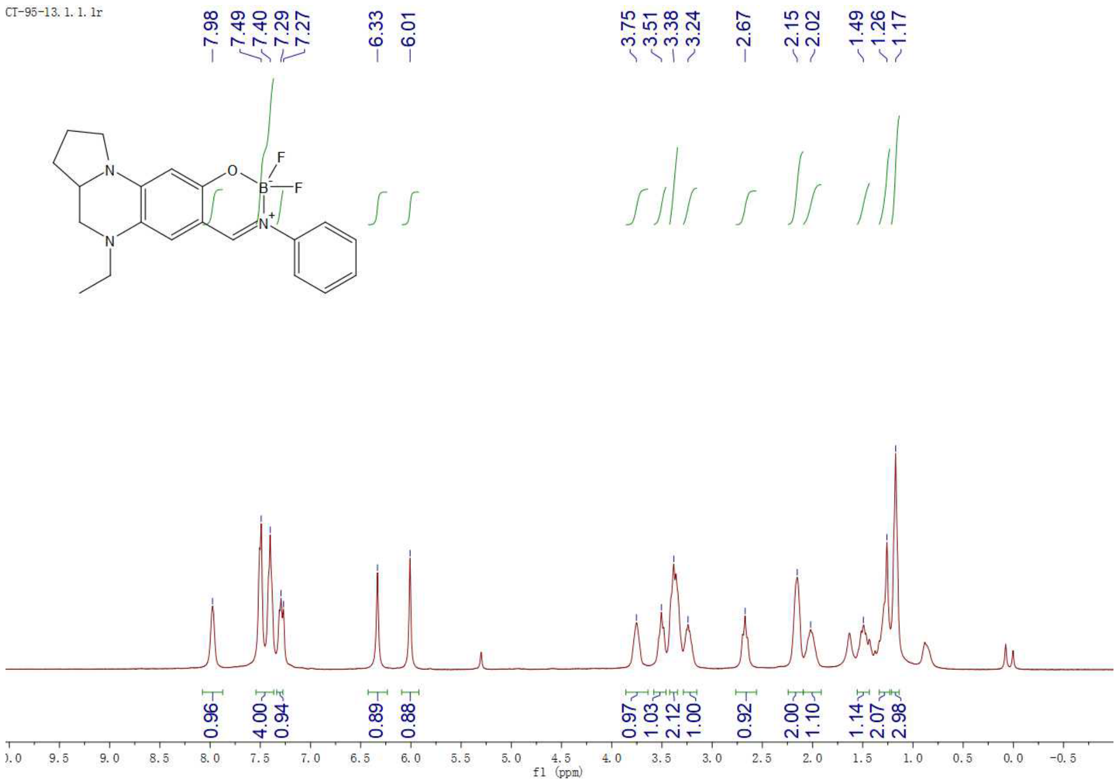

^{1}H NMR spectrum of **21** in $CDCl_3$

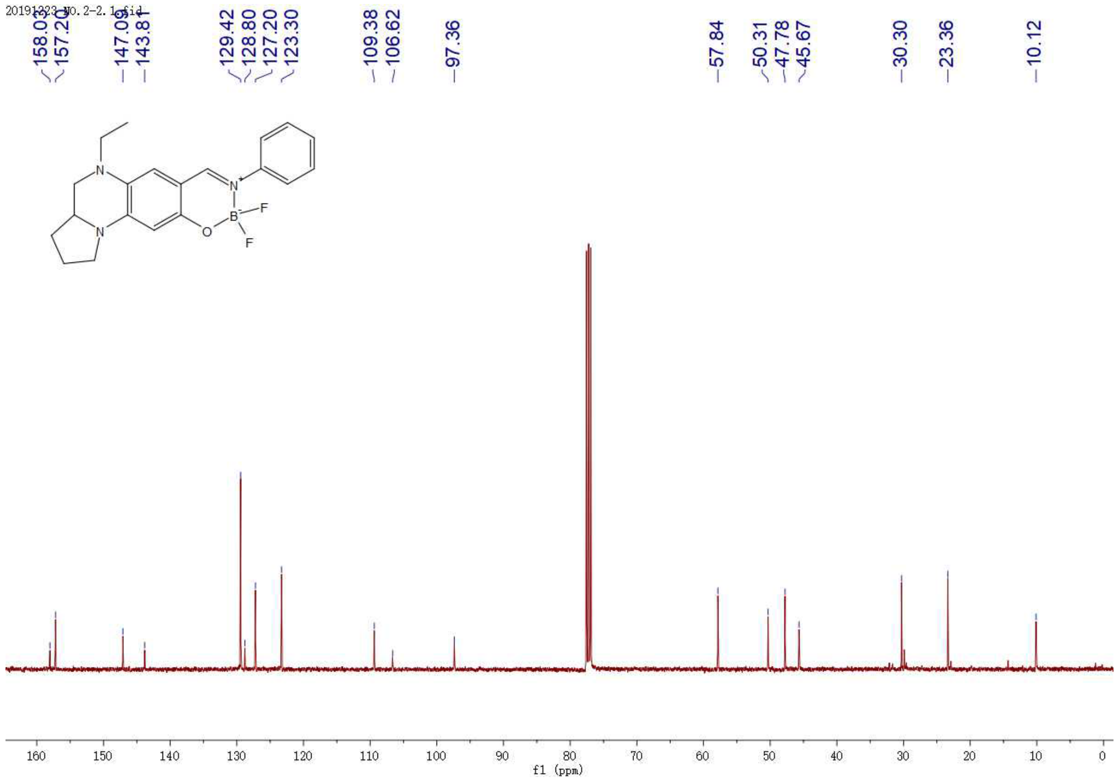

^{13}C NMR spectrum of **21** in $CDCl_3$

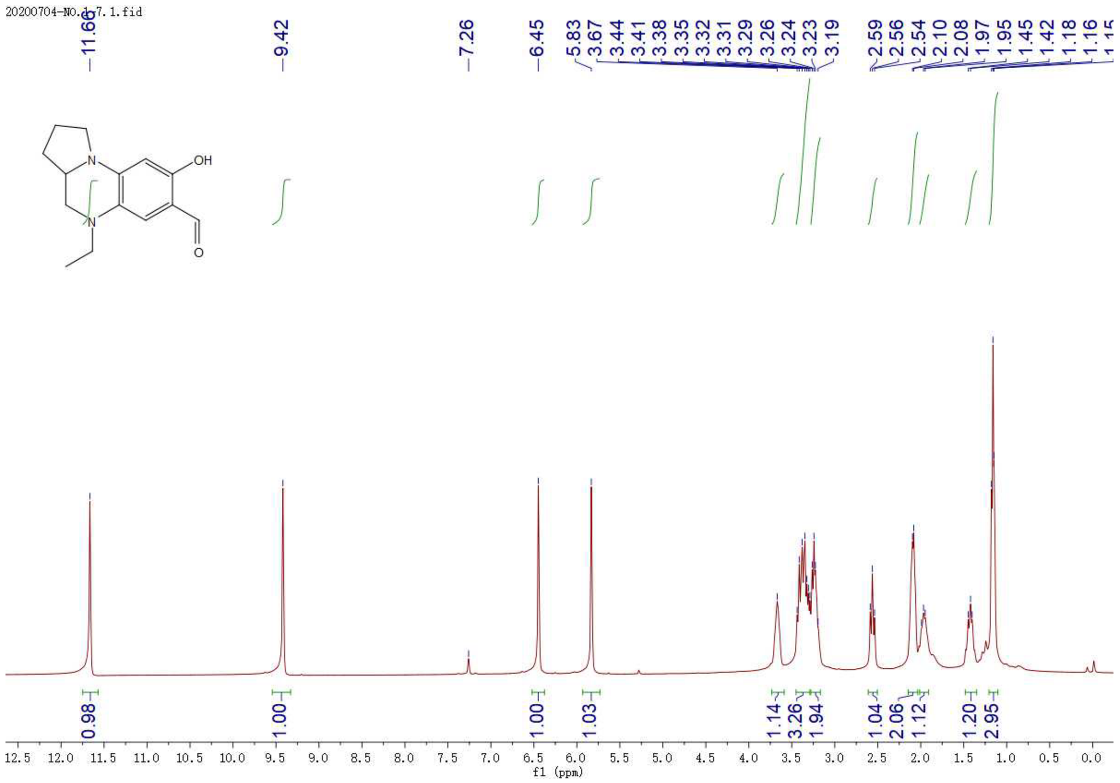

^{1}H NMR spectrum of **S-20** in $CDCl_3$

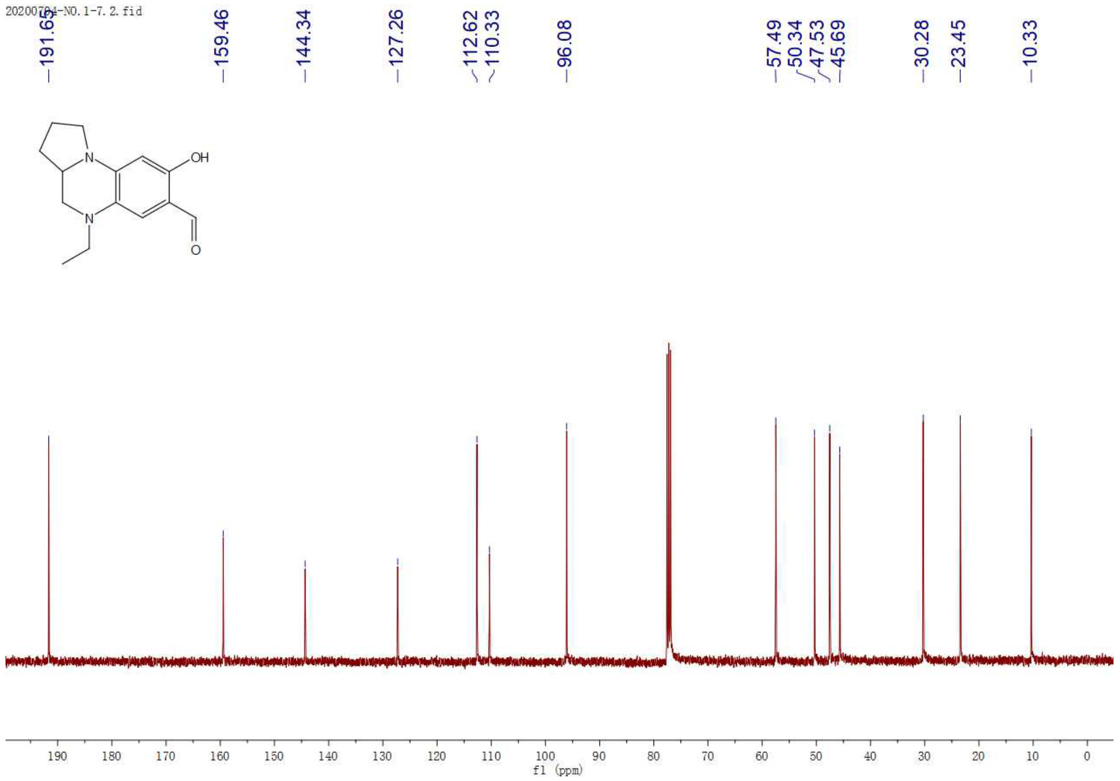

^{13}C NMR spectrum of **S-20** in $CDCl_3$

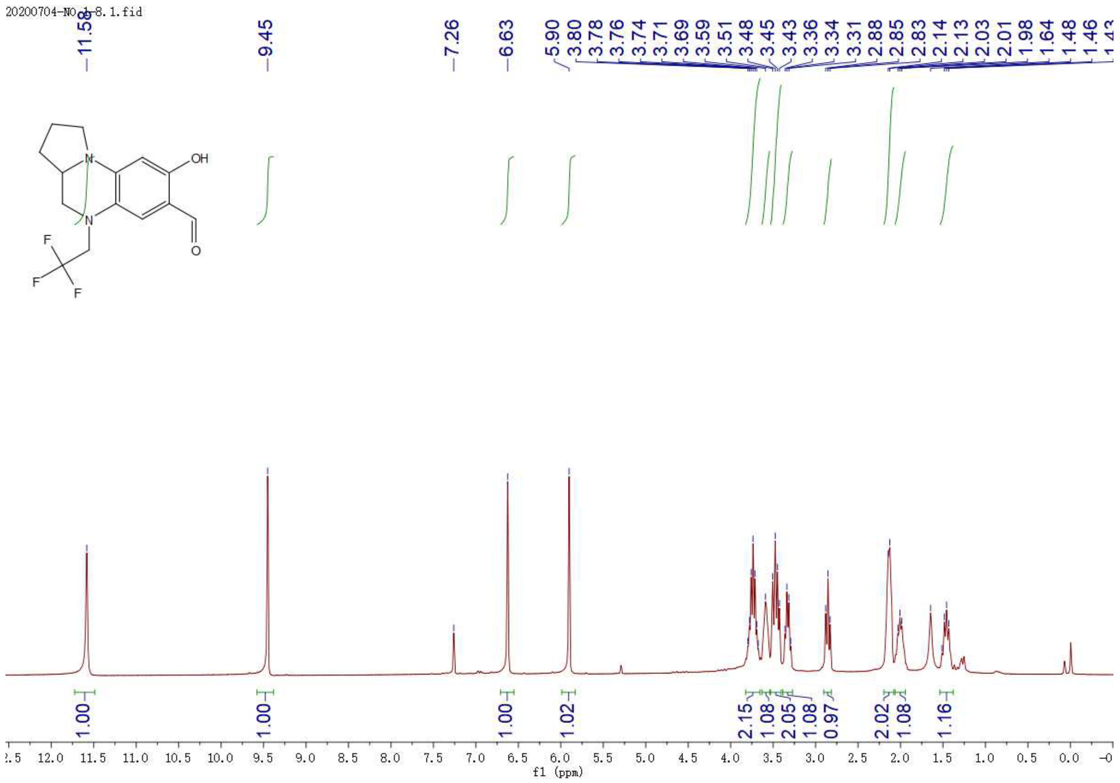

^{1}H NMR spectrum of **S-21** in $CDCl_3$

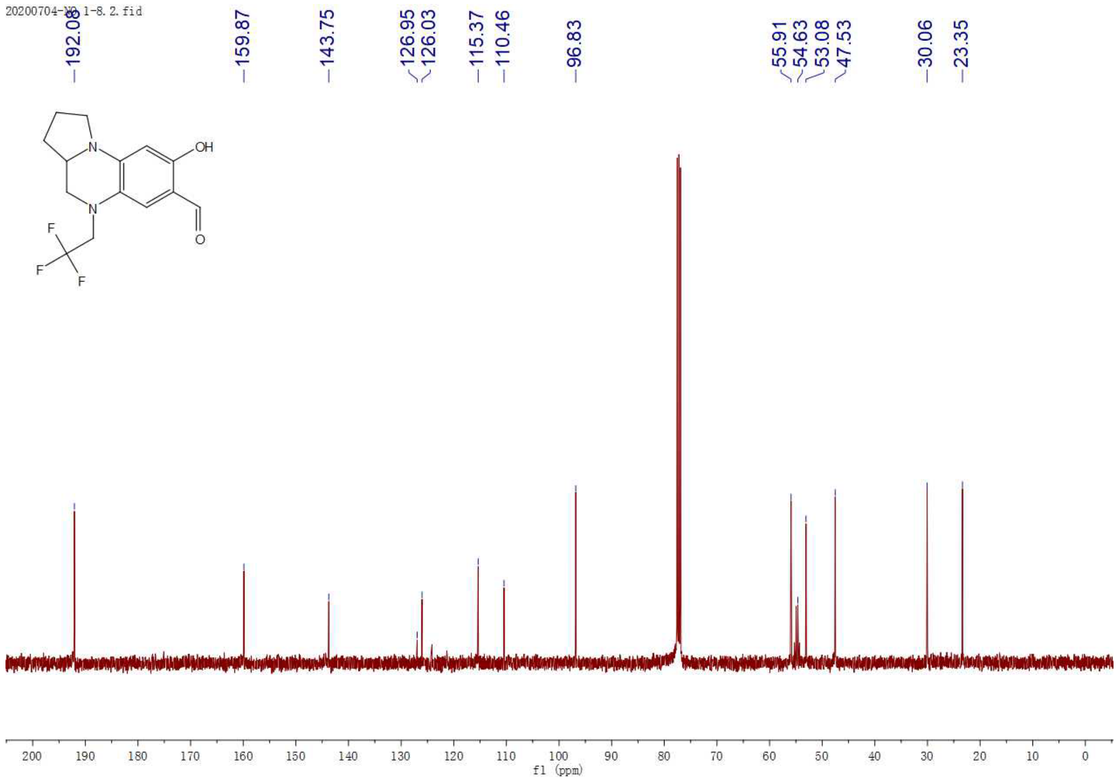

^{13}C NMR spectrum of **S-21** in $CDCl_3$

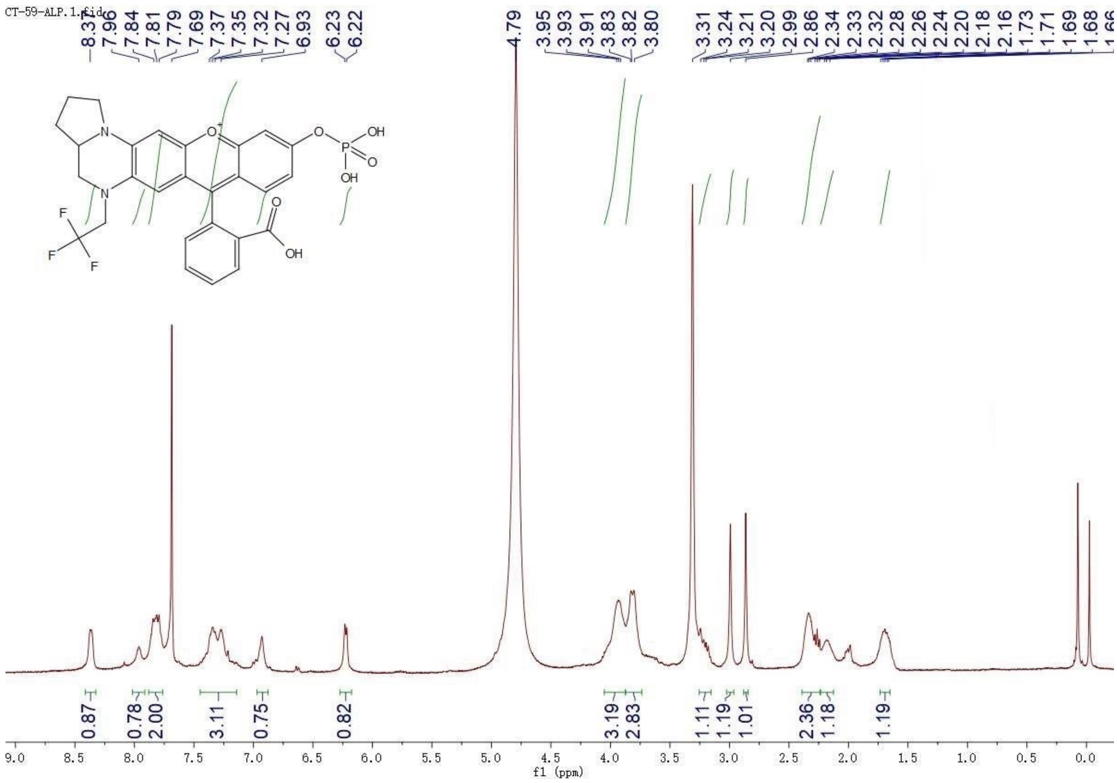

^{1}H NMR spectrum of **11-ALP** in CD_3OD containing 20 % $CDCl_3$

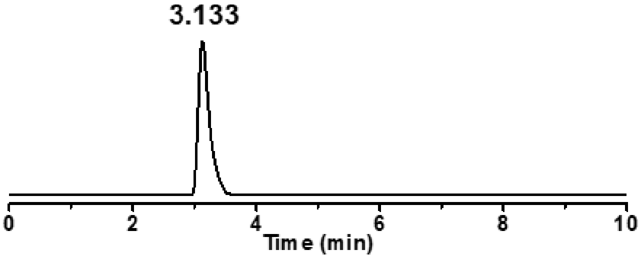

HPLC spectrum of **11-ALP**

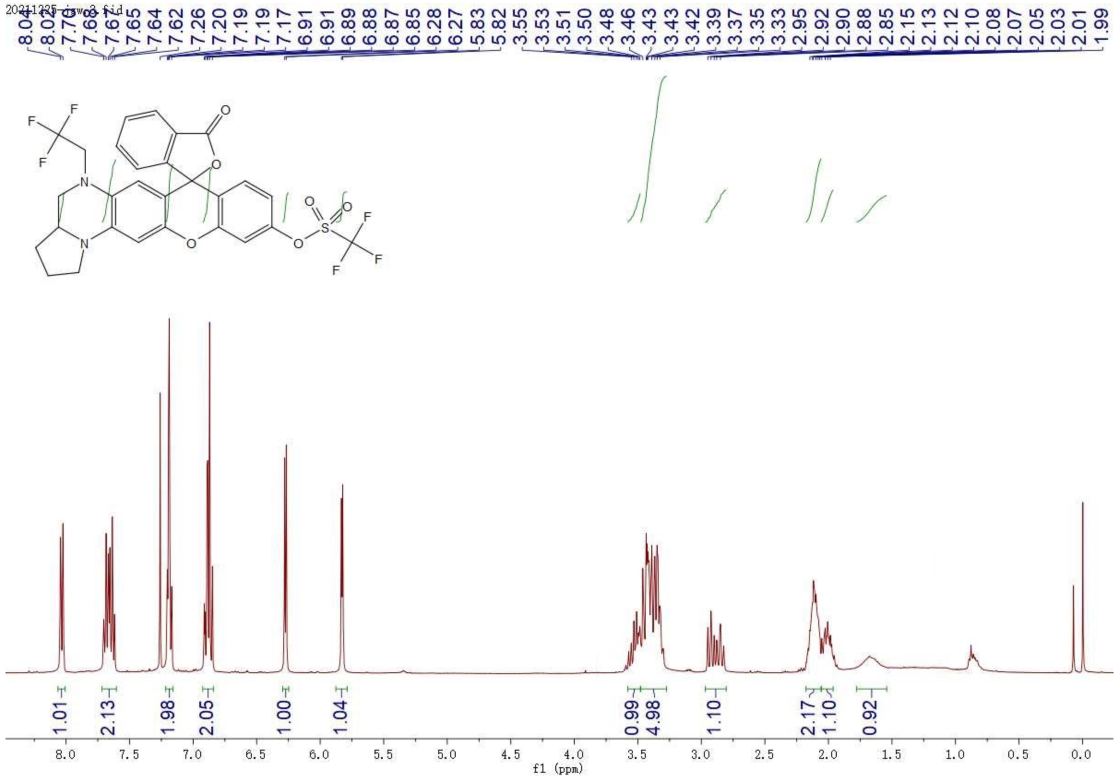

^{1}H NMR spectrum of **S-31** in $CDCl_3$

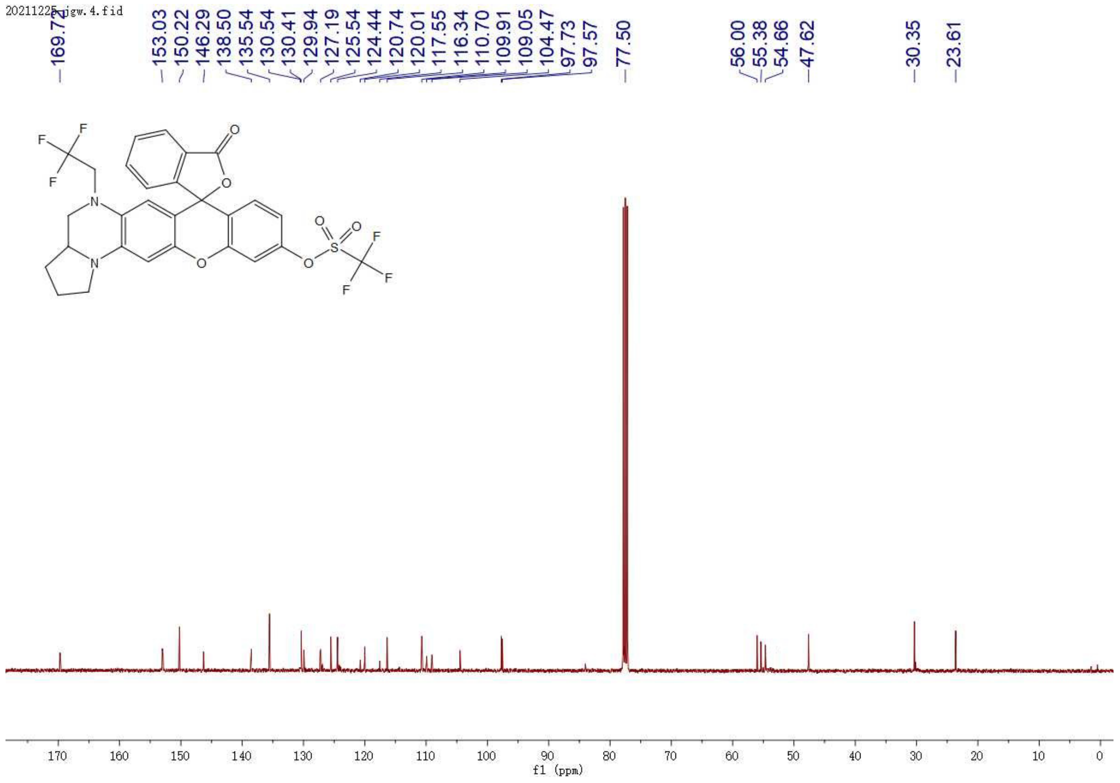

^{13}C NMR spectrum of **S-31** in $CDCl_3$

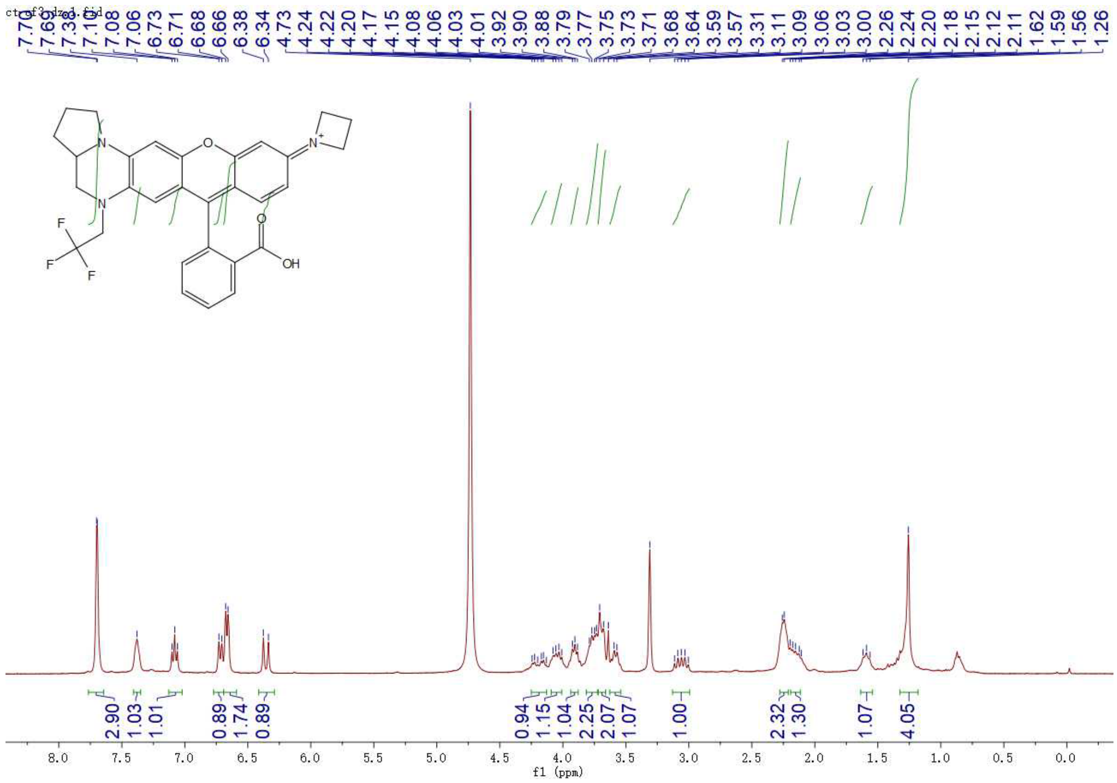

^{1}H NMR spectrum of **YL-Az** in CD_3OD

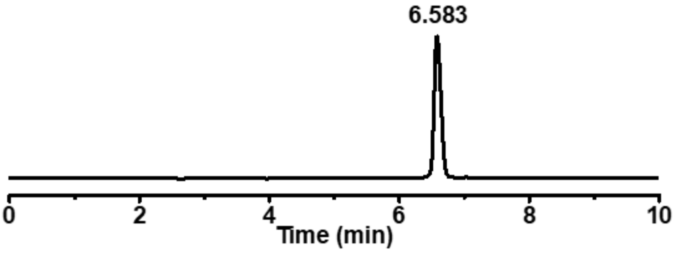

HPLC spectrum of **YL-Az**

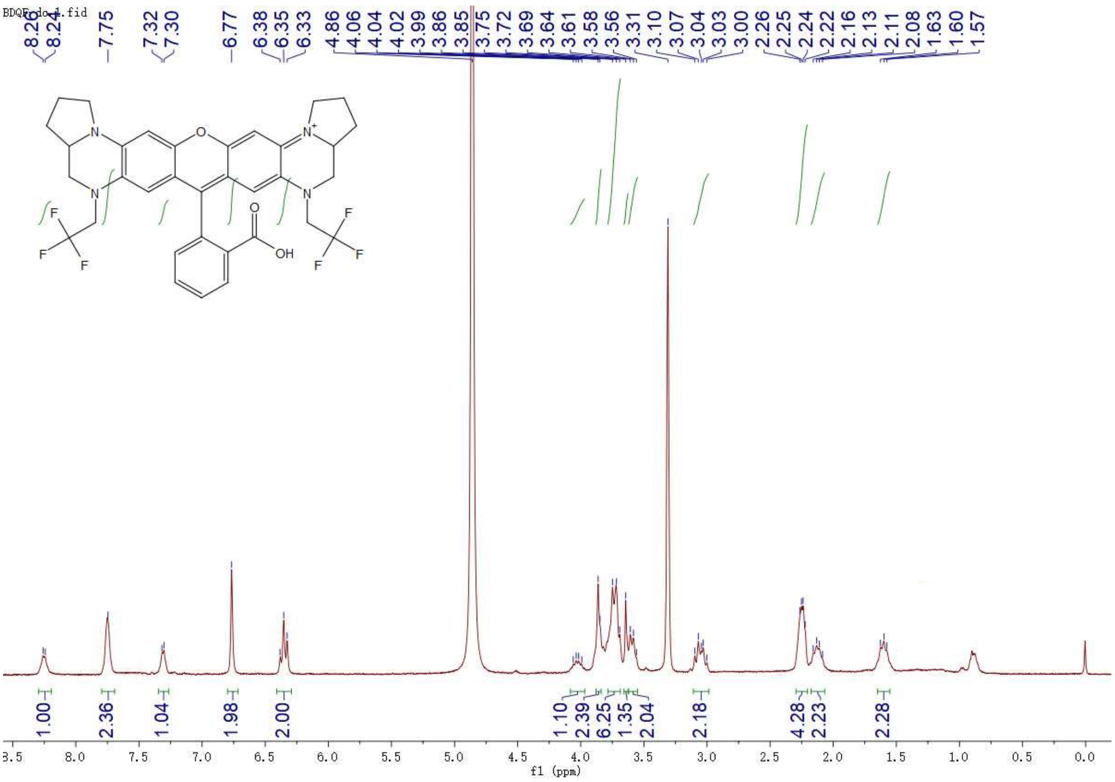

^{1}H NMR spectrum of **bis-YL** in CD_3OD

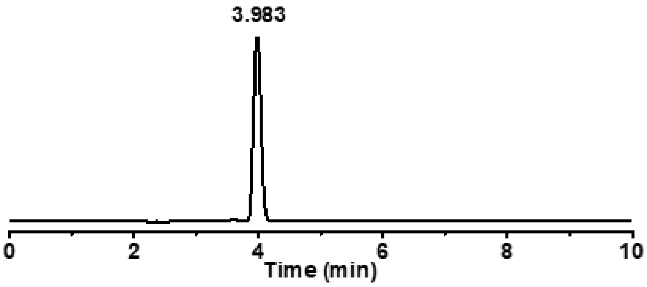

HPLC spectrum of **bis-YL**

References

1. Zheng Q., Lavis L. D. Development of photostable fluorophores for molecular imaging. *Curr. Opin. Chem. Biol.* **39**, 32-38 (2017).
2. Grimm J. B.*, et al.* A general method to improve fluorophores for live-cell and single-molecule microscopy. *Nat. Methods* **12**, 244-250 (2015).
3. Butkevich A. N., Bossi M. L., Lukinavicius G., Hell S. W. Triarylmethane fluorophores resistant to oxidative photobluing. *J. Am. Chem. Soc.* **141**, 981-989 (2019).
4. Hansch C., Leo A., Raft R. W. A survey of hammett substituent constants and resonance and field parameters. *Chem. Rev.* **91**, 165-195 (1991).
5. Chen W., Xu S., Day J. J., Wang D., Xian M. A general strategy for development of near-infrared

fluorescent probes for bioimaging. *Angew. Chem. Int. Ed.* **56**, 16611-16615 (2017).

6. Rurack K., Spieles M. Fluorescence quantum yields of a series of red and near-infrared dyes emitting at 600-1000 nm. *Anal. Chem.* **83**, 1232-1242 (2011).
7. Kubin R. F., Fletcher A. N. Fluorescence quantum yields of some rhodamine dyes. *J. Lumin.* **27**, 455-462 (1982).
8. Liu X., *et al.* Methylated chromenoquinoline dyes: synthesis, optical properties, and application for mitochondrial labeling. *Chem. Commun.* **54**, 1509-1512 (2018).
9. Zhou W., Fang X., Qiao Q., Jiang W., Zhang Y., Xu Z. Quantitative assessment of rhodamine spectra. *Chinese Chem. Lett.* **32**, 943-946 (2021).
10. Zhang X., Zhang J., Lu X. The fluorescence properties of three rhodamine dye analogues: acridine red, pyronin Y and pyronin B. *J. Fluoresc.* **25**, 1151-1158 (2015).
11. Peng T., Yang D. Construction of a library of rhodol fluorophores for developing new fluorescent probes. *Org. Lett.* **12**, 496-499 (2010).
12. Frisch M. J., *et al.* Gaussian 09, Revision A.1; Gaussian, Inc.: Wallingford, CT. (2009).
13. Yanai T., Tew D. P., Handy N. C. A new hybrid exchange-correlation functional using the coulomb-attenuating method (CAM-B3LYP). *Chem. Phys. Lett.* **393**, 51-57 (2004).
14. Critchfield F. E., Gibson J. A., Hell J. L. Dielectric constant for the dioxane water system from 20 to 35°. *J. Am. Chem. Soc.* **75**, 1991-1992 (1953).
